# The Biosynthetic Gene Cluster of Boydines in *Scedosporium apiospermum*

**DOI:** 10.1007/s11046-026-01050-z

**Published:** 2026-02-07

**Authors:** Clarisse Carvalho, Anaïs Hérivaux, Méline Wéry, Jean-Charles Jouhanneau, Nicolas Papon, Jean-Philippe Bouchara

**Affiliations:** 1 IRF (Infections Respiratoires Fongiques), Univ Angers, Univ Brest, SFR ICAT 4208, Angers, France; 2 Univ Angers, SFR ICAT 4208, Angers, France

**Keywords:** *Scedosporium*, Boydines, Acetylaranotin, Aspirochlorine, Biosynthetic gene cluster, Genome mining

## Abstract

Although *Scedosporium* species may cause severe infections in immunocompromised patients, little is known about their pathogenic mechanisms. The thioredoxin reductases (TrxRs) of *Scedosporium apiospermum* are thought to play an important role in protecting the fungus against oxidative stress. The genes that encode these proteins are part of biosynthetic gene clusters (BGCs) which ensure the synthesis of non-ribosomal peptides. Due to the discrepancies between chemical studies and bioinformatic predictions regarding the product of the BGC comprising the TrxR-encoding gene *SAPIO_CDS1830*, a large-scale study of almost 300 fungal genomes was undertaken to search for BGCs that could potentially synthesize homodipeptides. Phylogenetic analysis of the amino acid sequence of the adenylation domain of a large number of non-ribosomal peptide synthases confirmed the assembly of two phenylalanine molecules. Synteny analysis clearly showed that this BGC ensures the synthesis of some aranotin-related hybrid compounds, called boydins. This BGC comprised 15 genes, including one encoding a polyketide synthase, which allows the synthesis of the polyketide chain attached to the dipeptide skeleton. The orthologues of all members of this BGC were also identified in all available *Scedosporium* genomes, as well as in other Sordariomycetes and even in some phylogenetically distant molds living as endophytes or plants pathogens, or on decaying wood. These secondary metabolites, which may therefore not be specific to the *Scedosporium* genus, could play a role in evasion of the fungus to the oxidative stress, as suggested by the overexpression of several members of this BGC in response to oxidative stress.

**Supplementary Information:**

The online version contains supplementary material available at 10.1007/s11046-026-01050-z.

## Introduction

*Scedosporium* species are soil filamentous fungi which may cause a large variety of infections in humans, ranging from localized infections, such as subcutaneous mycetomas and bone and joint infections, resulting from traumatic inoculation of fungal elements, to respiratory infections thought to be due to the inhalation of airborne fungal spores [1]. The latter, which are close to *Aspergillus* infections, include unilateral sinusitis, pulmonary mycetoma similar to the well-known aspergilloma, bronchitis, and invasive pulmonary infections in patients with AIDS, chronic granulomatous disease or hematological malignancies, as well as in solid organ transplant recipients [2]. Patients with cystic fibrosis (CF) are particularly prone to *Scedosporium* infections since these microorganisms rank second among the filamentous fungi colonizing the CF airways, after *Aspergillus fumigatus*. While this fungal colonization is usually well tolerated, it can sometimes lead to febrile pneumopathies, allergic bronchopulmonary mycoses, or even deep-seated or disseminated infections in case of corticosteroid-induced diabetes or after lung or heart–lung transplantation. Nevertheless, little is known about the pathogenic attributes of these fungi [1].

*Scedosporium* species must face many challenges in the environment as well as in the respiratory tract of CF patients. They must protect themselves from oxygen radicals, and combat surrounding bacteria and fungi. Acquiring the extracellular iron is also challenging since this essential metal for fungal growth is abundant in the environment but not in a form that can be directly assimilated. By allowing the synthesis of compounds with antiradical, antibacterial, antifungal or chelating properties, secondary metabolism is of particular interest, especially since it has no equivalent in mammalian cells and therefore represents potential therapeutic targets. The importance of secondary metabolism in *Scedosporium* species has recently been highlighted [3]. For example, some secondary metabolites produced through non-ribosomal peptide (NRP) synthesis are involved in the acquisition of extracellular iron [4]. Indeed, the non-ribosomal peptide synthase (NRPS) SidD encoded by the *SAPIO_CDS2806* gene is essential for iron uptake, and disruption of the gene encoding this enzyme resulted in a lack of growth under iron-restricted culture conditions and loss of virulence in a murine model of disseminated scedosporiosis. Nevertheless, other secondary metabolites could also play a role in the pathogenicity of *Scedosporium* species.

Recent studies suggested an important role for thioredoxin reductases (TrxRs) in protecting the fungus against oxidative stresses [5]. Of note, the two corresponding genes are also part of biosynthesis gene clusters (BGCs) involved in the synthesis of some NRPs [6]. Although these enzymes may play a direct role in evasion of the fungus to the host immune response by reducing oxidized thioredoxins, an indirect role via the synthesis of secondary metabolites with immunomodulatory properties should also be considered.

As mentioned above, the *Scedosporium* TrxRs-encoding genes are part of two BGCs centered on an NRPS-encoding gene and potentially involved in the synthesis of some epidithiodioxopiperazines (ETPs), whose role in pathogenicity has been widely demonstrated in other fungal models [6]. ETPs result from the condensation of two identical or non-identical amino acids by a head-to-head assembly through the activity of some NRPSs. In addition, synthesis of these metabolites requires the presence of a TrxR-encoding gene within the BGC, responsible for the formation of a disulfide bridge which supports their immunomodulatory properties [7, 8]. For example, gliotoxin, which is largely recognized as a major pathogenic determinant in *A. fumigatus*, inhibits the immune response mediated by cytotoxic T cells and phagocytosis, and stimulates apoptosis of monocytes and dendritic cells [9–17]. Similar properties have also been reported for other ETPs, like sporidesmin [7, 9, 18].

In *S. apiospermum*, the nature of the ETPs produced by these two BGCs remains controversial. Among the nearly 170 secondary metabolites identified by chemical analyses on different strains of the *Scedosporium* genus, 12 compounds exhibit a structure reminiscent to that of the ETP aranotin produced by *Aspergillus terreus,* another filamentous fungus associated with CF [19, 20]. However, previous analysis of the genome of *S. apiospermum* using the bioinformatic tool antiSMASH suggested that aspirochlorine is synthesized by the BGC centered on the *SAPIO_CDS1828* gene, while only aranotin derivatives have been described to date for *Scedosporium* species [21, 22]. The first steps in the biosynthetic pathway of these two ETPs are identical since both compounds derive from the assembly of two phenylalanine building blocks, and it is only after the formation of the disulfide bridge that the two pathways diverge with: (i) the production of a regularly branched ETP for the acetylaranotin biosynthesis pathway in *A. terreus* [19, 20]; or (ii) the migration of the disulfide bridge from α,α′- to α,β′-positions and spiroformation, followed by a retro-aldol reaction resulting in the conversion of one of the phenylalanyl residues into a glycyl residue, for the aspirochlorine biosynthesis pathway in the non-pathogenic *Aspergillus oryzae* [23, 24]. Since our previous studies on *S. apiospermum* IHEM 14462 [6], other genomes were made available for the *Scedosporium* genus. Therefore, we undertook a broad scale bioinformatic study browsing about 300 genomes of filamentous fungi (including the available *Scedosporium* genomes) to clarify the nature of the metabolites produced by this BGC.

## Materials and Methods

### Genome Mining

In order to clarify the nature of the ETPs produced by the BGC centered on *SAPIO_CDS1828* in *S. apiospermum*, we first conducted a bibliographic search focused on fungal species known to produce acetylaranotin, aspirochlorine, and related compounds. In addition, we identified fungal species harbouring orthologs of *S. apiospermum SAPIO_CDS1828* (encoding the NRPS KEZ45502) through a BLAST search (Basic Local Alignment Search Tool) using the BLASTp algorithm (available at https://blast.ncbi.nlm.nih.gov/Blast.cgi). Fungal species with orthologs of *A. terreus ATEG_03470* (encoding the AtaP NRPS – GenBank identifier: EAU36744—responsible for the first step in acetylaranotin synthesis in *A. terreus* strain NIH 2624) and *A. oryzae AO090001000043* (encoding the AclP NRPS—GenBank identifier: BAE56606—responsible for the first step in aspirochlorine synthesis in *A. oryzae* strain RIB 40) were also identified.

The genomes of these species were then analysed using the antiSMASH fungal version 7.0 pipeline [25]. In our study, if multiple genomes were found available for a fungal species, priority was given to the reference genome when it was annotated. Otherwise, the analysis was performed using an annotated genome from another strain, or, as a last resort, with the genomic data from the reference genome (Supplementary Material Table S1).

NRPSs are large multimodular proteins. Each module comprises at least an adenylation domain (the A domain responsible for the activation of the amino acid), a thiolation domain with a phosphopantetheine group attached to a seryl residue (the PP domain responsible for elongation of the peptide chain), and a condensation domain (the C domain which catalyzes the condensation of amino acids). These megaenzymes comprise as many modules as there are amino acids in the synthesized peptide. Nevertheless, for peptides resulting from the assembly of two identical amino acids, a single adenylation domain is required. Thus, results from antiSMASH analysis were screened for BGCs displaying a core gene encoding an NRPS with a single adenylation domain and two condensation domains, therefore, potentially responsible for the synthesis of homodipeptides. A dataset was built comprising the GenBank accession numbers of the NRPS and of the corresponding gene, the species name, the domain architecture of the NRPS, the amino acid predicted to be recognized, and the metabolite predicted to be synthesized. In addition, the presence of a PKS-encoding gene in the BGC was also compiled (Supplementary Material Table S2). Substrate specificity of the adenylation domains was further investigated using the recently developed algorithm PARAS v1.0.1 (https://paras.bioinformatics.nl/submit) which takes into account a pre-defined list of 223 substrates [26]. Then, a phylogenetic tree was constructed using IQ-TREE software version 3.0 from the comparison of the amino acid sequences of the A domain of these NRPSs. The peptide sequence of the NRPS encoded by *SAPIO_CDS2806*, erroneously considered as a pseudogene in the annotation of the reference genome because of the lack of introns [4], was determined using ExPASy translate (https://web.expasy.org/translate/), and after analysis of the deduced protein sequence with InterProScan (https://www.ebi.ac.uk/interpro/search/sequence/), the peptide sequence of the A domain was included in the phylogenetic analysis.

The orthologs of the different genes that are part of the acetylaranotin BGC in *A. terreus* genome, or the aspirochlorine BGC in *A. oryzae* genome, were searched in the reference genome of *S. apiospermum* using the BLASTp or tBLASTn algorithms since the reference genome comprises about 2000 intronless coding sequences considered as pseudogenes [27]. Similarly, orthologs of the different coding sequences in the “aspirochlorine” BGC in *S. apiospermum* reference genome were searched in the reference genomes of *A. terreus* and *A. oryzae* using the BLASTp or BLASTx algorithms.

Protein sequences were analyzed using InterProScan and ScanProsite to predict the putative domains and motifs, and the biological functions of these proteins. Peptide sequences of putative efflux pumps were analyzed using the PSI-Blast algorithm (https://tcdb.org/), the Protscale bioinformatic tool (https://web.expasy.org/protscale/) with the transmembrane tendency scale, and the HMMTOP (http://www.enzim.hu/hmmtop/server/hmmtop.cgi), CCTOP (https://cctop.ttk.hu), and WHAT (Web-based Hydropathy, Amphipathicity and Topology; https://tcdb.org/biotools/what.php) algorithms.

PKSs are megaenzymes catalyzing the synthesis of polyketides by assembling malonyl-CoA condensation units, after initiation of the process with an acetyl-CoA starter unit. Type 1 PKSs are multidomain proteins comprising: (i) an acyltransferase (AT) domain, which recognizes the building block to be used; (ii) a keto synthase (KS) domain, which catalyzes C–C bond formation; and (iii) a thiolation domain with a phosphopantetheine group attached to a seryl residue (PP), which allows the binding of the growing polyketide chain as a thioester. In an attempt to determine the number of condensation units assembled by the identified PKSs, the peptide sequences of their PP domain were compared using MEGA XI software with those of PKSs known to perform the assembly of a well-defined number of malonyl-CoA units, *i.e.* fungal PKSs involved in the synthesis of mellein (4 malonyl-CoA units), betaenone A (7), solanapyrones (7) or monacolin K (8), or of the alkyl chain of asperfuranone (3) or fumagillin (5) (Supplementary Material Table S3).

Some PKSs show additional domains, including a keto reductase (KR) domain catalyzing the reduction of all or part of the carbonyl groups, a dehydratase (DH) domain catalyzing the loss of all or some of the hydroxyl groups resulting from the reduction of the carbonyl groups, a carboxymethyl transferase (cMT) domain catalyzing the introduction of an additional methyl group during elongation of the polyketide chain, and an enoyl-reductase (ER) domain catalyzing the reduction of all or part of the C–C double bonds resulting from dehydration. The peptide sequence of the ER domain of the PKSs identified for *Scedosporium* species was compared with those of type1-PKSs with a functional ER domain, involved in the synthesis of zearalenone or alternapyrone, or of the alkyl chain of asperfuranone, fumonisin or cylodepsipeptides like BII-rafflesfungin, emericellamide, W493 B/ W493 A and beauveriolides (Supplementary Material Table S4). Peptide sequences were multialigned with MEGA XI and signature logos were determined with Seq2logo—2.0 (https://services.healthtech.dtu.dk/services/Seq2Logo-2.0/).

Finally, due to the discrepancies in the amino acid sequences of the protein KEZ45502 encoded by the *SAPIO_CDS1824* gene and its orthologs in the fungal kingdom, the nucleotide sequence of *SAPIO_CDS1824* was analyzed using GENSCAN (http://hollywood.mit.edu/GENSCAN.html) and the amino acid sequence of KEZ45502 was analyzed with PANNZER2 (http://ekhidna2.biocenter.helsinki.fi/sanspanz/) [28].

### DNA Extraction, PCR and Sequencing

Genomic DNA was extracted from fresh mycelium from cultures grown for 9 days on potato-dextrose-agar. The mycelium was ground using a mini-bead beater in 10 mM Tris–HCl lysis buffer (pH 8) supplemented with 1 mM EDTA (TE buffer), 2% Triton X100, 1% SDS, and 0.1 M NaCl. Genomic DNA was extracted by the addition of an equal volume of phenol:chloroform:isopropanol (25:24:1; Sigma-Aldrich, Saint-Quentin-Fallavier, France). RNA was then digested with 0.2 mg/mL RNase A. DNA was then precipitated by adding an equal volume of isopropanol. After washing with 70% ethanol, DNA was resuspended in TE buffer and quantified using a Qubit® 2.0 Fluorometer (Invitrogen, Cergy-Pontoise, France). The DNA integrity was checked by 1% agarose gel electrophoresis, and the genomic DNA was then stored at 4 °C.

When necessary, appropriate primers were designed to check for chromosome rearrangements or to join some contigs and reconstruct the BGCs (Supplementary Material Table S5). PCR was performed in a final volume of 50 μL containing 10 µL 5X Q5 reaction buffer, 10 µL 5X Q5 high-GC enhancer, 1.25 µL of 10 mM dNTPs, 25 pmol of each primer, 1.25 μL of Q5 high-fidelity DNA polymerase (2,000 U/mL) (New England Biolabs, Evry, France), and 2 μL of DNA extract. The PCR cycling parameters included an initial denaturation step at 95 °C for 5 min, followed by 35 cycles of denaturation at 95 °C for 30 s, annealing at 62 °C for 30 s, and extension at 72 °C for 2 min, with a final elongation step at 72 °C for 5 min. The amplified products were then purified on gel using the NucleoSpin Clean-up Kit (Macherey–Nagel, Düren, Germany) and sent to Eurofins Genomics (Ebersberg, Germany) for sequencing.

### Quantification of Gene Expression Levels

Gene expression in cultures grown under standard or hypercapnic culture conditions in yeast extract-peptone-dextrose (YPD) broth, or in a synthetic medium mimicking the CF bronchial mucus (CFSM), was quantified by RNA-seq. All procedures, from total RNA extraction and DNA digestion, to sequencing and transcript assembly and quantification, were previously reported [29].

The gene expression level was also analyzed under oxidative stress conditions. Germination was initiated by incubating conidia in YPD broth for 24 h. Afterward, the hyphae were transferred to fresh YPD broth with or without 1 mM menadione or 2 mM cumene hydroperoxide (Sigma Aldrich). After 3 h of incubation, the fungal cells were harvested and ground in liquid nitrogen with a mortar and a pestle. Total RNA was recovered using the NucleoSpin® RNA Plant kit (Macherey–Nagel) according to the manufacturer's instructions. After digestion of DNA using the rDNase Set (Macherey–Nagel), RNA was quantified by fluorometry and reverse transcribed using the High Capacity RNA-to-cDNA kit (Applied biosystems, Foster City, CA). cDNAs were stored at − 20 °C until use.

cDNAs were used as a template for quantitative PCR (qPCR). Each reaction (12.5 μL final volume) contained PowerUp™ SYBR™ Green Master Mix (Applied Biosystems), 5 or 10 pmol of each primer depending on the amplified gene (Supplementary Table S5), and 2 μL of fivefold diluted cDNA. qPCR reactions were carried out on a StepOnePlus™ thermocycler (Applied Biosystems) with the following thermal profile: 2 min at 50 °C and 2 min at 95 °C, followed by 40 cycles of 3 s at 95 °C and 30 s at 60 °C. Melting curve analysis was performed immediately after the amplification procedure as follows: 95 °C for 15 s, and a stepwise annealing process from 60 °C to 94.9 °C with 0.3 °C increments. Fold changes in the gene expression level relative to the standard condition (*i.e*., YPD broth) were calculated in each condition using the delta-delta Ct method with the actin gene as the endogenous control [30]. For each data point, two biological replicates and two technical replicates were performed, and the variation in expression of a given gene was considered significant if the log_2_ fold change was > 1 or <  − 1.

## Results

### The NRPS KEZ45505 Encoded by *SAPIO_CDS1828* Ensures the Synthesis of Cyclo(Phe-Phe)

Our bibliographic and BLAST searches using KEZ45505, EAU36744 or BAE56606 as a query yielded a list of 294 fungal species (Supplementary Material Table 1). AntiSMASH analysis of the available genomes (n = 288) allowed us to identify 719 proteins with a single adenylation domain and two condensation domains, corresponding to 278 fungal species (Supplementary Table 2). The domain architecture A-PP-C1-PP-C2 was inferred for 256 of these proteins, which were predicted to be involved in the synthesis of metachelin-type siderophores or dimethylcoprogen for 106 and 37 proteins, respectively, whereas the domain architectures PP-C1-A-PP-C2 and C1-A-PP-C2 were inferred for 243 and 128 proteins, respectively. For 270 proteins, antiSMASH predicted the recognition of phenylalanine by the adenylation domain (substrate specificity confirmed using PARAS v1.0.1 for all but four of these NRPSs), whereas the predicted substrates were alanine and tryptophan for 29 and 21 proteins, respectively (2S-aminododecanoic acid and tryptophan for 16 and 19 of these proteins, respectively, using PARAS v1.0.1). Regarding the metabolites potentially produced, the most frequently predicted were aspirochlorine, acetylaranotin and penigainamide/outovirin/pretrichodermamide for 86, 40, and 33 proteins, respectively, with phenylalanine as the substrate specificity for all but four of these proteins. However, errors are possible in the predictions by antiSMASH since two of the BGCs were predicted to synthesize clapurines and sporidesmin, whereas these metabolites result from the condensation of two distinct amino acids.

**Table 1:** The “aspirochlorine” biosynthetic gene cluster in *S. apiospermum* IHEM 14462 as revealed by antiSMASH analysis and its orthologs in the *Scedosporium* genus

***Scedosporium* species and strain**	Contig or scaffold	Cluster position	Genes detected	NRPS domain architecture	PKS domain architecture	Amino acid predicted	SM predicted
***S. apiospermum* IHEM 14462**	Contig 77 (JOWA01000077.1)	623,914–672,358	9	C1-A-PP-C2	KS-AT-DH-cMT-ER-KR-PP	Phenylalanine	Aspirochlorine
*S. apiospermum* HDO1	Scaffold 7 (MVOQ01000007.1)	740,213–798,194	9*	C1-A-PP-C2	KS-AT-DH-cMT-ER	Phenylalanine	Acetylaranotin
*S. apiospermum* 2RF1-5	Scaffold 181 (JASXSD010000181.1)	597–56,371 (scaffold edge)	9*	C1-A-PP-C2	KS-AT-DH-cMT-ER-KR-PP	Phenylalanine	Acetylaranotin
*S. aurantiacum* MUT 6114	Scaffold 46 (SJVH01000046.1)	332–61,732 (scaffold edge)	8	PP-C	KS-AT-DH-cMT-ER-KR-PP	No prediction	No prediction
***S. aurantiacum* WM 09.24**	Scaffold 93 (JUDQ01000093.1)	31,217–66,003	7	PP-C	Not detected	No prediction	Aspirochlorine
***S. boydii* IHEM 23826**	Contig 58 (NJFT01000058.1)	101,768–167,415	8	C1-PP-C2	KS-AT-DH-ER-PP	No prediction	Aspirochlorine
***S. dehoogii* UA120008799-01/4**	Contig 17 (PGIR01000017.1)	3,289–64,389 (contig edge)	11	C1-PP-C2	KS-AT-DH-cMT-ER-KR-PP	No prediction	Acetylaranotin
*S. minutisporum* MUT 6113	Contig 98 (SJVG01000098.1)	34,961–57,820 (contig edge)	6	C1-A-PP-C2	Not detected	No prediction	Acetylaranotin

Reference genomes are indicated in bold font

^*^The genes *SAPIO_CDS1831* and *SAPIO_CDS1832* in *S. apiospermum* reference genome are fused in the genomes of these strains and the SAM-dependent methyl transferase and glutathione-S-transferase activities are supported by the same protein

A: Adenylation domain; AT: acyl transferase domain; C1, C2: condensation domains; cMT: carboxymethyl transferase domain; DH: dehydratase domain; ER: enoyl reductase domain; KR: ketoreductase domain; KS: ketosynthase domain; NRPS: non-ribosomal peptide synthase; PKS: polyketide synthase; PP: phosphopantetheine (for thiolation domain); SM: secondary metabolite

**Fig. 1:**
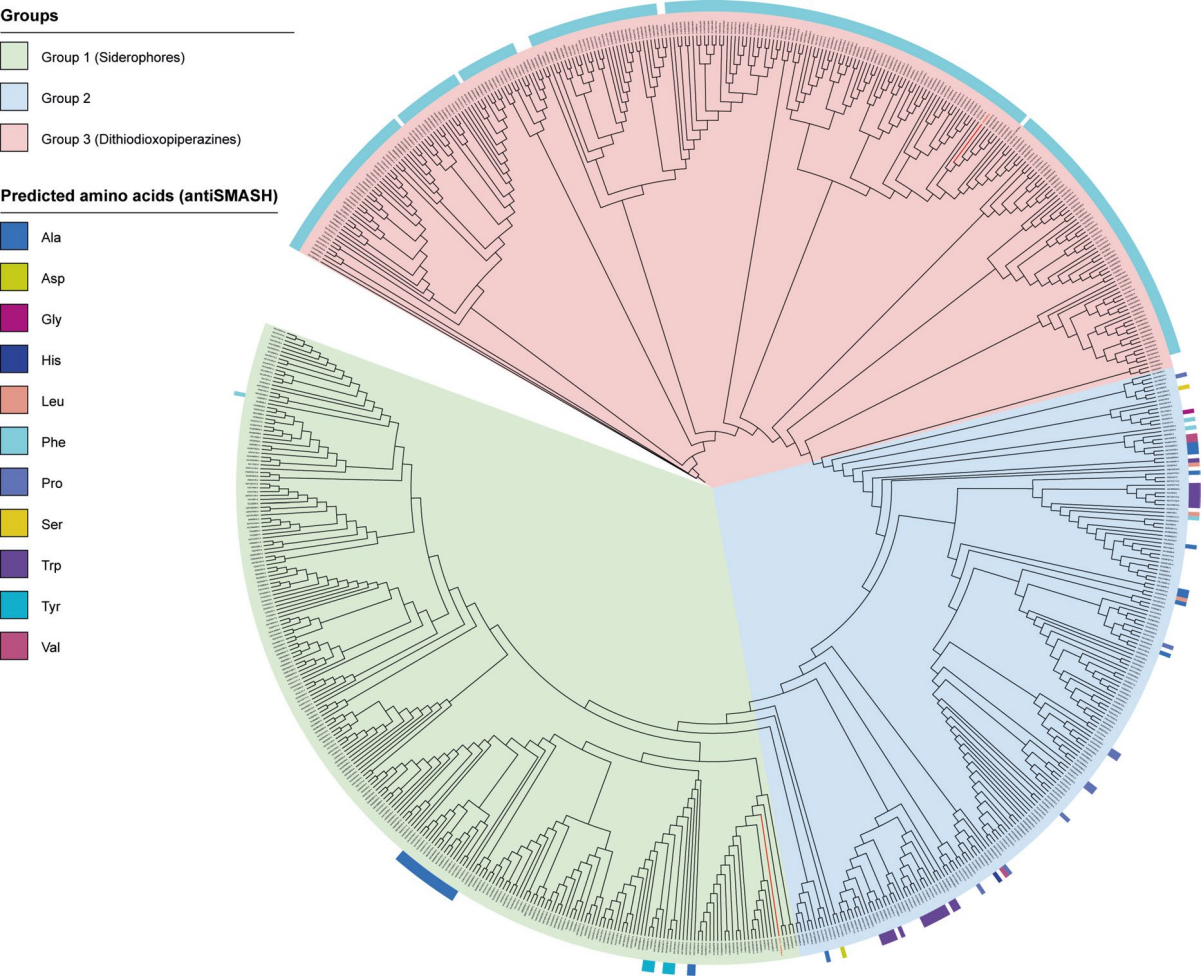
Phylogenetic tree of NRPSs exhibiting a single adenylation domain and two condensation domains, and therefore possibly involved in the synthesis of homodipeptides. NRPSs were identified by antiSMASH analysis of available fungal genomes presenting potential orthologs of *SAPIO_CDS1828*, or of the NRPSs AtaP (encoded by *ATEG_03470*) or AclP (encoded by *AO090001000043*), which are responsible for the synthesis of acetylaranotin and aspirochlorine in *A. terreus* strain NIH 2624 and *A. oryzae* strain RIB 40, respectively (Supplementary Tables S1 and S2). Phylogenetic analysis was performed using IQ-TREE software version 3.0. Three groups were identified: (i) Group 1 comprising 248 proteins mainly corresponding to NRPSs allowing the synthesis of siderophores; (ii) Group 2 comprising 193 proteins for which no prediction could be made regarding the produced metabolite or the recognized amino acid; and (iii) Group 3 comprising 278 proteins, corresponding mainly to NRPSs allowing the synthesis of cyclo-(Phe-Phe). *Scedosporium apiospermum* SidDp, encoded by *SAPIO_CDS2806*, belonged to Group 1, and KEZ45505, encoded by *SAPIO_CDS1828*, belonged to Group 3; these two proteins are highlighted in red bold font

A phylogenetic analysis of these NRPSs, based on the amino acid sequence of their adenylation domain, revealed three distinct groups of proteins (Fig. 1). The first group, which comprised 248 proteins, including SidDp of *S. apiospermum* (encoded by *SAPIO_CDS2806*), corresponded mainly to proteins predicted to be involved in the synthesis of siderophores (metachelin-type siderophores or dimethylcoprogen for 131 and 39 of these proteins, respectively; no prediction for almost all other proteins). These siderophores result from the assembly of three identical building blocks (which explains the presence of a single adenylation domain in these NRPSs), consisting of anhydromevalonate (a derivative of the ergosterol biosynthesis pathway) combined with N5-hydroxyornithine (a degradation product of arginine). Whereas antiSMASH failed to predict in most cases the nature of the amino acid recognized by these NRPSs (223/248) or predicted alanine (19/248), PARAS v1.0.1 indicated N5-*cis*-anhydromevalonyl-N5-hydroxyornithine as the substrate specificity for the adenylation domain of 163 out of the 248 proteins.

For the second group composed of 193 proteins, in most cases, antiSMASH did not provide any prediction regarding the metabolite produced by the BGC (162/193) nor the amino acid recognized (140/193). A large variety of secondary metabolites was predicted to be synthesized by the other BGCs, but these predictions often were erroneous or not related to the studied NRPS. This is likely due to the presence of the encoding gene in a genomic region also comprising another core gene responsible for the synthesis of the predicted metabolite. For example, dehydrocurvularin and squalestatin are produced through the PKS pathway. Likewise, the synthesis of chaetolivacine or oxaleimide is initiated by hybrid enzymes called PKS-NRPSs. However, the second group comprised a subgroup of 9 proteins (recognizing tryptophan for 7 and 8 of them according to antiSMASH and PARAS) that were predicted by antiSMASH to ensure the synthesis of the non-sulfur diketopiperazines (DKPs) notoamides (OJJ76279, RAK93182, BCR96254, PYH36058, PYH62947, RDK41754, RAH59955, GFN16130, and PYH74752), which result from the assembly of two tryptophan molecules.

*Scedosporium apiospermum* NRPS KEZ45505 encoded by *SAPIO_CDS1828* belonged to the third group, which comprised 278 NRPSs. Almost all of these proteins were predicted to recognize phenylalanine (by both antiSMASH and PARAS v1.0.1 algorithms for 265 of them, and by PARAS only for 10 others) and, therefore, to be involved in the synthesis of cyclo-(Phe-Phe) derivatives (*i.e.* aspirochlorine, acetylaranotin, and penigainamide / outovirin / pretrichodermamide for 86, 40 and 33 of these proteins).

### The “Aspirochlorine” Biosynthetic Gene Cluster Comprises a Total Number of 15 Genes

According to the antiSMASH analysis, *SAPIO_CDS1828* belonged to a 9-membered BGC located on contig 77 (nucleotides 623,914 to 672,358), and predicted to ensure the synthesis of aspirochlorine (Table 1). Table 2 summarizes the InterProscan annotation, domains, motifs, and predicted functions of the proteins encoded by the different genes of this BGC.

**Table 2:** Domains and function of the proteins encoded by members of the “aspirochlorine” biosynthetic gene cluster in *S. apiospermum* IHEM 14462

Gene	Position	Length (bp)	Introns	GenBank accession number of the protein	Size of the protein (AA)	InterProScan annotation	Domains and motifs	Predicted function
*SAPIO_CDS1819*	623,914 – 632,483	8,570	12	KEZ45498	2,490	Polyketide and nonribosomal peptide biosynthesis enzyme	Beta-ketoacyl synthase (KS3_2) Acyl transferase (PKS_AT) Dehydratase (PKS/mFAS DH) Carboxy- methyltransferase (cMT) Enoyl reductase (PKS_ER) Keto reductase (PKS_KR) Acyl carrier protein (PP)	PKS (synthesis of the alkyl chain of boydines B, C and D)
*SAPIO_CDS1820*	633,038 – 633,952	915	0	–	304	No annotation	Domain of unknown function (DUF7136)	/
*SAPIO_CDS1821*	637,360 – 636,323	1,038	1	KEZ45499	322	No annotation	3 extracellular domains 5 transmembrane domains 3 cytoplasmic domains	Membrane bound O-acyl transferase
*SAPIO_CDS1822*	638,153 – 639,832	1,680	2	KEZ45500	509	Cytochrome P450 monooxygenase (E class, group I)	1 extracellular domain 1 transmembrane domain 1 cytoplasmic domain	Cytochrome P450 monooxygenase (hydroxylation of Cyclo-Phe-Phe)
SAPIO_CDS1823*	641,272 – 639,955	1,318	3	KEZ45501	262	No annotation	AdoMet_MTase (Methyltransferase-23; pfam13489)	SAM-dependent methyltransferase
*SAPIO_CDS1824*	643,799 – 642,436	1,364	2	KEZ45502	391	No annotation	1 Gal4 domain	Putative transcription factor
*SAPIO_CDS1825*	647,607 – 645,808	1,800	3	KEZ45503	515	Cytochrome P450 monooxygenase (E class, group IV)	2 extracellular domains 3 transmembrane domains 2 cytoplasmic domains	CYP503A1 (AtaF)
*SAPIO_CDS1826*	647,954 – 648,873	920	1	KEZ45504	277	GGCT	GGCT-like	Gamma-glutamyl cyclotransferase (degradation of glutathione)
*SAPIO_CDS1827*	650,977 – 649,237	1,741	0	/	562	MEROPS peptidase family M19 (membrane dipeptidase family, clan MJ)	Non-cytoplasmic domain of a membrane-embedded protein	Dipeptidase (degradation of glutathione)
*SAPIO_CDS1828*	651,701 – 656,629	4,929	2	KEZ45505	1,597	No annotation	NRPS (1 adenylation domain, 2 condensation domains, 2 acyl-carrier protein domains)	NRPS (synthesis of Cyclo-[Phe-Phe])
*SAPIO_CDS1829*	659,514 – 661,120	1,607	3	KEZ45506	442	Ethylene and sulfur compound biosynthesis-related protein	Aspartate aminotransferase – like Three 1-aminocyclopropane1 carboxylate synthase signature motifs	Aspartate aminotransferase (degradation of glutathione)
*SAPIO_CDS1830*	662,826 – 661,778	1,049	1	KEZ45507	322	Ferredoxin–NADP reductase type 2	Pyridine nucleotide-disulphide oxidoreductase 3 FAD-dependent pyridine nucleotide reductase signature motifs (9–28, 107–125, 269–291) 4 pyridine nucleotide-disulphide reductase class-II signature motifs (8–30, 108–116, 130–142, 279–297)	Thioredoxine reductase
*SAPIO_CDS1831*	663,011 – 664,835	1,825	2	KEZ45508	531	O-methyltransferase	SAM-dependent O-methyltransferase class II (Methyltransferase-2; pfam PF00891)	SAM-dependent O-methyltransferase
*SAPIO_CDS1832*	665,054 – 666,064	1,011	3	KEZ45509	225	Glutathione transferase family	Glutathione S-transferase	Glutathione S-transferase
*SAPIO_CDS1833*	667,895 – 669,370	1,476	0	/	491	MFS	6 extracellular domains 11 transmembrane domains 6 cytoplasmic domains 9 helices	MFS (efflux of metabolites)

^*^SAPIO_CDS1823 had no orthologs in *A. terreus* genome nor in the Aspirochlorine BGC in *A. oryzae*

AA: amino acid; AT: acyl transferase domain; cMT: carboxymethyl transferase domain; DH: dehydratase domain; ER: enoyl reductase domain; GGCT: gamma-glutamyl cyclotransferase; KR: ketoreductase domain; KS: ketosynthase domain; MFS: major facilitator superfamily; NRPS: non-ribosomal peptide synthase; PKS: polyketide synthase; PP: phosphopantetheine (for thiolation domain); SAM: S-adenosylmethionine

In the environment of *SAPIO_CDS1828,* antiSMASH analysis identified: (i) an *AtaTC-*3’ end and *AclC* ortholog, *SAPIO_CDS1822,* encoding a P450 cytochrome that allows the hydroxylation of the DKP ring; (ii) an *AtaIMG*-3′ end and *AclG* ortholog, *SAPIO_CDS1832*, encoding the glutathione S-transferase which allows condensation of the tripeptide glutathione with the hydroxylated DKP; and (iii) an *AtaIMG-*5’ end and *AclI* ortholog, *SAPIO_CDS1829,* encoding an aspartate amino transferase which allows the final step in the hydrolysis of bound glutathione. Of note, the *AclK* ortholog *SAPIO_CDS1826* and the *AtaJ*/*AclJ* ortholog *SAPIO_CDS1827*, encoding respectively a gamma-glutamyl transferase and a membrane dipeptidase, which catalyze the two initial steps in the degradation of glutathione, were identified by BLAST searches.

Four other genes were detected in the *S. apiospermum* “aspirochlorine” BGC, including the *AtaTC*-5′ end/*AclD* ortholog (*SAPIO_CDS1830*), the *AtaF*/*AclB* ortholog (*SAPIO_CDS1825*), and the *AtaIMG*-center/*AclM* ortholog (*SAPIO_CDS1831*) encoding a TrxR, another cytochrome, and a S-adenosyl methionine (SAM)-dependent methyltransferase, respectively, as well as the gene *SAPIO_CDS1823* also encoding a methyltransferase, which had no ortholog in *A. terreus* acetylaranotin BGC nor in *A. oryzae* aspirochlorine BGC. Conversely to its counterpart in *A. terreus* and *A. oryzae*, the *S. apiospermum* “aspirochlorine” BGC also comprised a gene encoding a PKS (*SAPIO_CDS1819*), with the KS-AT-DH-cMT-ER-KR-PP domain architecture.

**Fig. 2:**
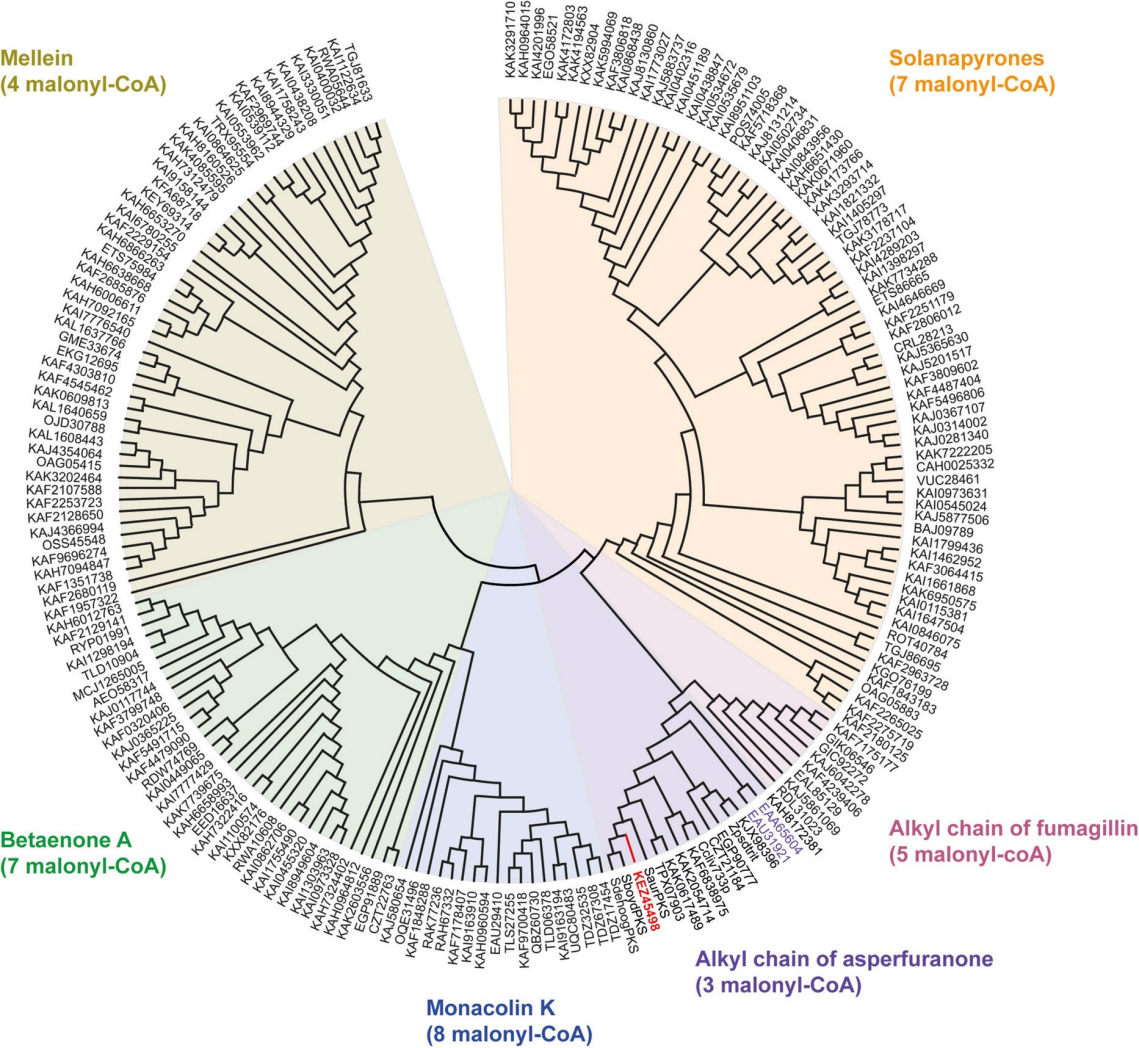
Phylogenetic analysis of selected PKSs allowing the assembly of well-defined numbers of condensation units. A database encompassing 201 PKSs was built (Supplementary Table S3), and a maximum likelihood phylogenetic tree was constructed using MEGA XI based on the amino acid sequences of the acyl carrier domain (PP domain) of these proteins. The *S. apiospermum* PKS KEZ45498 (indicated in red bold font) and its closest orthologs clustered with PKSs allowing the synthesis of the alkyl chain of asperfuranone, thus enabling the assembly of three condensation units

To determine the number of condensation units assembled by this PKS, the peptide sequence of the thiolation domain was compared to that of the PP domain of fungal PKSs governing the synthesis of well-known polyketides, the elongation of the alkyl chain taking place in this domain. A database of 201 amino acid sequences was analyzed with MEGA XI software (Supplementary Table S3). All these peptides presented the GxDS signature sequence, which corresponds to the phosphopantheteine attachment site. Moreover, the phylogenetic analysis depicted in Fig. 2 revealed four distinct clades: (i) a first clade consisting of PKSs ensuring the synthesis of mellein (4 condensation units); (ii) a second clade splitting in two subgroups, with PKSs enabling the synthesis of betaenone A (7 units) or monacolin K (8 units); (iii) a third clade also dividing into two subgroups, with PKSs involved in the synthesis of the alkyl chain of asperfuranone (3 units), or of fumagillin (5 units); and finally *iv*), a large clade of PKSs enabling the synthesis of solanapyrones (7 units). KEZ45498 clustered with EAA65604 and EAU31921, which ensure the synthesis of the alkyl chain of asperfuranone in *Aspergillus nidulans* and *A. terreus*, respectively. Therefore, this suggested that KEZ45498 enables the assembly of three malonyl-CoA units for chain elongation, which is consistent with the length of the alkyl chain of boydines, the [2*R*,3*S*,4*S,E*]-3-hydroxy-2,4,6-trimethyl-5-oxooct-6-enoic acid, taking into account the initiation of its synthesis with one acetyl-CoA starter unit. Interestingly, two of the carbonyl groups on the alkyl chain undergo reduction, and one of the resulting hydroxyl groups is removed. This process requires the presence of KR and DH domains in the PKS. In addition, three methyl groups are branched on the polyketide chain in boydines, which implies the presence of a cMT domain in the PKS. All these optional domains were detected in KEZ45498, together with an ER domain which does not seem to be functional, as the sole C–C double bond generated by the KR and DH domains is maintained in the alkyl chain of boydines.

**Fig. 3:**
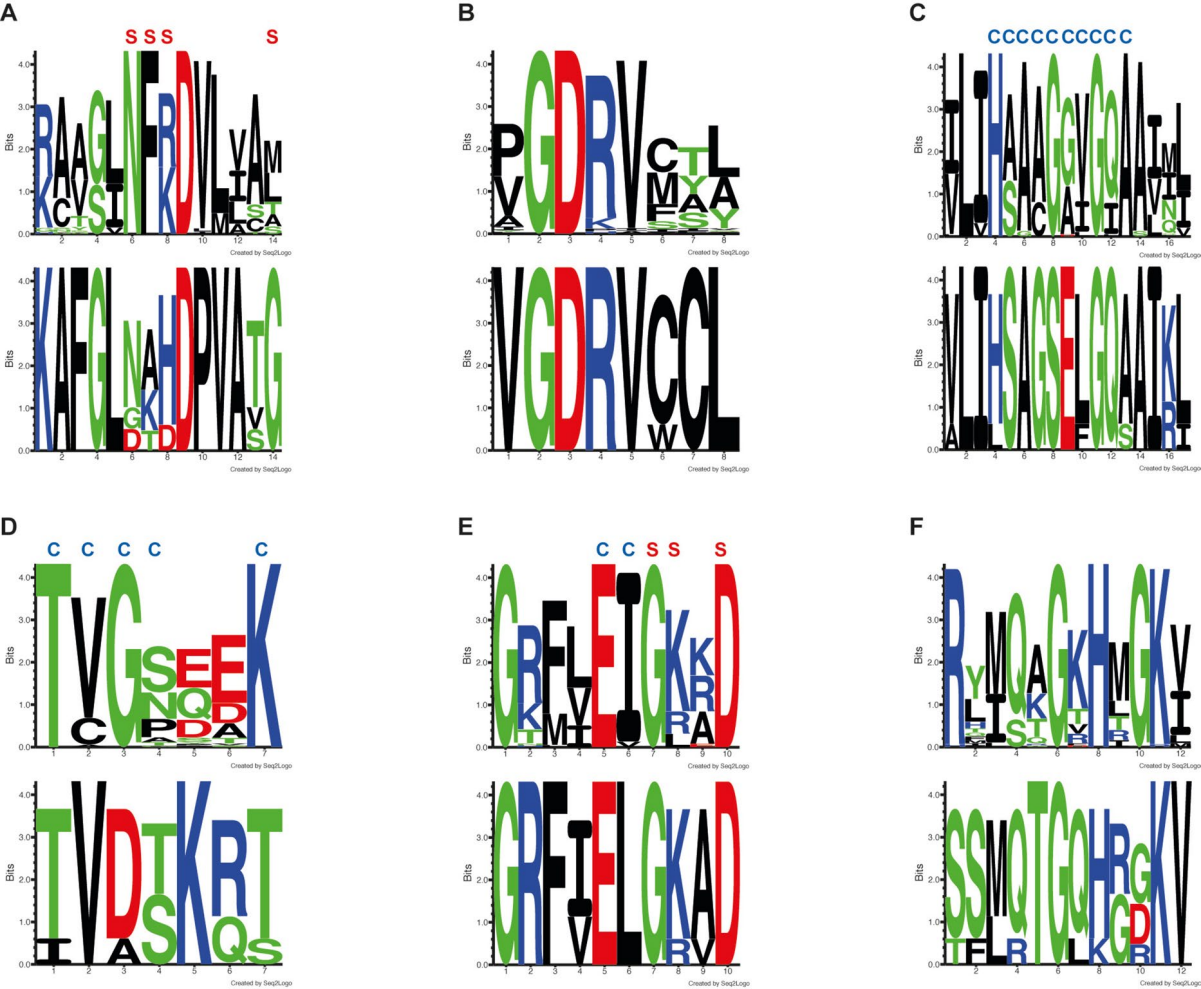
Signature logos in the enoyl reductase domain of iterative polyketide synthases. PKSs exhibiting a functional enoyl reductase (ER) domain were compared with *S. apiospermum* PKS KEZ45498 and its orthologs in other *Scedosporium* species and strains (Supplementary Table S4). In each panel of the figure, the signature logo of the ER domain of the reducing PKSs is presented on top, and that of KEZ45498 and its orthologs on the bottom. Two conserved sequences were found that may be the signature sequences of ER domains in iterative PKSs (**B** and **F**), which could explain the detection of this domain by antiSMASH and InterProScan in KEZ45498. However, some of the amino acids involved in the recognition of the substrate (**S**) or of the NADP(H) + cofactor (**C**) were mutated in KEZ45498 (**A**, **C**, **D**, and **E**)

To confirm this hypothesis, the peptide sequence of the ER domain of KEZ45498 was compared to those of the ER domain of reducing iterative PKSs involved in the synthesis of asperfuranone, fumonisin, zearalenone, or alternapyrone, or of some cyclodepsipeptides comprising a fully saturated alkyl chain (BII-rafflesfungin, emericellamide, W 493 A/W 493 B or beauveriolides). Sixty-one amino acid sequences from PKSs with a functional ER domain were compiled and analyzed using MEGA XI (Supplementary Table S4). The analysis revealed a high degree of sequence conservation, with 28 identical and 30 similar amino acids among a total number of 308 to 312 amino acids. In particular, two conserved sequences were found in all ER domains, likely the signature sequences of the ER domains of iterative PKSs (Fig. 3B and F). However, some of the amino acids involved in the recognition of the substrate or of the NADP(H) + cofactor were found mutated in KEZ45498 (Fig. 3A, C, D, and E). For example, in the sequence NF(R/K)D, whose first three amino acids are involved in substrate recognition [31], a threonyl residue was substituted to the phenylalanyl residue in KEZ45498 (Fig. 3A). Likewise, in the sequence Hx_3_Gx_2_GxA, which is usually present in functional ER domains and is involved in the recognition of NADP(H) + [32], the first glycyl residue was replaced with a seryl residue (Fig. 3C). Similarly, in the sequence TxGx_3_K, which is also frequently observed in functional ER domains and is involved in cofactor binding, the glycyl and lysyl residues were replaced with an aspartic acid and a threonyl residue, respectively, in KEZ45498 (Fig. 3D). Taken together, these mutations could explain why the ER domain in KEZ45498 is not functional.

The *S. apiospermum* “aspirohlorine” BGC also comprised an ortholog of *AtaH* (*SAPIO_CDS1821*) encoding an acetyl transferase that allows in *A. terreus* the conversion of aranotin in acetylaranotin, and an ortholog (*SAPIO_CDS1833*) of *ATEG_03467*, encoding an efflux protein belonging to the Major Facilitator Superfamily (MFS) and allowing the active efflux of the synthesized metabolites. During annotation of *S. apiospermum* reference genome, *SAPIO_CDS1833* was considered as a pseudogene due to the lack of an intron, and therefore, was not detected by antiSMASH analysis but only by BLAST searches. Analysis of the protein sequence deduced from *SAPIO_CDS1833* using the PSI-Blast algorithm allowed us to classify this protein among the 2.A.1 superfamily of membrane transporters (*i.e.* MFSs), more precisely in family 2.A.1.16, which corresponds to the Sit siderophore transporters. InterProScan also identified this protein as a membrane transporter, with ferrisiderophore transporter-like activity, but only eleven transmembrane domains were detected. Nevertheless, analysis of this sequence with Protscale and the Transmembrane tendency algorithm, revealed the expected presence of twelve transmembrane domains, which was confirmed using the HMMTOP, CCTOP, and WHAT algorithms.

Finally, this BGC also comprised a transcription factor-encoding gene, *SAPIO_CDS1824*. However, the protein sequence deposited in the GenBank Database (KEZ45502) revealed only three cysteinyl residues with a spacing suggestive of a Gal4-type transcription factor (Cx_2_Cx_6_C motif). Nevertheless, a BLAST search for sequence homology revealed high homology with typical fungal Gal4-type Zn2Cys6 transcription factors. Strikingly, two introns of 66 and 122 bp were reported in the GenBank database for *SAPIO_CDS1824,* separated by a very short exon of only 13 bp. As expected, analysis of the deduced amino acid sequence with PANNZER2 suggested a Zn2Cys6 transcription factor. Likewise, analysis of the nucleotide sequence with GENSCAN revealed a single intron within *SAPIO_CDS1824*, and six cysteinyl residues with the typical Cx_2_Cx_6_Cx_6_Cx_2_Cx_6_C spacing pattern of Gal-type transcription factors were predicted in the deduced protein*.*

To sum up, the *S. apiospermum* “aspirochlorine” BGC comprises 15 genes instead of the 9 genes predicted by antiSMASH analysis. The NRPS-encoding gene *SAPIO_CDS1828* stands out as the core gene of this BGC, along with 10 decorating genes, including a single TrxR-encoding gene, two genes encoding cytochromes and one encoding a PKS, and with two genes encoding a Gal4-type transcription factor and an efflux protein (Table 2). Of note, the function of *SAPIO_CDS1820* remains elusive, as well as that of *SAPIO_CDS1823* which encodes a putative methyltransferase.

### The “Aspirochlorine” Biosynthetic Gene Cluster is Responsible for the Synthesis of Boydines

The synthesis of aspirochlorine requires two TrxRs, the first one forming the disulfide bond, while the second, which acts after hydroxylation of the ETP through the cytochrome AclB, ensures both the migration of one sulfur atom and spiroformation. In addition, two other cytochromes are also needed (encoded by *AclL* and *AclO*), the second being responsible together with an O-methyl transferase, encoded by *AclU*, for the degradation of one of the phenylalanyl residue and its conversion to a glycyl residue. Finally, the halogenase encoded by *AclH* allows chloration of the remaining aromatic ring. None of these genes had an ortholog in the *S. apiospermum* “aspirochlorine” BGC. Only one gene encoding a TrxR, with the canonical peptide motif CxxC, was found in *S. apiospermum* “aspirochlorine” BGC; likewise, this cluster comprised only two cytochrome-encoding genes, *SAPIO_CDS1822 and SAPIO_CDS1825,* the respective orthologs of *AtaTC* / *AclC* and *AtaF* / *AclB*.

**Fig. 4:**
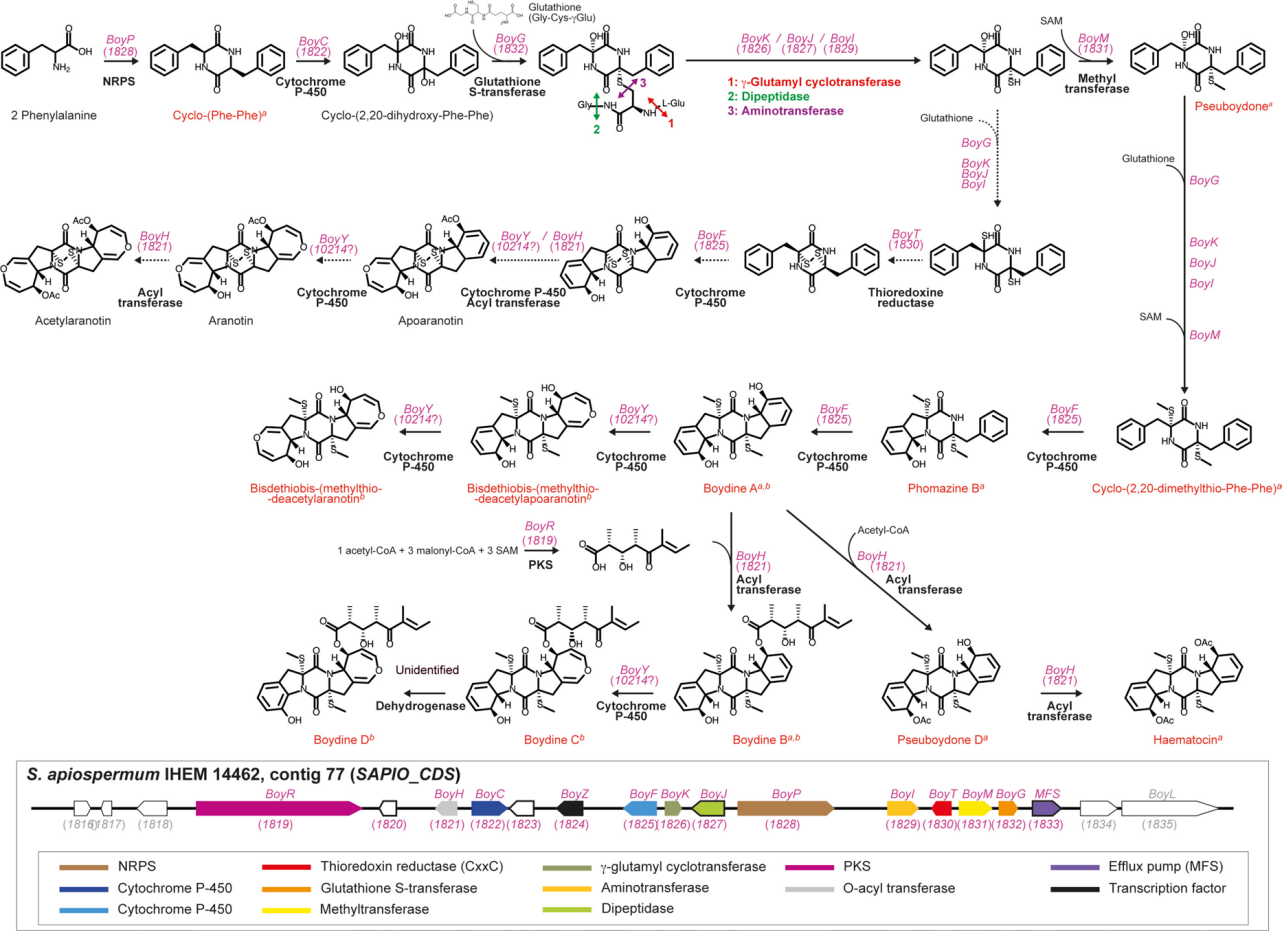
The boydines biosynthetic gene cluster (BGC) in *Scedosporium apiospermum* IHEM 14462 and a proposed metabolic pathway. A metabolic pathway is proposed starting with the assembly of two phenylalanine molecules. Metabolites and biosynthesis intermediates already identified in *Scedosporium* species are indicated in red (^*a*^, identified by Lan et al., 2016; ^*b*^, identified by Wu et al., 2014). GenBank accession numbers of the genes are indicated in parentheses (in pink for members of the BGC that are named *Boy* for boydines biosynthesis, in grey for non-members). Genes outlined in black were erroneously considered as pseudogenes during the annotation of the reference genome using Augustus. PKS: polyketide synthase; MFS: major facilitator superfamily; NRPS: non-ribosomal peptide synthase; SAM: S-adenosylmethionine

Together, these data provide firm evidence that the *S. apiospermum* BGC centered on *SAPIO_CDS1828* allows the synthesis of the aratonin-related compounds boydines. Figure 4 depicts the BGC and a proposed metabolic pathway for boydines biosynthesis. The biosynthetic pathways for acetylaranotin synthesis in *A. terreus* and aspirochlorine synthesis in *A. oryzae*, together with their respective BGCs, are presented in Supplementary Figure S1 for comparison. In *S. apiospermum*, three distinct proteins, BoyG, BoyI and BoyM, support glutathione S-transferase, aspartate aminotransferase and SAM-dependent methyl transferase activities ensured in *A. terreus* by the same protein, AtaIMG. Moreover, an additional gene, *BoyR*, was identified, which encodes a PKS allowing the synthesis of an unsaturated and branched alkyl chain, and the conversion of boydine A to boydine B. Nevertheless, BLAST searches failed to identify the ortholog of *AtaY* in the boydines BGC, and suggested that the conversion of aromatic rings to oxepine rings is ensured by the product of *SAPIO_CDS10214* which is located on contig 165.

### The Boydines BGC is Shared by all *S. apiospermum* Strains and *Scedosporium* Species

**Fig. 5:**
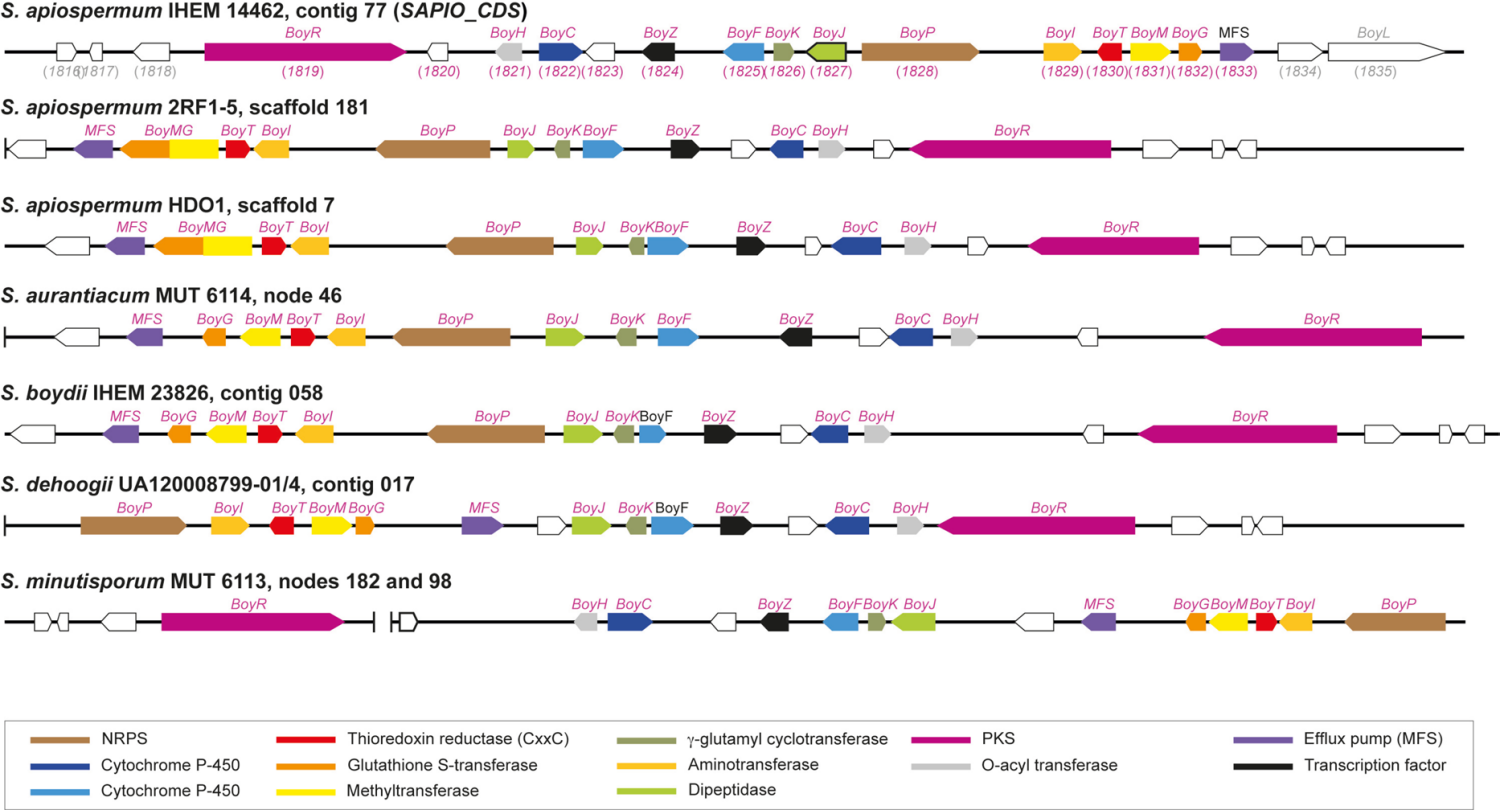
The boydines biosynthetic gene cluster in other* Scedosporium apiospermum* strains and non*-apiospermum Scedosporium* species*.* The boydines BGC identified in *S. apiospermum* strain IHEM 14462 (originally recovered from respiratory secretions of a French CF patient) was also detected in the genome of two other *S. apiospermum* strains (the endophytic strain 2RF1-5 collected from roots of *Hypericum maculatum* in Slovakia, and the strain HDO1 recovered from soil in Columbia), as well as in all other *Scedosporium* genomes available in the GenBank database. A unique TrxR-encoding gene was found in all species and strains, and the corresponding proteins always exhibited the canonical peptide sequence CxxC. However, compared to *S. apiospermum* IHEM 14462, the SAM-dependent methyltransferase and glutathione S-transferase activities were supported by the same protein, BoyMG, in the two other *S. apiospermum* strains; in addition, whereas the synteny was rigorously conserved in the genome of all *S. apiospermum* strains, as well as in *S. aurantiacum* and *S. boydii*, a chromosome rearrangement was seen for *S. dehoogii* and *S. minutisporum*. GenBank accession numbers of the genes are indicated in parentheses (in pink for members of the BGC that are named *Boy* for boydines biosynthesis, in grey for non-members) for *S. apiospermum* IHEM 14462 (genome not annotated for the other *S. apiospermum* strains, nor the other *Scedosporium* species). PKS: polyketide synthase; MFS: major facilitator superfamily; NRPS: non-ribosomal peptide synthase

Since the availability of the reference genome (from the clinical strain IHEM 14462), four other *S. apiospermum* genomes have been deposited in the GenBank database. The boydines BGC was detected in the four genomes, notably in the 2RF1-5 and HDO1 strains (Fig. 5 and Table 1). For the two other strains, orthologs were found for all members of the cluster, but a BGC could not be identified because these genomes were highly fragmented, each contig corresponding to a single gene, or even part of a gene. For example, the ortholog of *SAPIO_CDS1835* was distributed over two contigs in the B4 and B8 strains. Of note, the two genes *SAPIO_CDS1831* and *SAPIO_CDS1832* were fused in the HDO1 and 2RF1-5 genomes, and antiSMASH predicted acetylaranotin synthesis for the corresponding BGCs.

This BGC was also detected in all other *Scedosporium* genomes available in the GenBank database. antiSMASH predicted aspirochlorine synthesis by this BGC in *S. aurantiacum* strain MUT6114 and *S. boydii* strain IHEM 23826*,* but acetylaranotin synthesis in *S. dehoogii* strain UA120008799-01/4 and *S. minutisporum* strain MUT 6113 (Fig. 5 and Table 1). For the latter, the PKS-encoding gene was located at the 3’-end of node 182, while the other genes were located at the 5’-end of node 98. However, PCR using primers targeting the ends of these contigs, followed by sequencing of the amplified product, confirmed the joining of the two contigs (Supplementary Figure S2). In addition, whereas the synteny was rigorously conserved in the genomes of *S. aurantiacum* and *S. boydii*, a chromosomal rearrangement affecting the boydines BGC was observed in *S. dehoogii* and *S. minutisporum*. This was confirmed by PCR and sequencing with primers targeting the end of the MFS-encoding gene and *BoyJ* (Supplementary Figures S2 and S3). Conversely but interestingly, all our attempts to identify a similar BGC in the genome of the former *Scedosporium prolificans*, now called *Lomentospora prolificans*, were unsuccessful.

### The Boydines BGC is Not Specific to the *Scedosporium* Genus

As shown in Fig. 1, the closest proteins to the NRPS KEZ45505 were produced by other Sordariomycetes belonging to the orders Glomerellales (*Colletotrichum caudatum* and *Colletotrichum musicola*), Hypocreales (*Sarocladium implicatum*), Magnaporthales (*Pyricularia oryzae*), and Sordariales (*Immersiella* c*audata*), as well as unclassified Sordariomycetes, such as Thyridiaceae (*Thyridium curvatum*). Interestingly, the more phylogenetically distant filamentous fungus, *Ramularia collo-cygni*, also produce these proteins. In addition, two other fungal species belonging to the Mycosphaerellaceae family, *Zymoseptoria brevis* and *Zymoseptoria tritici*, exhibited a BGC associating genes encoding a Group 3-NRPS and a PKS with a domain architecture similar to that described for KEZ 45498*.* For all these fungi, the corresponding BGCs were investigated, and for BGCs located at contig edge, we looked for orthologs of the lacking genes by BLAST searches.

**Fig. 6:**
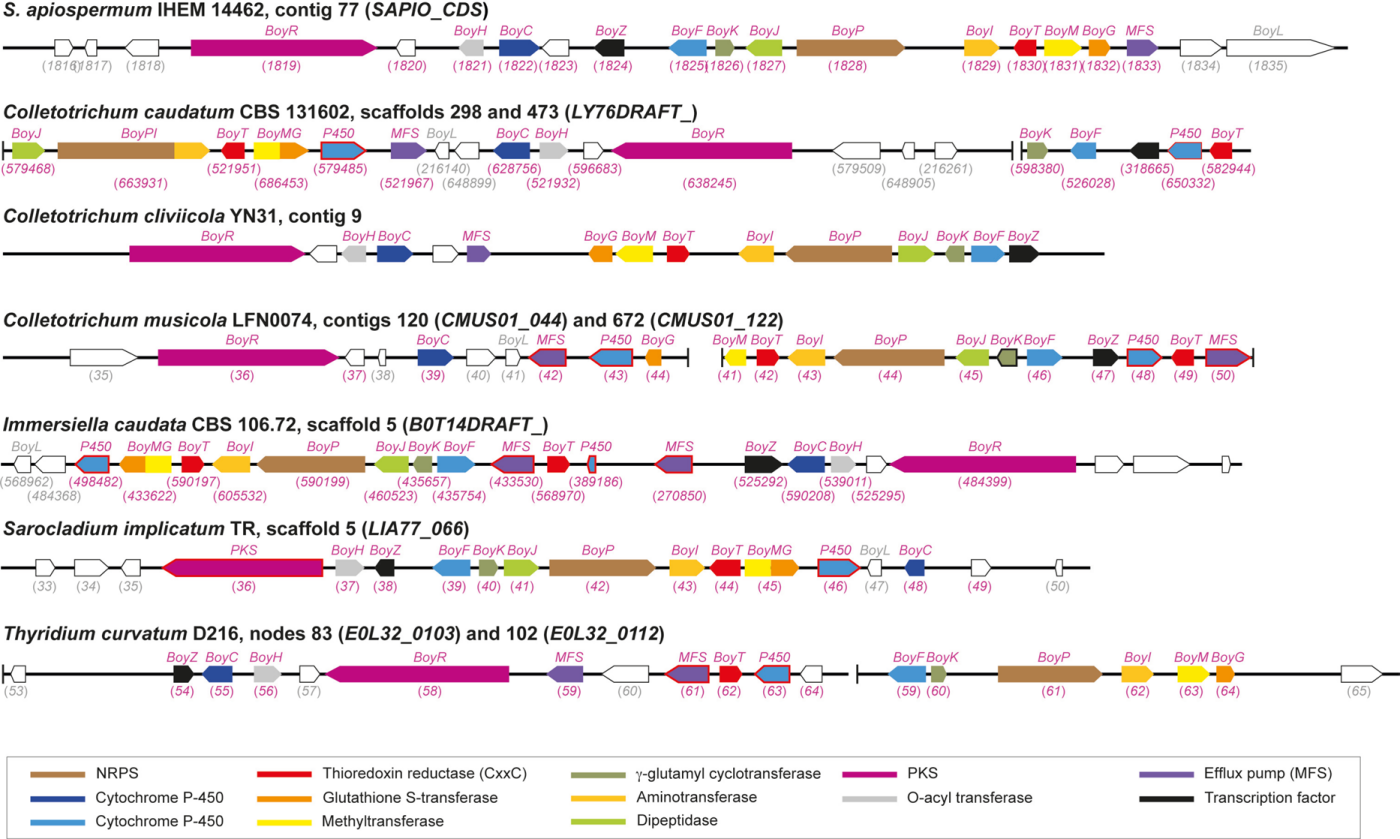
The boydines biosynthetic gene cluster in non-*Scedosporium* Sordariomycetes. Almost all the genes of the *S. apiospermum* boydines BGC were found in the genome of other Sordariomycetes, including some *Colletotrichum* species (*i.e. C. caudatum, C. cliviicola* and* C. musicola*),* Immersiella caudata, Sarocladium implicatum* and* Thyridium curvatum.* Compared to *S. apiospermum* IHEM14462, the SAM-dependent methyltransferase and glutathione S-transferase activities were supported by the same protein, BoyMG, for *C. caudatum* and *I. caudata*. Likewise, the gene cluster comprised two TrxR-encoding genes in *C. caudatum*, *C. musicola*, and *I. caudata*, but all the corresponding proteins exhibited the canonical peptide sequence CxxC. GenBank accession numbers of the genes are indicated in parentheses (in pink for members of the BGC that are named *Boy* for boydines biosynthesis, in grey for non-members), except for *C. cliviivola* since its genome is not annotated. Genes without ortholog in *S. apiospermum* boydines BGC are outlined in red, and gene encoding BoyK in *C. musicola* (outlined in black) was detected only by BLAST searches. PKS: polyketide synthase; MFS: major facilitator superfamily; NRPS: non-ribosomal peptide synthase

**Fig. 7:**
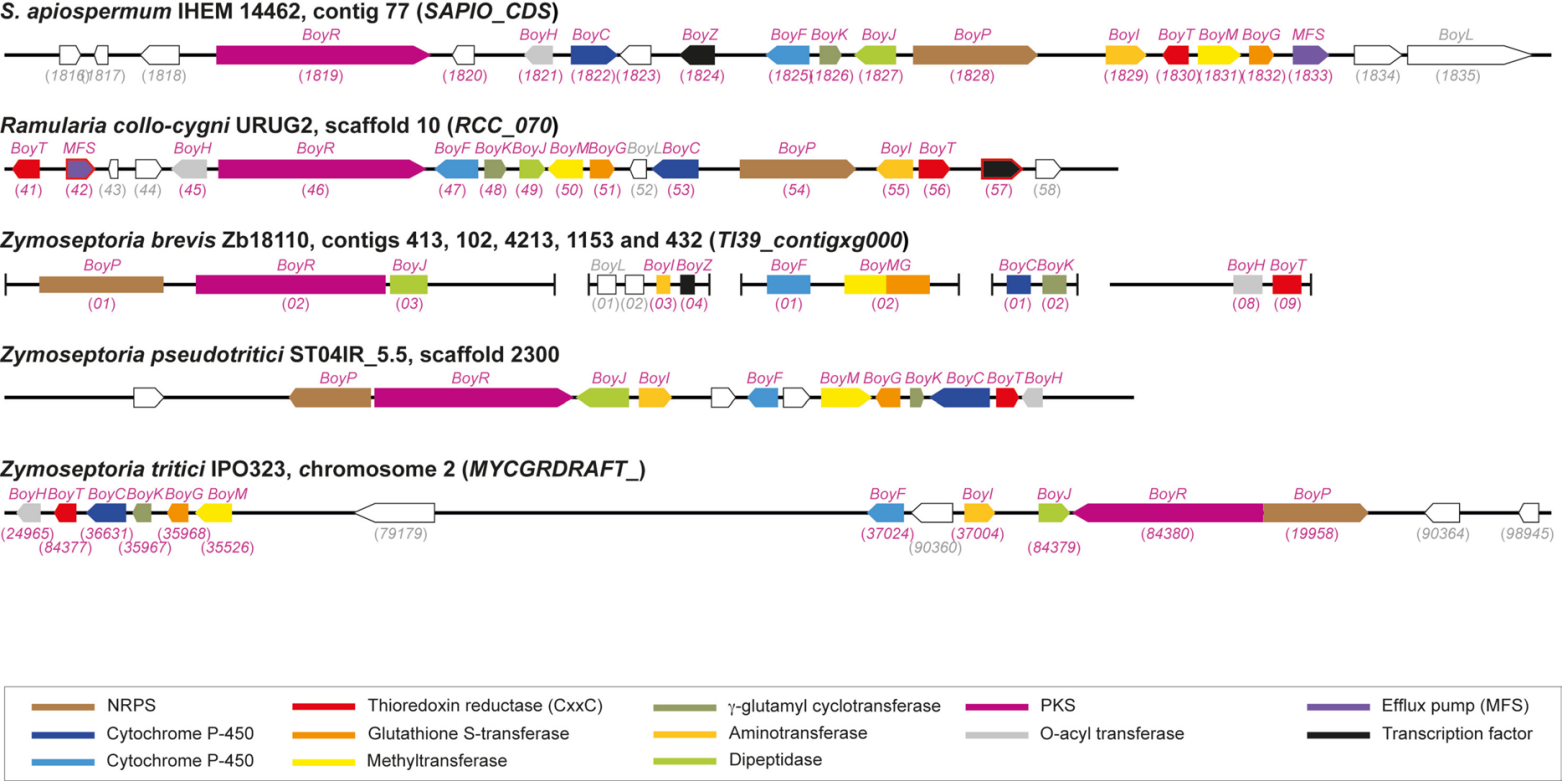
The boydines biosynthetic gene cluster in some Mycosphaerellaceae. Almost all the genes of the *S. apiospermum* boydines BGC were found in the genome of some filamentous fungi belonging to the Mycosphaerellaceae family: *Ramularia collo-cygni,* and several *Zymoseptoria* species*,* such as *Z. brevis, Z. pseudotritici* and *Z. tritici.* Compared to *S. apiospermum* IHEM14462, the SAM-dependent methyltransferase and glutathione S-transferase activities were supported by the same protein, BoyMG, in *Z. brevis*. Likewise, the gene cluster comprised two TrxR-encoding genes for *R. collo-cygni*, but the two corresponding proteins exhibited the canonical peptide sequence CxxC. In addition, all our attempts to identify this BGC in the genome of the other annotated genomes available for Mycosphaerellaceae (including *Cercospora beticola*, *Cercospora kikuchii*, *Cercospora zeae-maydis, Dothistroma septosporum*, *Fulvia fulva*, *Lecanosticta acicola, Pseudocercospora fijiensis*, *Pseudocercospora fuligena*, *Septoria linicola*, *Sphaerulina musiva* and *Zasmidium cellare*) were unsuccessful. GenBank accession numbers of the genes are indicated in parentheses (in pink for members of the BGC that are named *Boy* for boydines biosynthesis, in grey for non-members), except for *Z. pseudotritici* since its genome is not annotated. Genes without ortholog in *S. apiospermum* boydines BGC are outlined in red. PKS: polyketide synthase; MFS: major facilitator superfamily; NRPS: non-ribosomal peptide synthase

BGCs comprising orthologs of almost all genes of the boydines BGC in *S. apiospermum* were found in *C. caudatum* and* C. musicola,* as well as in* I. caudata* and* T. curvatum* (Table 3 and Fig. 6). Likewise, the boydines BGC was also identified in the genomes of* R. collo-cygni, Z. brevis*, and *Z. tritici* (Table 3 and Fig. 7)*.* Conversely, all our attempts to identify this BGC in the genomes of other *Colletotrichum* and *Zymoseptoria* species were unsuccessful, except for *C. cliviicola* strain YN31 and *Z. pseudotritici* strain ST04IR_5.5, nor in the other annotated genomes available for Mycosphaerellaceae. Of note, as observed for *S. apiospermum* strains HDO1 and 2RF1-5, both SAM-dependent methyltransferase and glutathione S-transferase activities were supported by the same protein BoyMG for *C. caudatum*, *I. caudata* and *Z. brevis*. For the latter, the orthologs were found in different contigs, but PCR targeting the ends of the contigs confirmed the joining of the contigs and allowed us to reconstruct the BGC (Supplementary Figure S4).

**Table 3:** The boydines biosynthetic gene cluster in other Sordariomycetes and some Mycosphaerelliaceae

Species and strain	AntiSMASH analysis			Number of genes identified by BLAST searches				
	BGC location	Metabolite predicted	Gene number	Total	TrxR	P450	MFS	TF
*Scedosporium apiospermum* IHEM 14462	Contig 77	Aspirochlorine	9	15	1 (CxxC)	2	1 (2.A.1.16)	1 (Zn_6_Cys_6_)
*Colletotrichum caudatum* CBS 131602	Scaffold 298 (and 473)	Penigainamide/outivirin/pretrichrodermamide	9	15 (with *BoyPI * and *BoyMG*)	2 (both CxxC)	4	1 (2.A.1.16)	1
*Colletotrichum cliviicola* YN31*	Contig 9	Acetylaranotin	11	13	1 (CxxC)	2	1	1
*Colletotrichum musicola* LFN0074	Contig 120	Acetylaranotin	7	16	2 (both CxxC)	4	2 (2.A.1.16 and 2.A.1.3)	1
	Contig 672	Penigainamide/outivirin/pretrichrodermamide	8					
*Immersiella caudata* CBS 106.72	Scaffold 5	Acetylaranotin	13	17	2 (both CxxC)	4	2 (2.A.1.3 and 2.A.1.16)	1 (Zn_6_Cys_6_)
*Sarocladium implicatum* TR	Scaffold 5	Acetylaranotin	9	12 (with *BoyMG*)	1 (CxxC)	3	ND	1
*Thyridium curvatum D216*	Nodes 8	Acetylaranotin	9	15	1 (CxxC)	3	2 (2.A.1.16 and 2.A.1.3)	1
	and 102	Aspirochlorine	5					
*Ramularia collo-cygni URUG2*	Scaffold 10	Penigainamide/outivirin/pretrichrodermamide	15	14	2 (both CxxC)	2	1 (2.A.1.3)	1 (Zn6Cys6)
*Zymoseptoria brevis* Zb18110	Contig 413 (and 102, 4213, 1153 and 432)	No prediction	2	11 (with *BoyMG*)	1 (CxxC)	2	ND	1 (Zn_6_Cys_6_)
*Zymoseptoria tritici* IPO323	Chromosome 2	Acetylaranotin	6	11	1 (CxxC)	2	ND	ND

^*^Genome not annotated

No ortholog of *SAPIO_CDS1820* was found in the other genomes studied here. The ortholog of *BoyK* in the genome of *C. musicola* was not annotated

MFS: major facilitator superfamily; ND: not detected; TF: transcription factor; TrxR: thioredoxine reductase

Interestingly, two genes encoding TrxRs were identified in the boydines BGC of *C. caudatum*, *C. musicola*, *I. caudata* and *R. collo-cygni*. In each case, both proteins exhibited the canonical peptide sequence CxxC. In addition, the PKSs detected in the boydines BGCs of these fungal species exhibited a similar domain architecture (KS-AT-DH-cMT-ER-KR-PP), and analysis of the peptide sequence of the PP domain suggested the assembly of three condensation units (Fig. 2).

Finally, *Sarocladium implicatum* strain TR exhibited a very similar BGC, suggesting the production of secondary metabolites closely related to boydines, but without the methyl groups branched on the alkyl chain, since the *S. implicatum* ortholog of the PKS KEZ45498 was devoid of cMT domain. Besides, analysis of the exons-introns boundaries of the PKS-encoding gene in all these fungi revealed high conservation of the first four exons, except for *S. implicatum* (Supplementary Figure S5).

### Genes of the Boydines Biosynthesis Cluster are Co-Regulated

**Fig. 8:**
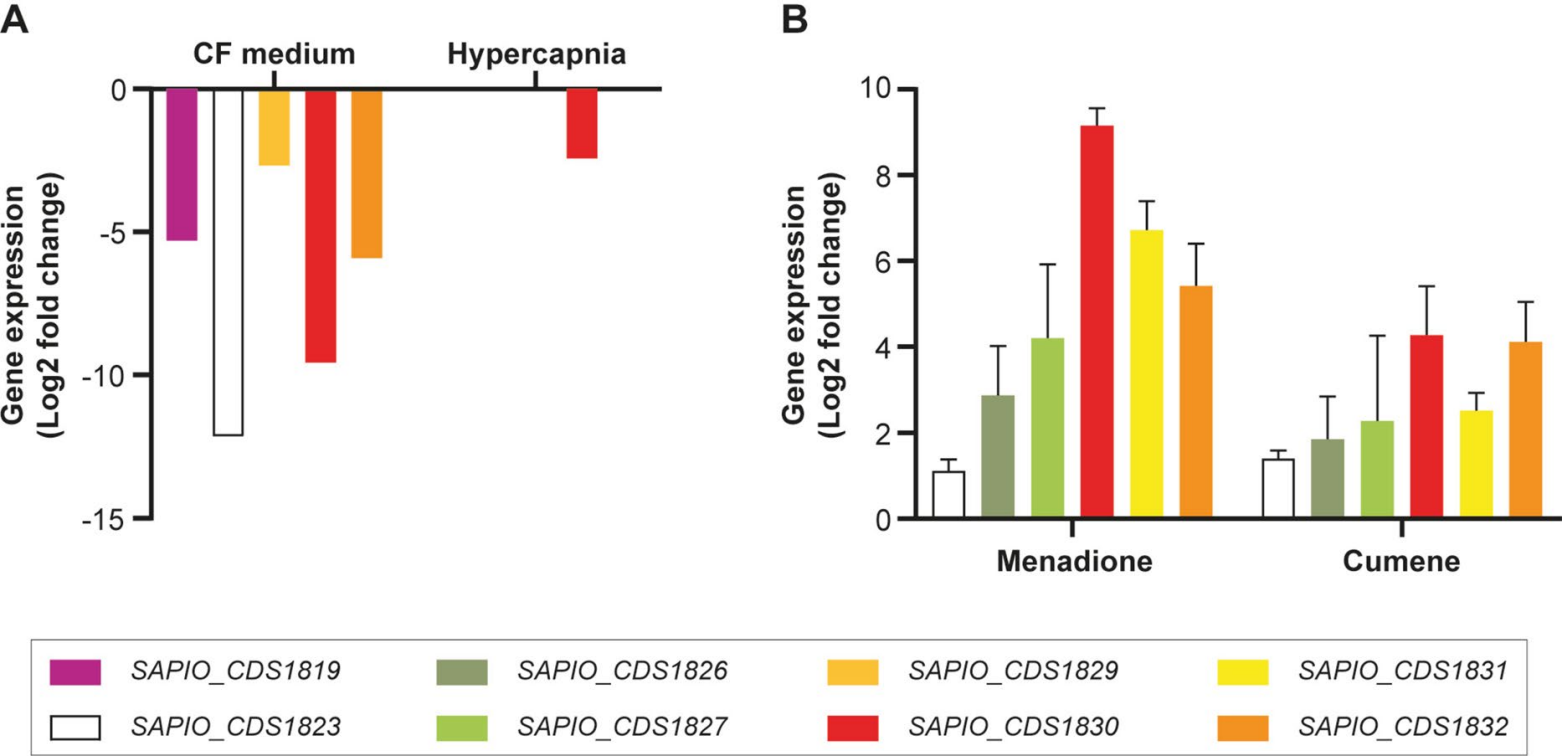
Expression level of genes of the *Scedosporium apiospermum* boydines biosynthetic gene cluster. The expression level of these genes was evaluated in mycelium from cultures grown in a CF synthetic liquid medium, or in YPD broth incubated under hypercapnic conditions (**A**), to mimic the saprobic growth of the fungus within a climax microbial community, or after exposure of the fungus to oxidative stress induced by menadione or cumene hydroperoxide (**B**), to mimic the environment of the fungus during pulmonary exacerbations

Transcriptomic data from a previous study focusing on the adaptation of *S. apiospermum* strain IHEM 14462 to the physicochemical conditions encountered in the CF bronchial mucus were further analyzed regarding the expression level of genes belonging to the boydines BGC. Transfrags corresponding to all but three of the genes of this cluster (*SAPIO_CDS1820*, *SAPIO_CDS1827* and *SAPIO_CDS1833*) were detected when the fungus was grown under normal air conditions. As previously reported for genes involved in the biosynthesis of aflatoxin-like mycotoxins, 5 out of the 15 members of the boydines BGC were down-regulated when the fungus was cultivated in a CF synthetic medium and 1 of them (*SAPIO_CDS1830* encoding the TrxR) was down-regulated under hypercapnic conditions (Fig. 8A).

In contrast, exposure of the fungus to a chemically-induced oxidative stress resulted in the overexpression of the six studied genes (Fig. 8B). Log2 fold changes ranging from 1.41 to 4.27 were seen in the expression level of these genes in response to cumene hydroperoxide, and this overexpression was even higher in cultures grown in the presence of menadione.

## Discussion

Over the past two decades, particular attention has been given to secondary metabolites produced by human fungal pathogens due to their potential role in the pathogenesis of infection. Nearly 170 secondary metabolites belonging to different chemical classes, mainly terpenoids, polyketides, and NRPs, have been identified in *Scedosporium* species. Twelve of these NRPs, including boydines A to D, derive from the assembly of two phenylalanine molecules [21, 22]. However, knowledge about the genes involved in their biosynthesis and the architecture of metabolic pathways remains fragmentary.

Two of the nine NRPSs identified in the genome of *S. apiospermum* display a single adenylation domain and two condensation domains [6]. These NRPSs could, therefore, drive the synthesis of boydines, which are related to acetylaranotin produced by *A. terreus*. One of these NRPSs, SidD, drives the synthesis of *N*^α^-methylcoprogen B, whose role in iron acquisition has been clearly demonstrated [4]. According to antiSMASH, *SAPIO_CDS1828* which encodes the second NRPS with a single adenylation domain, is part of a BGC comprising 9 genes predicted to ensure the synthesis of aspirochlorine, another ETP also resulting from the assembly of two phenylalanine molecules.

Usually in fungi, genes involved in the biosynthesis of secondary metabolites are physically linked in gene clusters, which allows co-regulation of gene expression and metabolite production and efflux [33]. The *S. apiospermum* BGC centered on *SAPIO_CDS1828* comprises all the genes encoding the decorating enzymes required for the hydroxylation of the cyclodipeptide backbone, the subsequent substitution of thiol groups to the hydroxyles and formation of the disulfide bridge. All these steps are common to acetylaranotin and aspirochlorine biosynthesis [19, 20, 23, 24]. Nevertheless, none of the genes required for the final steps of aspirochlorine synthesis (i.e. migration of one of the sulfur atoms of the disulfide bridge and spiroformation, degradation of one of the phenylanyl residue and its conversion to glycyl, and finally halogenation of the remaining phenylalanyl residue), had an ortholog in the *S. apiospermum* “aspirochlorine” BGC. As it occurs in the acetylaranotin BGC in *A. terreus*, only one TrxR-encoding gene was found in the *S. apiospermum* “aspirochlorine” BGC. For both fungi, the encoded protein displays the canonical peptide motif CxxC, whereas a second TrxR with a CxxH or CxxQ motif [34] is required for the migration of the disulfide bridge from α,α’- to α,β’-positions and spiroformation that occur in aspirochlorine. Likewise, the *S. apiospermum* “aspirochlorine” BGC comprised a limited number of cytochrome-encoding genes, only two (*SAPIO_CDS1822* and *SAPIO_CDS1825,* orthologs of *AtaTC* / *AclC* and of *AtaF* / *AclB,* respectively)*,* thus contrasting with the high number of these genes required for aspirochlorine synthesis [24]. However, conversely to the acetylaranotin BGC in *A. terreus*, the *S. apiospermum* “aspirochlorine” BGC also comprised a gene encoding a PKS (*SAPIO_CDS1819*), with the KS-AT-DH-cMT-ER-KR-PP domain architecture. Moreover, analysis of the amino acid sequence of its PP and ER domains suggested the assembly of three malonyl-CoA units and the inefficiency of the ER domain. These results are consistent with the synthesis of 3-hydroxy-2,4,6-trimethyl-5-oxooct-6-enoic acid, the alkyl chain of boydines. Finally, this BGC also comprised two genes encoding a class III zinc-binding transcription factor, and an efflux protein belonging to family 16 of the MFS, which is consistent with the identification of boydines from culture filtrates of some *Scedosporium* strains grown in YPD or modified Czapek-Dox broth [21, 22]. In summary, the *S. apiospermum* “aspirochlorine” BGC actually comprises 15 genes that ensure the synthesis of hybrid compounds, the boydines. Strikingly, despite the presence of a TrxR-encoding gene in this BGC, none of the aranotin-related compounds already identified in *Scedosporium* cultures exhibited a disulfide bridge. Given the high reactivity of these compounds, one may speculate the opening of this disulfide bridge during the extraction procedures, and the subsequent methylation of the thiol groups.

Hybrid secondary metabolites resulting from the assembly of a polyketide with a terpene or NRP exhibit a huge potential as pharmaceutical drugs [35–37]. For example, mycophenolic acid, which is widely used as immunosuppressant in bone marrow and solid organ transplantation [35], is a meroterpenoid produced by a restricted number of Eurotiales species comprising a linear sesquiterpene tail branched to a cyclic polyketide [38]. Fumagillin produced by *Aspergillus fumigatus,* which shows a linear polyketide chain branched to a cyclic sesquiterpene, is used to treat microsporidiosis in humans and honeybees [35]. Likewise, the echinocandins, the most recent class of antifungal drugs, derive from the lipopeptide pneumocandin B, produced by *Glarea lozoyensis,* and composed of a polyketide tail linked to a cyclohexapeptide [36, 37]. Regarding boydines, antibacterial activity is the sole biological activity that has been studied so far [21]. Their biological activities should be further investigated and compared to that of aranotin, since the introduction of a partially reduced linear polyketide chain may enhance the affinity for the host cell membranes.

The boydines BGC is a common feature of all *Scedosporium* species. Orthologs of all the genes in this BGC were found in the four other available genomes for *S. apiospermum*, and the cluster organization was perfectly conserved in two of them, *i.e.* the endophytic strain 2RF1-5 collected in Slovakia [39] and the strain HDO1, isolated from soil in Columbia [40]. The poor quality of the assembly of sequence data did not allow us to determine the organization of these genes in cluster for the two other genomes. This BGC was also identified in the genome of the other *Scedosporium* species that we studied. Synteny was perfectly conserved in the genome of *S. boydii* IHEM23826 [41] and *S. aurantiacum* MUT 6114 [42], whereas a chromosomal rearrangement was observed in the less pathogenic species *S. minutisporum* MUT 6113 [42] and *S. dehoogii* UA120008799-01/4 [43].

Moreover, boydines could be produced also by non-*Scedosporium* molds. Among the fungal genomes studied, we found that the boydines BGC was shared with other Sordariomycetes: (i) *Immersiella caudata*, formerly *Sphaeria caudata* or *Cercophora caudata*, which grows in very loose, porous wood [44]; (ii) *Thyridium curvatum*, formerly *Phialemoniopsis curvata* or *Phialemonium curvatum* [45]*,* which can grow on biodiesel [46], like *Scedosporium* species [47–49]; and (iii), several *Colletotrichum* species, *i.e. C. caudatum, C. cliviicola* and *C. musicola*, which cause leaf spot disease in various plant hosts, including indiangrass, cowpea, tobacco, manioc, and litchi [50–54].

Strikingly, this BGC was also found in the genome of some Mycosphaerelliaceae, including *Ramularia collo-cygni*, an emerging pathogen causing the leaf spot disease of barley [55], as well as some *Zymoseptoria* species (*Z. brevis* and *Z. tritici*) that affect graminicolous hosts [56, 57].

The presence of the boydines BGC in these phylogenetically distant species suggests that *Scedosporium* species share the same natural habitat with these Sordariomycetes or Mycosphaerelliaceae. Whether this BGC was an ancestral gene cluster distributed by vertical transmission and subsequent gene loss in most species, or acquired by horizontal gene transfer between species living in the same ecological niche, these fungi have likely acquired or maintained similar traits to adapt to environmental constraints. Consistent with this hypothesis, several *Scedosporium* strains have been found living as endophytes in *Bauhinia guianensis*, *Eucalyptus exserta* or *Hypericum maculatum* [39, 58, 59] while others have been recovered from submerged decaying wood in estuaries and on marine coasts [60, 61]*,* wood pulp [62, 63], forest soils in Belgium, Spain or Zaire [63] and xylophagous insects or termite nests in French Guyana, China, Costa Rica or India [21, 64–66]. Likewise, recent studies demonstrated that *Scedosporium* species may use lignin as the sole source of carbon and energy, and possess all the enzymatic equipment necessary for depolymerization of lignin and the subsequent opening of the aromatic rings [67, 68], which also allows them to use aromatic pollutants as a carbon source for their growth. Finally, a large phylogenetic analysis based on whole-genome comparisons revealed that, within the Sordariomycetes class, *Scedosporium* species form a clade with the Ceratocystidaceae family, which consists of plant pathogenic fungi living on roots (*Berkeleyomyces* spp., some *Ceratocystis* species, *Thielaviopsis* spp.), and saproxylophagous or xylophagous species (*Bretziella fagacearum*, other *Ceratocystis* species, *Chalaropsis* spp., *Davidsionella* spp., *Huntiella* spp., ambrosia fungi belonging to the genus *Ambrosiella, Meredithiella, Phialophoropsis, Solaloca, Toshionella* or *Wolfgangiella*, and bark-beetle-associated fungi of the genus *Endoconidiophora*) [69, 70].

The genes involved in the biosynthesis of boydines are co-regulated. A typical Gal4-type transcription factor was identified within the cluster for most species harboring the boydines BGC. Regarding *S. apiospermum* IHEM14462, GENSCAN analysis of the nucleotide sequence of *SAPIO_CDS1824* and PANNZER2 analysis of the amino acid sequence of the corresponding protein KEZ45502 revealed an erroneous identification of the exons-introns boundaries within this gene and the presence in the encoded protein of six cysteinyl residues with a Cx_2_Cx_6_Cx_6_Cx_2_Cx_6_C spacing, which is consistent with the canonical spacing of cysteinyl residues in Gal4-type Zn_2_Cys_6_ transcription factors, Cx_2_Cx_6_Cx_5–12_Cx_2_Cx_6–9_C [71]. This is not the only error in the genome of *S. apiospermum* IHEM14462. As mentioned above, about 2,000 coding sequences were erroneously considered as pseudogenes, like *SidD*, which plays a key role in fungal pathogenesis [4]. Another example is provided by the peroxiredoxin-encoding gene *SaPrx2* involved in the degradation of reactive oxygen species, which was not identified as a coding sequence during the annotation of the draft genome sequence of *S. apiospermum* with Augustus [72]. Moreover, an error in the exons-introns boundaries has already been demonstrated for *SAPIO_CDS2796* [73]. Such errors may have occurred in the annotation of other fungal genomes. For instance, the *BoyR* ortholog in *Z. tritici* and *Z. brevis* comprises an unusually long intron at its 5’-end for the former, while the first four exons, highly conserved in *BoyR* orthologs, are lacking in the latter.

As previously reported for genes involved in the synthesis of the aflatoxin-related metabolite O-methyl sterigmatocystin [29], several members of the *S. apiospermum* boydines BGC were underexpressed in cultures grown in a CF synthetic culture medium. The reprogramming of the secondary metabolism of the fungus in response to the physicochemical conditions encountered in the CF bronchial mucus may explain the usual absence of clinical translation of the chronic colonization of the airways by *Scedosporium* species. Environmental conditions also shape phenotypic changes in the bacterial pathogen *Pseudomonas aeruginosa* during long-term colonization of the CF lung*,* with a reduced synthesis of molecules of the quorum-sensing repertoire or even loss of nonessential metabolic functions [74, 75]. By silencing the biosynthetic genes, microorganisms limit the synthesis of secondary metabolites and their potential toxic effects on the host epithelial cells, and avoid the host immune response, thus enabling their saprobic growth within a climax microbial community in clinically stable CF patients [76].

Conversely, all studied members of the *S. apiospermum* boydines BGC were found to be overexpressed upon exposure to menadione, a generator of superoxide anions, and to cumene hydroperoxide, which more specifically solicits components of the thioredoxin system [77]. Of the 33 genes potentially involved in the degradation of reactive oxygen or nitrogen species, analysis of the transcriptional response of the fungus upon exposure to chemically induced oxidative stresses or co-cultivation with phagocytic cells showed that *SAPIO_CDS1830*, encoding the TrxR within the boydines BGC, was one of the three most overexpressed genes [5].

Together these results clearly establish that the BGC centered on *SAPIO_CDS1828* in *S. apiospermum* IHEM14462, actually a 15-membered gene cluster, ensures the synthesis of some aranotin-related compounds. These secondary metabolites, called boydines, have already shown antibacterial activity and may also play an important role in fungal cells to cope with oxygen radicals, as suggested by the overexpression of several genes of this BGC in response to oxidative stress. Therefore, phenotypic changes resulting from disruption of the *SAPIO_CDS1830* gene and the associated NRPS-encoding gene (*SAPIO_CDS1828)* should be investigated to confirm the role of these enzymes in evasion to the host immune response during pulmonary exacerbations.

## Supplementary Information

Below is the link to the electronic supplementary material.

Supplementary file1 (PDF 694 KB)

Supplementary file2 (PDF 592 KB)

## Data Availability

Data is provided within the manuscript or supplementary information files.
