# Supplementary material for: The Biosynthetic Gene Cluster of Boydines in Scedosporium apiospermum
Source: Mycopathologia. 2026 Feb 7;191(2):31. doi: 10.1007/s11046-026-01050-z (PMC12881047; doi:10.1007/s11046-026-01050-z)

The biosynthetic gene cluster of boydines in *Scedosporium apiospermum*

Clarisse Carvalho, Anaïs Hérivaux, Méline Wéry, Jean-Charles Jouhanneau, Nicolas Papon, and Jean-Philippe Bouchara

Corresponding authors: Clarisse Carvalho (clarisse.carvalho@univ-angers.fr) and Jean-Philippe Bouchara (jean-philippe.bouchara@univ-angers.fr)

IRF (Infections Respiratoires Fongiques), Univ Angers, Univ Brest, SFR ICAT 4208, Angers, France

Supplementary Figures

Supplementary Figure S1: Biosynthetic pathways for acetylaranotin synthesis in *A. terreus* NIH 2624 and aspirochlorine synthesis in *A. oryzae* RIB40, together with their respective BGCs. GenBank accession numbers of the genes are indicated in parentheses (members of the BGC in red for *A. terreus* and blue for *A. oryzae*, in grey for non-members).

PKS: polyketide synthase; MFS: major facilitator superfamily; NRPS: non-ribosomal peptide synthase; SAM: S-adenosylmethionine.

Supplementary Figure S2: The boydines biosynthetic gene cluster in *Scedosporium minutisporum* MUT 6113. Compared to the *S. apiospermum* boydines BGC, a chromosomal rearrangement was observed in *S. minutisporum*, which was confirmed by PCR and sequencing using primers targeting the end of the MFS-encoding gene and *BoyJ*. In addition, PCR and sequencing with primers targeting the 3’-end of node 182 and the 5’-end of node 98 confirmed their joining.

Supplementary Figure S3: The boydines biosynthetic gene cluster in *Scedosporium dehoogii* strain UA120008799-01/4. Compared to the *S. apiospermum* boydines BGC, a chromosomal rearrangement was observed in *S. dehoogii*, which was confirmed by PCR and sequencing using primers targeting the end of the MFS-encoding gene and *BoyJ*.

Supplementary Figure S4: The boydines biosynthetic gene cluster in *Zymoseptoria brevis* Zb18110. The genes of the boydines BGC in *Z. brevis* were distributed over several contigs, most of them comprising only 2 to 4 genes. PCR amplification using primers targeting the ends of contigs 102 and 4213, 4213 and 1153, or 1153 and 432 confirmed their joining.

Supplementary Figure S5: Exons-introns boundaries in the PKS-encoding gene identified in the boydines biosynthetic gene cluster (BGC) in *S. apiospermum* IHEM 14462 and its orthologs. Apart from *SAPIO_CDS1819* encoding the PKS KEZ45498, the reference genome of *S. apiospermum* comprises three other genes encoding PKSs with the same KS-AT-DH-cMT-ER-KR-PP domain architecture. However, the exons-introns boundaries in these four genes were totally different. In contrast, a high conservation of the exons-introns boundaries was observed between *SAPIO_CDS1819* and its orthologs, especially in the length of the first four exons, except for the *LIA77_06636* PKS-encoding gene in *Sarocladium implicatum* strain TR.

Supplementary Figure S1

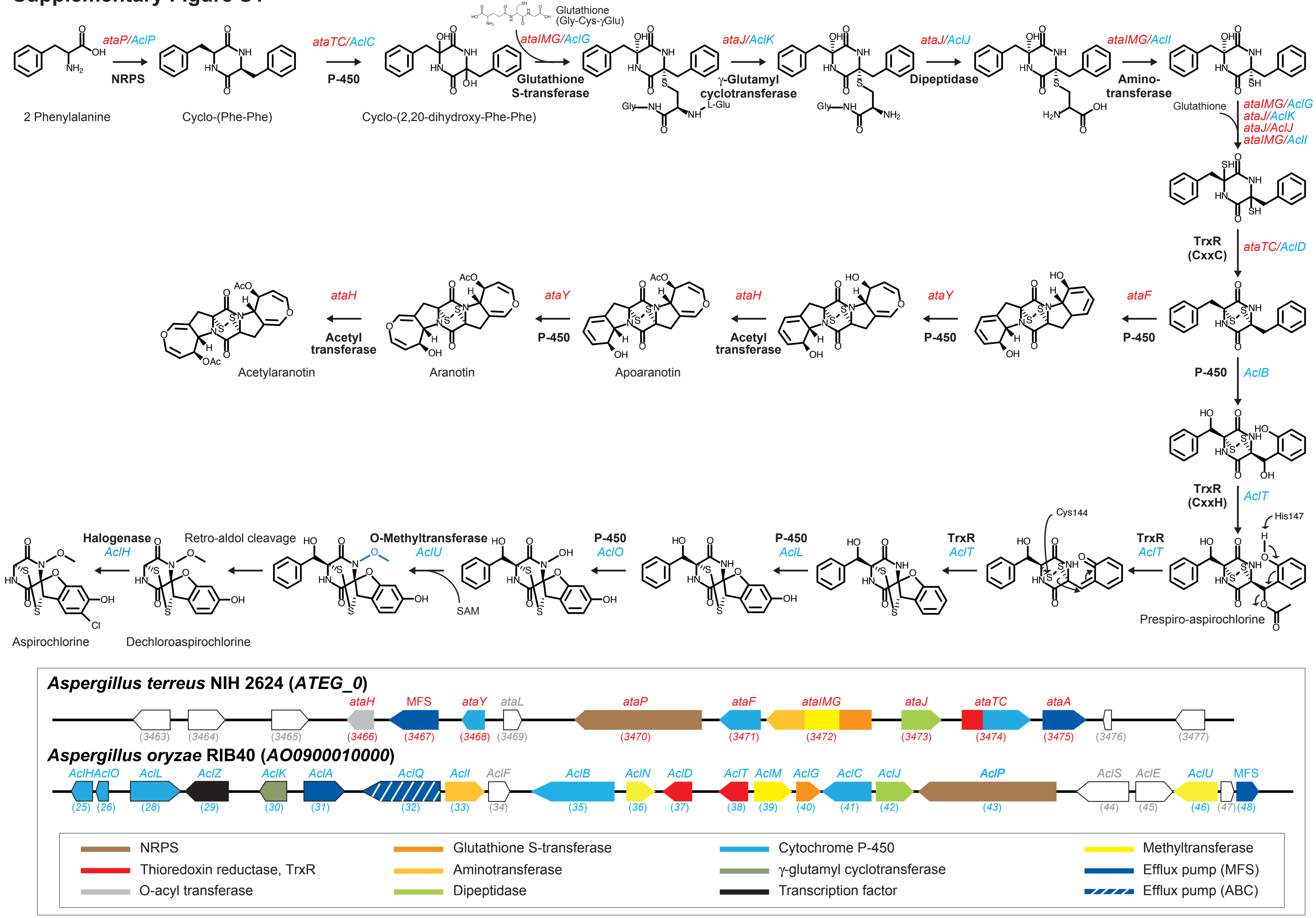

Supplementary Figure S2

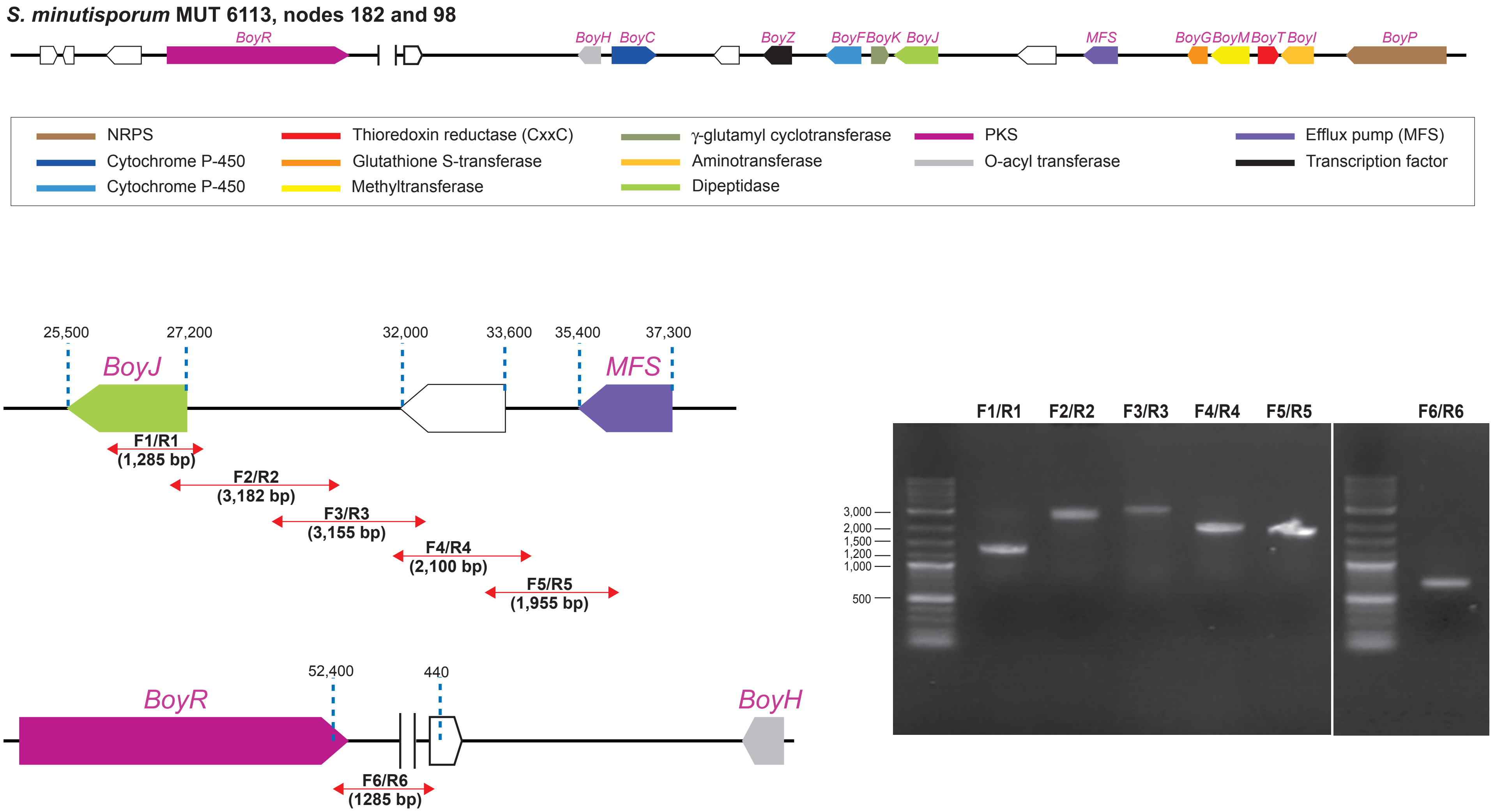

Supplementary Figure S3

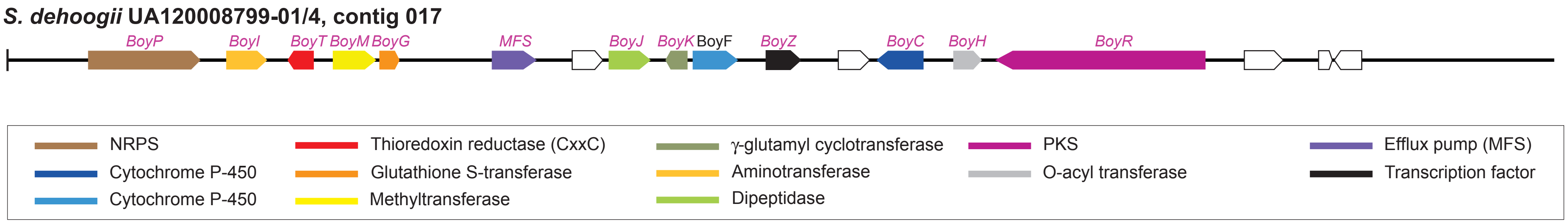

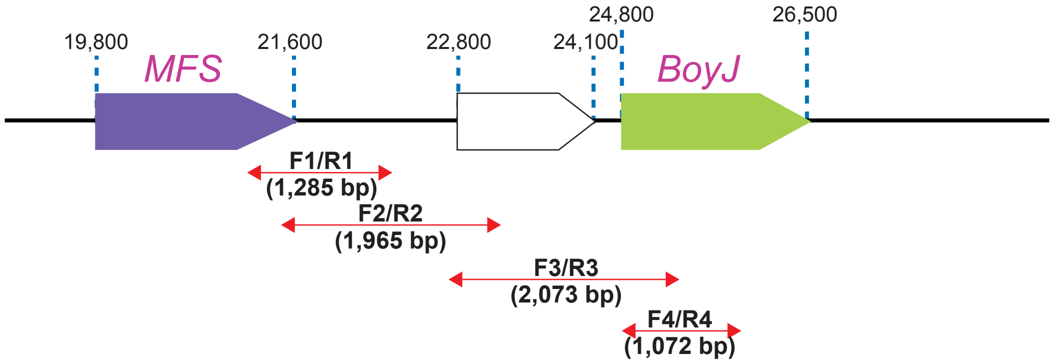

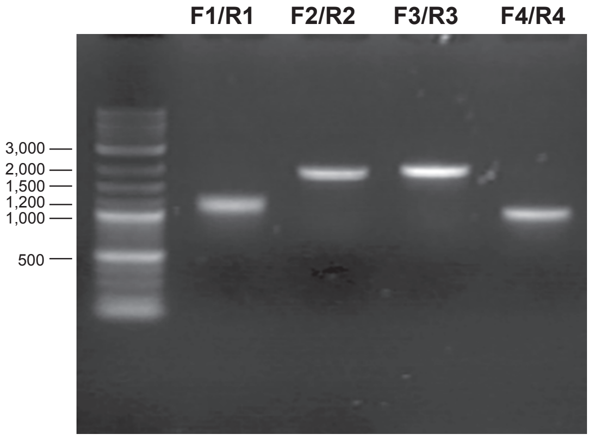

Supplementary Figure S4

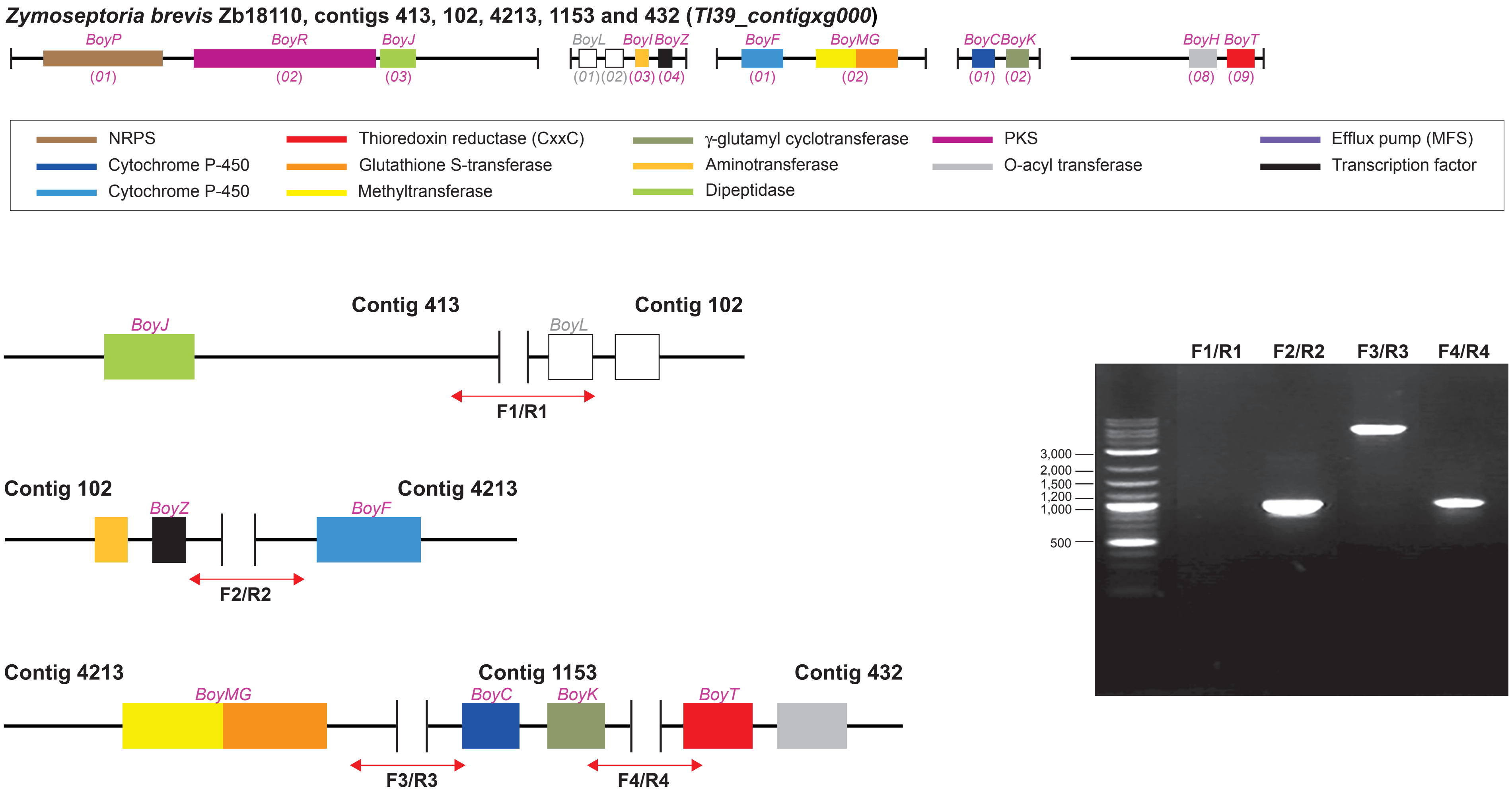

Supplementary Figure S5

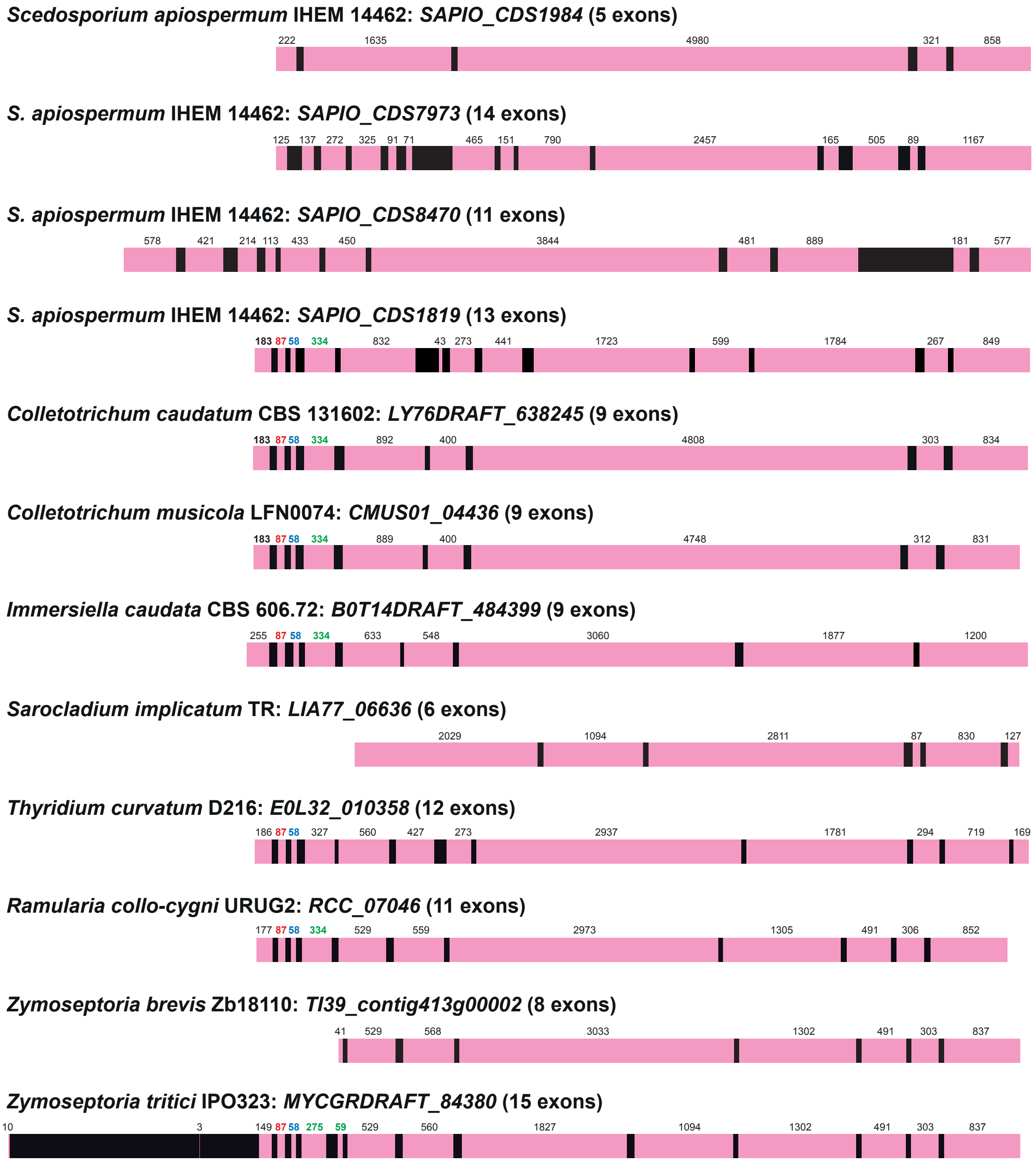

Supplement: Supplementary file 1 — Supplementary file1 (PDF 694 KB) [file 11046_2026_1050_MOESM1_ESM.pdf]
