# Supplementary material for: The Biosynthetic Gene Cluster of Boydines in Scedosporium apiospermum
Source: Mycopathologia. 2026 Feb 7;191(2):31. doi: 10.1007/s11046-026-01050-z (PMC12881047; doi:10.1007/s11046-026-01050-z)
Supplement: Supplementary file 2 — Supplementary file2 (PDF 592 KB) [file 11046_2026_1050_MOESM2_ESM.pdf]

The biosynthetic gene cluster of boydines in *Scedosporium apiospermum*

Clarisse Carvalho, Anaïs Hérivaux, Méline Wéry, Jean-Charles Jouhanneau, Nicolas Papon, and Jean-Philippe Bouchara

Corresponding authors: Clarisse Carvalho (clarisse.carvalho@univ-angers.fr) and Jean-Philippe Bouchara (jean-philippe.bouchara@univ-angers.fr)

IRF (Infections Respiratoires Fongiques), Univ Angers, Univ Brest, SFR ICAT 4208, Angers, France

Supplementary Tables

Supplementary Table S1: List of fungal species studied.

Supplementary Table S2: List of the NRPSs identified by antiSMASH analysis as having a single adenylation domain and two condensation domains, therefore possibly involved in the synthesis of homodipeptides.

Supplementary Table S3: The amino acid sequence of the acyl carrier protein domain (PP domain) of selected PKSs allowing the assembly of a well-defined number of condensation units, and of KEZ45498 and its orthologs.

Supplementary Table S4: Selected iterative PKSs exhibiting a functional ER domain and comparison with that of the ER domain of KEZ45498 and its orthologs in *Scedosporium* species.

Supplementary Table S5: List of the primers used.

Supplementary Table S1: List of fungal species studied.

Fungal species	Submitted Genbank assembly	Genbank accession number	Genome size (Mb)	Number of scaffolds	Number of contigs	Number of regions detected by anti-SMASH analysis
Aaosphaeria arxii CBS 175.79	GCA_010015735.1	JAADKL000000000.1	38.9	55	235	64
Acarospora aff. strigata isolate T1882	GCA_022814355.1	JALDZA000000000.1	28.7	475	503	22
Akanthomyces lecanii strain RCEF 1005	GCA_001636795.1	AZHF00000000.1	35.6	130	197	52
Akanthomyces muscarius Ve6	GCA_028009165.1	JAJHUN000000000.1	36.2	12	12	57
Alectoria sarmentosa Spiribille	GCA_904859925.1	CAJHES000000000.1	53.3	1,135	1,323	83
Amauroascus aureus (formerly *Arachniotus aureus)*	Genome not available					
Amniculicola lignicola CBS 123094	GCA_010015725.1	JAADKK000000000.1	49.6	510	1,406	50
Amylocarpus encephaloides TRa018bII	GCA_019669835.1	JAFMPF000000000.1	46.3	2,381	2,508	51
Annulohypoxylon maeteangense CBS 123835	GCA_022496945.1	JAJKHO000000000.1	38.2	64	82	75
Annulohypoxylon moriforme CBS 123579	GCA_022695765.1	JAJLRE000000000.1	42.7	967	1,005	80
Annulohypoxylon stygium FL0470	GCA_022695755.1	JAJLRF000000000.1	38.5	278	285	77
Annulohypoxylon truncatum CBS 140777	GCA_022578515.1	JAJKKI000000000.1	38.1	130	163	74
Anthostomella pinea	GCA_963556695.1	CAUWAG000000000.1	53.8	20	20	91
Apiospora arundinis AAU 773	GCA_039105305.1	JAPCWZ000000000.1	48.7	9	9	94
Apiospora kogelbergensis	Genome not available					
Apiospora marii CBS 49790	GCA_038362585.1	JAQQWD000000000.1	51.1	15	15	82
Arthroderma uncinatum CBS 119779	GCA_011692745.1	JAACJD000000000.1	23.6	/	5	42
Aspergillus arachidicola CBS 117612	GCA_009193545.1	STFM00000000.1	39.8	451	538	89
Aspergillus aurantiobrunneus CBS 465.65	GCA_047715765.1	JBDQZS000000000.1	31.3	278	381	67
Aspergillus avenaceus IBT 18842	GCA_009193465.1	STFI00000000.1	33.8	1,528	1,705	72
Aspergillus awamori IFM 58123	GCA_003850985.1	BDHI00000000.1	38.6	/	33	93
Aspergillus brasiliensis CBS 101740	GCA_001889945.1	LJXV00000000.1	35.8	103	288	85
Aspergillus caelatus CBS 763.97	GCA_009193585.1	STFO00000000.1	40	729	814	102
Aspergillus campestris IBT 28561	GCA_002847485.1	MSFM00000000.1	28.3	/	62	57
Aspergillus candidus CBS 102.13	GCA_002847045.1	PKFS00000000.1	27.3	268	316	55
Aspergillus carlsbadensis CBS 123894	GCA_025688815.1	SWKC00000000.1	39.5	254	321	79
Aspergillus clavatus NRRL 1	GCA_000002715.1	AAKD00000000.3	27.9	143	231	51
Aspergillus costaricaensis CBS 115574	GCA_003184835.1	PSTH00000000.1	36.9	86	174	98
Aspergillus cristatus GZAAS20.1005	GCA_001717485.1	JXNT00000000.1	28.5	68	169	44
Aspergillus eucalypticola CBS 122712	GCA_003184535.1	MSFU00000000.1	34.8	/	131	82
Aspergillus felis CNM-CM7691	GCA_014281915.1	ACBAG000000000.1	32.4	1,927	1,939	84
Aspergillus flavus NRRL3357	GCA_014117465.1	GCF_014117465.1	37	8	16	87

Aspergillus floccosus CBS 116.37	GCA_048565125.1	JBDRAK000000000.1	30.6	224	265	90
Aspergillus foveolatus CBS 279.81	GCA_047715535.1	JBDRAE000000000.1	31.8	425	503	80
Aspergillus fumigatiaffinis CNM-CM6805	GCA_012656285.1	JAAAPX000000000.1	33.5	1,055	1,062	80
Aspergillus germanicus CBS 123887	GCA_045785125.1	JBDRAJ000000000.1	39.5	430	491	75
Aspergillus hiratsukae CNM-CM5793	GCA_014281905.1	JACBAD000000000.1	30.1	2,13	2,136	48
Aspergillus homomorphus CBS 101889	GCA_003184865.1	PSTJ00000000.1	34.1	152	247	84
Aspergillus karnatakaensis CBS 102800	GCA_047715665.1	JBDQZP000000000.1	36.3	239	285	88
Aspergillus luchuensis IFO 4308	GCA_016861625.1	GCF_016861625.1	37.3	8 chromosomes		89
Aspergillus minisclerotigenes MRI390	GCA_028505775.1	JAHXGP000000000.1	38	28	220	56*
Aspergillus navahoensis CBS 351.81	GCA_048626375.1	JBDRAB000000000.1	32.8	731	814	78
Aspergillus neoniger CBS 115656	GCA_003184625.1	MSFP00000000.1	35.4	169	243	91
Aspergillus nidulans var. acristatus CBS 119.5	GCA_047715555.1	JBDRAF000000000.1	32.6	311	403	70
Aspergillus niger CBS 101883	GCA_003184595.1	MSFR00000000.1	35.9	107	197	93
Aspergillus novofumigatus IBT 16806	GCA_002847465.1	MSZS00000000.1	32.4	/	62	63
Aspergillus novoparasiticus CBS 126849	GCA_009176405.1	SWDA00000000.1	40.9	870	934	92
Aspergillus oryzae RIB40	GCA_000184455.3	GCF_000184455.2	37.9	11	28	79
Aspergillus parasiticus SU-1	GCA_000956085.1	JZEE00000000.1	39.5	/	753	60
Aspergillus phoenicis ATCC 13157	GCA_003344505.1	QQUR00000000.1	35.8	55	112	99
Aspergillus piperis CBS 112811	GCA_003184755.1	PSTD00000000.1	35.3	47	122	96
Aspergillus pseudocaelatus CBS 117616	GCA_009193665.1	STFS00000000.1	39.7	466	608	95
Aspergillus pseudotamarii CBS 117625	GCA_009193445.1	STFH00000000.1	38.2	249	365	95
Aspergillus pseudoustus CBS 123904	GCA_044049465.1	JBFXLU000000000.1	41.2	656	706	83
Aspergillus pseudoviridinutans IFM 55266	GCA_018340605.1	BHVY00000000.1	33.3	24	84	69
Aspergillus puulaauensis AS33	GCA_016861865.1	GCF_016861865.1	34.3	8	8	73
Aspergillus sclerotialis CBS 366.77	GCA_003589665.1	MVGC00000000.1	28	/	7,484	49
Aspergillus sclerotiicarbonarius CBS 121057	GCA_003184635.1	PSSZ00000000.1	37.6	166	277	87
Aspergillus sergii CBS 130017	GCA_009193525.1	STFL00000000.1	38.3	262	314	101
Aspergillus similis CBS 293.93	GCA_047715515.1	JBDRAC000000000.1	33.2	569	666	80
Aspergillus taichungensis IBT 19404	GCA_002850765.1	PKFW00000000.1	27.1	310	352	52
Aspergillus tamarii CBS 117626	GCA_009193485.1	STFJ00000000.1	38.5	448	511	52*
Aspergillus tanneri NIH1004	GCA_003426965.1	QUQM00000000.1	38.7	/	14	106
Aspergillus terreus NIH2624	GCA_000149615.1	AAJN00000000.1	29.3	26	267	77
Aspergillus terricola var. indicus CBS 952.97	GCA_047715525.1	JBDRAD000000000.1	32.5	601	678	76
Aspergillus tetrazonus CBS 591.65A	GCA_045791975.1	JBDRAG000000000.1	32.1	452	506	77
Aspergillus thermomutatus HMR AF 39	GCA_002237265.2	NKHU00000000.2	30.9	/	647	61
Aspergillus transmontanensis CBS 130015	GCA_009193505.1	STFK00000000.1	39.3	293	367	93
Aspergillus tubingensis WU-2223L	GCA_013340325.1	BLWE00000000.1	35	15	16	86
Aspergillus turcosus HMR AF 23	GCA_002234965.2	NKHV00000000.2	32.4	/	939	49
Aspergillus udagawae IFM 46973	GCA_001078395.2	BBXM00000000.2	32.3	17	136	71

Aspergillus ustus 3.3904	GCA_000812125.1	JOMC00000000.1	38.4	770	1,502	52
Aspergillus vadensis **CBS 113365**	**GCA_003184925.1**	**MSFS00000000.1**	**35.7**	**60**	**107**	**85**
Aspergillus varians **CBS 505.65**	**GCA_047715575.1**	**JBBNTZ000000000.1**	**29.8**	**427**	**533**	**54**
Aspergillus viridinutans **IFM 47045**	**GCA_018404265.1**	**BOPL00000000.1**	**34.9**	**47**	**385**	**59**
Aspergillus welwitschiae **CBS 139.54b**	**GCA_003344945.1**	**QQZQ00000000.1**	**37.5**	**396**	**514**	**96**
Bipolaris sorokiniana **ND90Pr**	**GCA_000338995.1**	**AEIN00000000.1**	**34.4**	**154**	**504**	**50**
Botryotinia calthae **MUCL2830**	**GCA_004379285.1**	**PHWZ00000000.1**	**47.4**	**3,978**	**4,002**	**59**
Botryotinia convoluta **MUCL11595**	**GCA_004786275.1**	**PQXN00000000.1**	**45.3**	**2,043**	**2,051**	**57**
Botryotinia globosa **MUCL 444**	**GCA_014898425.1**	**RCSZ00000000.1**	**45.7**	**/**	**27**	**52**
Botryotinia narcissicola **MUCL2120**	**GCA_004786225.1**	**PQXJ00000000.1**	**54.8**	**8,379**	**8,4**	**48**
Botrytis aclada **633**	**GCA_014898285.1**	**RCSV00000000.1**	**48.3**	**/**	**16**	**54**
Botrytis byssoidea **MUCL 94**	**GCA_014898295.1**	**RCSW00000000.1**	**43**	**/**	**59**	**50**
Botrytis cinerea BcDW1	GCA_000349525.1	AORW00000000.1	42.1	454	1,102	61
Botrytis deweyae **B1**	**GCA_014898535.1**	**RCSX00000000.1**	**44.4**	**/**	**76**	**61**
Botrytis elliptica Be9612	GCA_014898555.1	RCSY00000000.1	47.7	/	137	59
Botrytis fragariae **BVB16**	**GCA_013461495.1**	**JABFCT000000000.1**	**41.9**	**/**	**28**	**60**
Botrytis galanthina **MUCL435**	**GCA_004916875.1**	**PQXL00000000.1**	**43.9**	**3,417**	**3,431**	**68**
Botrytis hyacinthi **Bh0001**	**GCA_004786245.1**	**PQXK00000000.1**	**43.8**	**2,498**	**2,503**	**55**
Botrytis paeoniae **Bp0003**	**GCA_004786145.1**	**PQXI00000000.1**	**46.2**	**1,830**	**1,838**	**57**
Botrytis porri **MUCL 3234**	**GCA_014898465.1**	**RCTA00000000.1**	**46.8**	**/**	**31**	**46**
Botrytis sinoallii **Bc 23**	**GCA_014898435.1**	**RCTB00000000.1**	**61.3**	**/**	**47**	**58**
Botrytis tulipae **Bt9001**	**GCA_004786125.1**	**PQXH00000000.1**	**45.3**	**634**	**683**	**58**
Byssothecium circinans **CBS 675.92**	**GCA_010015675.1**	**JAADKH000000000.1**	**49.3**	**160**	**1,626**	**86**
Caloplaca aegaea **LIQ143CAAG**	**GCA_023646655.1**	**JALAIP000000000.1**	**31.5**	**/**	**6,484**	**28**
Camillea tinctor **CBS 203.56**	**GCA_022578605.1**	**JAJKKR000000000.1**	**45**	**229**	**554**	**73**
Cercospora kikuchii **MAFF 305040**	**GCA_019650295.1**	**BOLY00000000.1**	**34.4**	**/**	**9**	**71**
Ciborinia camelliae **ITAC2**	**GCA_025890175.1**	**JANPYM000000000.1**	**46.4**	**48**	**969**	**64**
Cladorrhinum samala **PSN324**	**GCA_033576145.1**	**JAUDZH000000000.1**	**36.2**	**359**	**652**	**47**
Clathrospora elynae **CBS 161.51**	**GCA_010015635.1**	**JAADKG000000000.1**	**37.4**	**617**	**2,230**	**47**
Colletotrichum asianum ICMP 18580	GCA_009806415.1	WOWK00000000.1	64.7	486	501	106
Colletotrichum caudatum **CBS 131602**	**GCA_030867225.1**	**JAHLTY000000000.1**	**44.2**	**1,257**	**1,630**	**82**
Colletotrichum chrysophilum **AFK26**	**GCA_026319265.1**	**JANUHP000000000.1**	**56**	**/**	**652**	**83**
Colletotrichum eremochloae **CBS 129661**	**GCA_030867245.1**	**JAHMUH000000000.1**	**47.2**	**403**	**547**	**90**
Colletotrichum fructicola **Nara gc5**	**GCA_000319635.2**	**ANPB00000000.2**	**59.5**	**/**	**12**	**94***
Colletotrichum gloeosporioides **Lc1**	**GCA_011800055.1**	**WVTB00000000.1**	**61.9**	**128**	**541**	**95**
Colletotrichum karsti **CkLH20**	**GCA_011947395.2**	**JAATWM000000000.2**	**51.9**	**127**	**203**	**65**
Colletotrichum musicola **LFN0074**	**GCA_014235935.1**	**WIGM00000000.1**	**52.7**	**2,463**	**2,791**	**76**
Colletotrichum noveboracense **PMBrms-1**	**GCA_026319125.1**	**JANUHK000000000.1**	**59.1**	**/**	**1,494**	**85**
Colletotrichum orbiculare **104-T**	**GCA_000350065.2**	**AMCV00000000.2**	**89.7**	**355**	**6,664**	**82**

***Colletotrichum siamense* Cg363**	**GCA_013390195.1**	**QPNA00000000.1**	**62.9**	**/**	**22**	**96**
***Colletotrichum spinosum* CBS 515.97**	**GCA_004366825.1**	**QAPG00000000.1**	**82.7**	**10,715**	**10,895**	**79**
Colletotrichum sublineola TX430BB	GCA_000696135.1	JMSE00000000.1	46.8	1,625	6,383	78
***Colletotrichum trifolii* 543-2**	**GCA_004367215.1**	**RYZW00000000.1**	**109.7**	**10,473**	**16,095**	**76**
***Colletotrichum zoysiae* MAFF 238573**	**GCA_030867305.1**	**JAHMAF000000000.1**	**46.5**	**617**	**840**	**75**
***Coniella lustricola* B22-T-1**	**GCA_003019895.1**	**NSBW00000000.1**	**36.6**	**634**	**985**	**45**
Corynespora cassiicola Philippines	GCA_003016335.1	NSJI00000000.1	44.8	244	644	70
***Cryphonectria parasitica* EP155**	**GCA 011745365.1**	**WHUS00000000.1**	**43.9**	**26**	**33**	**59**
***Cucurbitaria berberidis* CBS 394.84**	**GCA_010015615.1**	**JAADKF000000000.1**	**32.9**	**42**	**184**	**31**
***Cudoniella acicularis* DSM 108380**	**GCA_013054445.1**	**JAAMPI000000000.1**	**59**	**2799**	**2801**	**68**
***Daldinia bambusicola* CBS 122872**	**GCA_022478815.1**	**JAJMPD000000000.1**	**38**	**448**	**631**	**63**
***Daldinia caldariorum* CBS 122874**	**GCA 022478825.1**	**JAJNNR000000000.1**	**35.6**	**45**	**211**	**58**
***Daldinia childiae* JS-1345**	**GCA 008694065.1**	**VYXO00000000.1**	**38.7**	**133**	**282**	**59**
***Daldinia decipiens* CBS 113046**	**GCA_022478715.1**	**JAJNNS000000000.1**	**35.3**	**96**	**149**	**59**
***Daldinia eschscholtzii* MFLUCC 19-0629**	**GCA_037126485.1**	**JBANMG000000000.1**	**37.6**	**/**	**11**	**57**
***Daldinia grandis* CBS 114736**	**GCA_022432525.1**	**JAJKLB000000000.1**	**37.5**	**/**	**26**	**56**
***Daldinia loculata* CBS 113971**	**GCA_022478755.1**	**JAJNNT000000000.1**	**37.8**	**260**	**316**	**58**
***Daldinia vernicosa* CBS 139.73**	**GCA 022497035.1**	**JAJKKY000000000.1**	**35.8**	**275**	**310**	**63**
***Delitschia confertaspora* ATCC 74209**	**GCA_010093945.1**	**JAAEIU000000000.1**	**31.2**	**764**	**878**	**51**
***Diaporthe amygdali* CAA958**	**GCA_026229845.1**	**JAJATV000000000.1**	**51.5**	**267**	**271**	**111**
Diaporthe australafricana CMW 18300	GCA_042257625.1	JAWRVE000000000.1	50.8	505	536	95
***Diaporthe helianthi* 7/96**	**GCA_001702395.2**	**MAVT00000000.2**	**63.7**	**7,376**	**8,358**	**66**
***Didymosphaeria variabile* IMI 356815**	**GCA_027946475.1**	**JAPEUX000000000.1**	**39.5**	**/**	**11**	**57**
***Diplodia corticola* CBS 112549**	**GCA_001883845.1**	**MNUE00000000.1**	**35**	**181**	**286**	**44**
***Dothidotthia symphoricarpi* CBS 119687**	**GCA_010015815.1**	**JAADKQ000000000.1**	**34.4**	**59**	**349**	**42**
***Endocarpon pusillum* Z07020**	**GCA_000464535.1**	**APWS00000000.1**	**37.1**	**908**	**1731**	**41**
***Epicoccum nigrum* ICMP 19927**	**GCA_002116315.1**	**NCTX00000000.1**	**34.7**	**227**	**651**	**40**
Exophiala dermatitidis Ex12	GCA_028027665.1	JAJJCG000000000.1	23.6	49	91	15
Exophiala mesophila	GCA_004011775.1	NAJM00000000.1	30.4	196	228	15
Exophiala oligosperma CCFEE 6327	GCA_036872695.1	JAVRRE000000000.1	35.5	204	288	26
***Fusarium anthophilum* NRRL 25214**	**GCA 013364935.1**	**JABEVY000000000.1**	**45.8**	**1,117**	**1,126**	**69**
***Fusarium beomiforme* NRRL 25174**	**GCA 002980475.2**	**PVQB00000000.2**	**46.5**	**1,868**	**1,995**	**62**
***Fusarium bulbicola* NRRL 25176**	**GCA 013758895.1**	**JAAOAI000000000.1**	**43.6**	**1,719**	**1,758**	**61**
***Fusarium coicis* NRRL 66233**	**GCA 013781345.1**	**JAAOAJ000000000.1**	**42.7**	**1,267**	**1,296**	**56**
***Fusarium fujikuroi* IMI 58289**	**GCA 900079805.1**		**43.8**	**12 chromosomes**		**57**
***Fusarium gaditjirri* NRRL 45417**	**GCA 013266175.1**	**JABFAI000000000.1**	**41.9**	**834**	**859**	**59**
***Fusarium globosum* NRRL 26131**	**GCA_013396165.1**	**JAAQPF000000000.1**	**44.6**	**1,695**	**1,705**	**64**
***Fusarium mangiferae* MRC7560**	**GCA_900044065.1**	**FCQH00000000.1**	**46.3**	**254**	**514**	**49**
***Fusarium mexicanum* NRRL 53147**	**GCA 013396015.1**	**JAAOAM000000000.1**	**44**	**957**	**994**	**57**

Fusarium musae **F31**	**GCA_019915245.1**	**JAHBCI000000000.1**	**44**	**13**	**13**	**47**
Fusarium napiforme **NRRL 25196**	**GCA_013396005.1**	**JAAOAO000000000.1**	**42.1**	**1,411**	**1,421**	**56**
Fusarium proliferatum **ET1**	**GCA_900067095.1**	**FJOF00000000.1**	**45.2**	**32**	**221**	**65**
Fusarium pseudoanthophilum **NRRL 25211**	**GCA_013395995.1**	**JAAOAR000000000.1**	**42.8**	**1,816**	**1,864**	**59**
Fusarium pseudocircinatum **NRRL 36939**	**GCA_013396035.1**	**JAAOAS000000000.1**	**43.3**	**1,040**	**1,045**	**58**
Fusarium solani **SB1**	**GCA_023522795.1**	**JAMBZA000000000.1**	**59.4**	**/**	**19**	**45**
Fusarium tjaetaba **NRRL 66243**	**GCA_013396195.1**	**JAAQRI000000000.1**	**43.1**	**866**	**915**	**57**
Fusarium verticillioides **BRIP53263**	**GCA_003317015.2**	**QJUS00000000.1**	**42.4**	**153**	**931**	**54**
Glonium stellatum **CBS 207.34**	**GCA_001692915.1**	**LKAO00000000.1**	**40.5**	**2730**	**4423**	**48**
Halenospora varia **MPI-CAGE-AT-0135**	**GCA_020726485.1**	**JAHEWH000000000.1**	**55.6**	**/**	**108**	**79**
Hypoxylon cercidicola **CBS 119009**	**GCA_022578845.1**	**JAJLYK000000000.1**	**36.6**	**60**	**163**	**84**
Hypoxylon fuscum **CBS 119018**	**GCA_022578945.1**	**JAJLYN000000000.1**	**39**	**552**	**620**	**58***
Hypoxylon rubiginosum **MUCL52887**	**GCA_902806565.1**	**CADCXA000000000.1**	**48.3**	**/**	**70**	**63***
Icmadophila ericetorum **T1913**	**GCA_022814295.1**	**JALDZD000000000.1**	**36.9**	**836**	**932**	**80**
Immersiella caudata **CBS 606.72**	**GCA_030512335.1**	**JAULSU000000000.1**	**41.3**	**/**	**11**	**55**
Jackrogersella minutella **CBS 135445**	**GCA_022578535.1**	**JAJKKJ000000000.1**	**35.7**	**537**	**632**	**78**
Karstenula rhodostoma **CBS 690.94**	**GCA_010093485.1**	**JAAEJB000000000.1**	**45.1**	**139**	**669**	**64**
Knufia peltigerae **TK_35**	**GCA_030247855.1**	**JAPDRN000000000.1**	**39**	**737**	**827**	**30**
Lachnellula willkommii **CBS 172.35**	**GCA_007825375.1**	**QGML00000000.1**	**51**	**/**	**20,879**	**43**
Lecanicillium fungicola Babe33	GCA_027595875.1	JANJQO000000000.1	39	/	3,946	83
Lentithecium fluviatile **CBS 122367**	**GCA_010405425.1**	**JAAGLE000000000.1**	**54.7**	**195**	**2,078**	**73**
Lepraria neglecta **Allen 5258**	**GCA_033220425.1**	**JASNWA000000000.1**	**42**	**11**	**11**	**52**
Lizonia empirigonia **CBS 542.76**	**GCA_009982855.1**	**JAADKA000000000.1**	**51.5**	**/**	**423**	**42**
Lobaria immixta **Werth ST12-03b**	**GCA_022814215.1**	**JALDZH000000000.1**	**55.9**	**1,045**	**1,243**	**87**
Lojkania enalia **(formerly** ***Didymosphaeria enalia*****) CBS 304.66**	**GCA_010094045.1**	**JAAEIZ000000000.1**	**61.2**	**611**	**2,127**	**63**
Lophiotrema nucula **CBS 627.86**	**GCA_010015825.1**	**JAADKP000000000.1**	**48.6**	**187**	**286**	**78**
Lophium mytilinum **CBS 269.34**	**GCA_010093605.1**	**JAAEJF000000000.1**	**43.4**	**49**	**369**	**35**
Madurella fahalii **IFM 68171**	**GCA_045866475.1**	**BAAFSV000000000.1**	**40**	**6**	**6**	**54**
Madurella mycetomatis mm55	GCA_001275765.2	LCTW00000000.2	36.7	804	829	50
Melanomma pulvis-pyrius **CBS 109.77**	**GCA_010093585.1**	**JAAEJI000000000.1**	**42.1**	**1,754**	**1,771**	**51***
Monilinia fructicola **Mfrc123**	**GCA_008692225.1**	**VICG00000000.1**	**44**	**/**	**20**	**40**
Monilinia laxa **Mlax316**	**GCA_009299455.1**	**VIGI00000000.1**	**42.8**	**/**	**49**	**39**
Monilinia vaccinii-corymbosi **RL-1**	**GCA_017357885.1**		**30**	**9 chromosomes**		**30**
Monosporascus ibericus **CBS 110550**	**GCA_004154915.1**	**QJNU00000000.1**	**87.2**	**1,612**	**1,838**	**64**
Mytilinidion resinicola **CBS 304.34**	**GCA_010093595.1**	**JAAEJJ000000000.1**	**47.3**	**73**	**577**	**52**
Nannizzia gypsea **CBS 118893**	**GCA_000150975.2**	**ABQE00000000.1**	**23.2**	**18**	**317**	**46**
Nemania abortiva **FL1152**	**GCA_022578505.1**	**JAJKKU000000000.1**	**49.6**	**888**	**929**	**154**
Nemania bipapillata **CP14**	**GCA_029590615.1**	**JAPESX010000000**	**37.3**	**3747**	**3747**	**51**
Nemania diffusa **YAFEF818**	**GCA_038501195.1**	**JBBPUQ000000000.1**	**55.6**	**/**	**19**	**71***

***Nemania serpens* AZ0576**	**GCA_022578855.1**	**JAJKKN000000000.1**	**49.5**	**565**	**657**	**131**
***Neocucurbitaria cava* IMI 356814**	**GCA_027946455.1**	**JAPEUY000000000.1**	**34.8**	**/**	**24**	**29**
***Oidiodendron maius* Zn**	**GCA_000827325.1**	**JMDP00000000.1**	**46.4**	**100**	**433**	**85**
***Ophidiomyces ophidiicola* CBS 122913**	**GCA_022830035.1**	**JALAZP000000000.1**	**21.9**	**115**	**127**	**25**
***Parachaetomium inaequale* CBS 284.82**	**GCA_033296685.1**	**JASJSX000000000.1**	**36.8**	**999**	**1,213**	**41**
Paraconiothyrium brasiliense M42-189	GCA_022225985.2	JAKJXO000000000.2	39.3	86	108	56
***Paraphaeosphaeria sporulosa* AP3s5-JAC2a**	**GCA_001642045.1**	**LXPO00000000.1**	**38.5**	**61**	**445**	**75**
***Peltigera leucophlebia* HD1-Pleu1**	**GCA_022814155.1**	**JALDZK000000000.1**	**44.7**	**4464**	**4475**	**27**
Penicillium adametzioides	Genome not available					
***Penicillium angulare* IBT 27051**	**GCA_028827245.1**	**JAPZBL000000000.1**	**37.8**	**/**	**12**	**79**
***Penicillium arizonense* CBS 141311**	**GCA_001773325.1**	**LXJU00000000.1**	**33.7**	**396**	**548**	**62**
Penicillium camemberti FM 013	GCA_000513335.1	CBVV000000000.1	35	180	936	74
***Penicillium canescens* IBT 15451**	**GCA_028828765.1**	**JAQJZN000000000.1**	**46.3**	**/**	**15**	**79**
***Penicillium cataractarum* IBT 29864**	**GCA_028827025.1**	**JAPZBS000000000.1**	**37.4**	**/**	**11**	**53**
***Penicillium chermesinum* IBT 19713**	**GCA_028974085.1**	**JAPQKS000000000.1**	**27.6**	**/**	**10**	**45**
***Penicillium concentricum* IBT 3081**	**GCA_028827145.1**	**JAPZBT000000000.1**	**30**	**/**	**6**	**71**
***Penicillium expansum* MD-8**	**GCA_000769745.1**	**JQFZ00000000.1**	**32.4**	**382**	**382**	**73**
***Penicillium glandicola* 3C**	**GCA_902713505.1**	**CACTIM000000000.2**	**28.4**	**232**	**232**	**69**
***Penicillium griseofulvum* PG3**	**GCA_001561935.1**	**LHQR00000000.1**	**29.1**	**/**	**92**	**67**
***Penicillium hordei* IBT 12815**	**GCA_028827395.1**	**JAQJAE000000000.1**	**33.8**	**/**	**6**	**93**
Penicillium italicum PHI-1	GCA_000769765.1	JQGA00000000.1	30.2	/	1,632	51
***Penicillium longicatenatum* IBT 33135**	**GCA_028827895.1**	**JAQJAH000000000.1**	**32.5**	**/**	**7**	**69**
Penicillium nordicum DAOMC 185683	GCA_001278595.1	LHQQ00000000.1	30.8	996	1,042	78
Penicillium raciborskii	Genome not available					
***Penicillium rolfsii* F1880**	**GCA_011392555.1**	**QMFL00000000.1**	**32.4**	**27**	**165**	**58**
Penicillium steckii IBT 24891	GCA_002072375.1	MLKD00000000.1	32.1	84	331	51
***Penicillium thymicola* DAOM 180753**	**GCA_030142185.1**	**LACB00000000.1**	**33.9**	**2,167**	**2,182**	**82**
***Penicillium verrucosum* IBT 35672**	**GCA_028828655.1**	**JAQKAO000000000.1**	**32.8**	**/**	**8**	**80**
***Periconia digitata* CNCM I-4278**	**GCA_948474695.1**	**CAOQHR000000000.1**	**39**	**/**	**15**	**80**
***Periconia macrospinosa* DSE2036**	**GCA_003073855.1**	**PCYO00000000.1**	**55**	**1,566**	**2,976**	**122**
***Pestalotiopsis fici* W106-1**	**GCA_000516985.1**	**ARNU00000000.1**	**51.9**	**39**	**518**	**94**
***Phlyctema vagabunda* 19-DSS-EL-015**	**GCA_045999785.1**	**JBFCZG000000000.1**	**38.2**	**12**	**12**	**52**
***Phyllosticta citribraziliensis* CPC 17464**	**GCA_038025025.1**	**JBBPEH000000000.1**	**31.4**	**22**	**22**	**26**
***Phyllosticta citricarpa* CBS 122482**	**GCA_038025095.1**	**JBBPEG000000000.1**	**30**	**107**	**107**	**26**
***Phyllosticta paracitricarpa* CBS 141358**	**GCA_038051015.1**	**JBBPBF000000000.1**	**30.8**	**90**	**90**	**26**
***Physcia stellaris* C0375214F**	**GCA_018902385.1**	**JABSSW000000000.1**	**44**	**155**	**166**	**78**
***Plenodomus biglobosus* (formerly *Leptosphaeria biglobosa*) CA1**	**GCA_022343325.1**	**JACTNT000000000.1**	**32.6**	**/**	**59**	**36**

***Plenodomus lingam* (formerly *Leptosphaeria maulans*) CAN1**	**GCA_022343315.1**	**JACTNS000000000.1**	**42**	**48**	**49**	**35**
***Pleopsidium flavum* Spribille 41593/TS-e1965**	**GCA_026027315.1**	**JAPEUA000000000.1**	**30.8**	**/**	**4,571**	**32**
***Podospora australis* PSN309**	**GCA_033296905.1**	**JASWIB000000000.1**	**37.8**	**578**	**747**	**60**
***Polyplosphaeria fusca* CBS 125425**	**GCA_010093805.1**	**JAAEJQ000000000.1**	**37**	**468**	**625**	**57**
***Polytolypa hystricis* UAMH7299**	**GCA_002573605.1**	**PDNA00000000.1**	**34.7**	**641**	**658**	**44**
***Pseudovirgaria hyperparasitica* CBS 121739**	**GCA_010093815.1**	**JAAEJS000000000.1**	**35.4**	**122**	**543**	**53**
Purpureocillium lavendulum YMF1.00683	GCA_029168975.1	JAQHRD000000000.1	46.1	/	49	51*
***Purpureocillium lilacinum* CBS 284.36**	**GCA_023168085.2**	**BQKW00000000.1**	**38.6**	**/**	**10**	**49**
***Purpureocillium takamizusanense* PT3**	**GCA_022605165.1**		**35.6**	**14 chromosomes**		**14**
Pyricularia grisea* (formerly *Magnaporthe grise	**GCA_004355905.1**	**RRCK00000000.1**	**44.6**	**43**	**271**	**62***
Pyricularia oryzae MZ5-1-6	GCA_004346965.1	/	42.7	7 chromosomes		66
***Ramularia collo-cygni* URUG2**	**GCA_900074925.1**	**FJUY00000000.1**	**32.3**	**78**	**473**	**51**
***Rhinocladiella mackenziei* CBS 650.93**	**GCA_000835555.1**	**JYBU00000000.1**	**32.5**	**17**	**130**	**30**
***Sarocladium implicatum* TR**	**GCA_021176775.1**	**JAJEVK000000000.1**	**30.2**	**17**	**127**	**41**
***Scedosporium apiospermum* IHEM 14462**	**GCA_000732125.1**	**JOWA00000000.1**	**43.4**	**176**	**333**	**34**
***Sclerotinia borealis* F-4128**	**GCA_000503235.1**	**AYSA00000000.1**	**39.5**	**1,241**	**1,741**	**42**
***Sclerotinia sclerotiorum* 1980**	**GCA_000146945.2**	**AAGT00000000.1**	**38.3**	**36**	**679**	**48**
***Sclerotinia trifoliorum* SwB9**	**GCA_905066765.1**	**CAJHIA000000000.1**	**39.9**	**/**	**42**	**50**
***Sphaerosporella brunnea* Sb_GMNB300**	**GCA_008704415.1**	**VXIS00000000.1**	**51.6**	**872**	**872**	**19**
Sirodesmium diversum	Genome not available					
***Stachybotrys elegans* MPI-CAGE-CH-0235**	**GCA_020746835.1**	**JAGPNK000000000.1**	**43.5**	**/**	**73**	**84**
Sticta canariensis	**GCA_022814045.1**	**JALDZP000000000.1**	**43.4**	**3190**	**3190**	**36**
***Stromatinia cepivora* JRUF-117**	**GCA_014898415.1**	**RCTD00000000.1**	**55.7**	**/**	**48**	**57**
***Talaromyces islandicus* WF-38-12**	**GCA_000985935.1**	**CVMT00000000.1**	**34.7**	**116**	**1,345**	**72**
***Teloschistes chrysophthalmus* LIQ78TCHR-2**	**GCA_023646055.1**	**JALAIL000000000.1**	**27**	**776**	**776**	**48**
***Thelotrema lepadinum* Spribille 44606**	**GCA_022813995.1**	**JALDZR000000000.1**	**37.1**	**355**	**376**	**85**
***Thyridium curvatum* D216**	**GCA_004353045.1**	**SKBQ00000000.1**	**40.4**	**/**	**359**	**66**
***Trapelia coarctata* P141**	**GCA_022813945.1**	**JALDZT000000000.1**	**34**	**287**	**291**	**54**
***Trematosphaeria pertusa* CBS 122368**	**GCA_010094035.1**	**JAAEIY000000000.1**	**47.7**	**101**	**812**	**71**
Trichoderma arundinaceum	GCA_003012105.1	PXOA00000000.1	36.9	1,37	1,527	59
Trichoderma hypoxylon	Genome not available					
***Trichoderma simmonsii* GH-Sj1**	**GCA_019565615.1**	**/**	**40.1**	**7 chromosomes**		**45**
***Trichoderma virens* Gv29-8**	**GCA_000170995.2**	**ABDF00000000.2**	**39**	**93**	**94**	**65**
***Trichophyton benhamiae* CBS 112371**	**GCA_000151125.2**	**ABSU00000000.1**	**22.2**	**/**	**68**	**36**
***Trichophyton equinum* CBS 127.97**	**GCA_000151175.1**	**ABWI00000000.1**	**24.1**	**123**	**1714**	**35**
Trichophyton interdigitale UCMS-IGIB-CI12	GCA_012182645.1	JAATJQ000000000.1	22	818	1934	39
Trichophyton rubrum CMCC(F)T1i	GCA_001651445.1	LHPM00000000.1	22.3	19	257	35
***Trichophyton tonsurans* CBS 112818**	**GCA_000151455.1**	**ACPI00000000.1**	**23**	**110**	**1163**	**35**

***Trichophyton verrucosum* HKI 0517**	**GCA_000151505.1**	**ACYE00000000.1**	**22.5**	**523**	**612**	**38**
Trichophyton violaceum CMCC(F)T3l	GCA_001651435.1	LHPN00000000.1	23.4	261	938	34
***Truncatella angustata* MPI-SDFR-AT-0073**	**GCA_020726525.1**	**JAGPXC000000000.1**	**47.1**	**/**	**17**	**105**
***Usnochroma carphineum* LIQ69CCAR**	**GCA_023645975.1**	**JALAHV000000000.1**	**37**	**/**	**2385**	**59**
***Ustulina deusta* IL1129**	**GCA_023159155.1**	**JAJNNX000000000.1**	**56.1**	**/**	**125**	**111**
***Variospora aurantia* LIQ74CAUR**	**GCA_023645955.1**	**JALAHZ000000000.1**	**24.8**	**/**	**5327**	**23**
***Westerdykella ornata* CBS 379.55**	**GCA_010094085.1**	**JAAEJA000000000.1**	**27**	**94**	**208**	**41**
Xanthoria parietina	GCA_964263705.1	CAXVWH000000000.1	30	58	59	49
***Xylaria acuta* CBS 122032**	**GCA_022495125.1**	**JAJJWY000000000.1**	**47.2**	**761**	**1009**	**94**
***Xylaria arbuscula* CBS 124340**	**GCA_022385695.1**	**JAJLRG000000000.1**	**51.2**	**89**	**141**	**115**
***Xylaria bambusicola* CBS 139988**	**GCA_022495145.1**	**JAJJWZ000000000.1**	**45.7**	**233**	**377**	**107**
***Xylaria castorea* CBS 124033**	**GCA_022495115.1**	**JAJJXA000000000.1**	**45.9**	**461**	**528**	**96**
***Xylaria cf. heliscus* FL0509**	**GCA_022539335.1**	**JAJKLH000000000.1**	**44.9**	**/**	**72**	**98**
***Xylaria cubensis* CBS 116.85**	**GCA_022385715.1**	**JAJLRI000000000.1**	**44.8**	**333**	**384**	**120**
***Xylaria curta* CBS 114988**	**GCA_022495235.1**	**JAJJXH000000000.1**	**43.9**	**269**	**319**	**133**
***Xylaria digitata* CBS 161.22**	**GCA_022495195.1**	**JAJJXK000000000.1**	**58.4**	**888**	**949**	**111**
***Xylaria flabelliformis* G536**	**GCA_007182795.1**	**VFLP00000000.1**	**41.2**	**155**	**162**	**106**
Xylaria grammica IHI A82	GCA_004014815.1	RYZI00000000.1	47	1053	1058	119
Xylaria hypoxylon DSM 108379	GCA_004768795.1	SKBN00000000.1	42.8	635	637	104
***Xylaria intraflava* YMJ 725**	**GCA_022385655.1**	**JAJJJO000000000.1**	**35.9**	**438**	**577**	**72**
***Xylaria longipes* CBS 148.73**	**GCA_025201785.1**	**JAJLRH000000000.1**	**50.8**	**974**	**1054**	**124**
***Xylaria multiplex* DSM 110363**	**GCA_011057905.1**	**WUBL00000000.1**	**45.6**	**/**	**389**	**116**
***Xylaria palmicola* CBS 124036**	**GCA_022385595.1**	**JAJJJN000000000.1**	**43.9**	**548**	**809**	**92**
***Xylaria polymorpha* DSM 105756**	**GCA_003426235.1**	**NQIM00000000.1**	**43.5**	**947**	**951**	**98**
***Xylaria telfairii* CBS 121673**	**GCA_022495065.1**	**JAJJXI000000000.1**	**46.9**	**1027**	**1120**	**115**
***Xylographa soralifera* Spribille 41478**	**GCA_022813805.1**	**JALEAA000000000.1**	**38.1**	**412**	**738**	**71**
***Xylographa trunciseda* Spribille 41477**	**GCA_022813785.1**	**JALEAB000000000.1**	**26.8**	**513**	**1355**	**46**
***Zalerion maritima* ATCC 34329**	**GCA_022664585.2**	**JAKWBI000000000.2**	**58.4**	**2208**	**2240**	**21**
***Zopfia rhizophila* CBS 207.26**	**GCA_010093925.1**	**JAAEIT000000000.1**	**152.8**	**864**	**1441**	**95**
Zymoseptoria brevis Zb18110	GCA_000966595.1	LAFY00000000.1	31.9	/	6070	52
***Zymoseptoria tritici* (formerly *Mycosphaerella graminicola*) IPO323**	**GCA_000219625.1**	**ACPE00000000.1**	**39.7**	**21**	**24**	**73**

* anti-SMASH analysis performed using genomic.fna file
Reference genomes are indicated in bold font.

Supplementary Table S2: List of the NRPSs identified by antiSMASH analysis as having a single adenylation domain and two condensation domains, therefore possibly involved in the synthesis of homodipeptides

Protein accession number	Gene accession number	Species name	Domain organization	Substrate prediction by antiSMASH (by PARAS v1.0.1)	Metabolite prediction by anti-SMASH	PKS present in the BGC
KAF2014423-A	*BU24DRAFT_423380*	*Aaosphaeria arxii*	C1-A-PP-C2	Phe (Phe)	Aspirochlorine	
KAF2015070-A	*BU24DRAFT_423985*	*Aaosphaeria arxii*	PP-C1-A-PP-C2	Phe (Phe)	NP	
KAF2021067-A	*BU24DRAFT_457083*	*Aaosphaeria arxii*	A-PP-C1-PP-PP-C2	NP (HMMTyr)	Dimethylcoprogen	
MCJ1366360-A	*MMC16_005488*	*Acarospora aff. strigata*	C1-A-PP-C2	Phe (Phe)	NP	
MCJ1368281-A	*MMC16_007423*	*Acarospora aff. Strigata*	C1-A-PP-C2	Trp (Leu)	NP	
OAA76647-A	*LEL_06331*	*Akanthomyces lecanii*	C1-A-PP-C2	Phe (Phe)	Acetylaranotin	
OAA79903-A	*LEL_03389*	*Akanthomyces lecanii*	A-PP-C1-PP-C2	NP (AMHOrn)	Metachelin C / metachelin A / metachelin A-CE / metachelin B / dimerumic acid 11-mannoside / dimerumic acid	
KAJ4153649-A	*LMH87_010129*	*Akanthomyces muscarius*	C1-A-PP-C2	Phe (Phe)	Penigainamide A / penigainamide B / penigainamide C / adametizine A / FA2097 / outovirin A / outovirin C / pretrichodermamide C	
CAD6564771-A	*ASARMPREDX12_004428*	*Alectoria sarmentosa*	C1-A-PP-C2	Phe (Phe)	Acetylaranotin	
CAD6569118-A	*ASARMPREDX12_002207*	*Alectoria sarmentosa*	PP-C1-A-PP-C2	Phe (Phe)	Aspirochlorine	
CAD6593808-A	*ASARMPREDX12_007679*	*Alectoria sarmentosa*	A-PP-C1-PP-C2	NP (Ala)	NP	

KAF1994960-A	*P154DRAFT_446469*	*Amniculicola lignicola*	A-PP-C1-PP-PP-C2	NP (HMMTyr)	Metachelin C / metachelin A / metachelin A-CE / metachelin B / dimerumic acid 11-mannoside / dimerumic acid
KAF1997711-A	*P154DRAFT_622256*	*Amniculicola lignicola*	A-PP-C1-PP-C2	NP (AMHOrn)	NP
KAF1998231-A	*P154DRAFT_469622*	*Amniculicola lignicola*	C1-A-PP-C2	Phe (Phe)	Aspirochlorine
KAG9230922-A	*BJ875DRAFT_430746*	*Amylocarpus encephaloides*	PP-C1-A-PP-C2	Phe (Phe)	Aspirochlorine
KAG9237427-A	*BJ875DRAFT_157054*	*Amylocarpus encephaloides*	A-PP-C1-PP-C2	NP (AMHOrn)	Metachelin C / metachelin A / metachelin A-CE / metachelin B / dimerumic acid 11-mannoside / dimerumic acid
KAI0880199-A	*GGS22DRAFT_193548*	*Annulohypoxylon maeteangense*	C1-A-PP-C2	NP (PAB acid)	NP
KAI0880571-A	*GGS22DRAFT_174722*	*Annulohypoxylon maeteangense*	A-PP-C1-PP-C2	Ala (2SADD acid)	Dimethylcoprogen
KAI0881677-A	*GGS22DRAFT_191861*	*Annulohypoxylon maeteangense*	PP-C1-A-PP-C2	NP (Ala)	NP
KAI0886471-A	*GGS22DRAFT_113993*	*Annulohypoxylon maeteangense*	C1-A-PP-C2	Phe (Phe)	Penigainamide A / penigainamide B / penigainamide C / adametizine A / FA2097 / outovirin A / outovirin C / pretrichodermamide C
KAI0888102-A	*GGS22DRAFT_93277*	*Annulohypoxylon maeteangense*	PP-C1-A-PP-C2	Phe (Phe)	NP
KAI1452670-A	*F4805DRAFT_21430*	*Annulohypoxylon moriforme*	PP-C1-A-PP-C2	NP (Piperazic acid)	NP
KAI1453861-A	*F4805DRAFT_442042*	*Annulohypoxylon moriforme*	A-PP-C1-PP-C2	Ala (2SADD acid)	Dimethylcoprogen
KAI1457516-A	*F4805DRAFT_467352*	*Annulohypoxylon moriforme*	PP-C1-A-PP-C2	Phe (Phe)	Aspirochlorine

KAI1458188-A	*F4805DRAFT_150525*	*Annulohypoxylon moriforme*	PP-C1-A-PP-C2	Phe (Phe)	NP	
KAI1462852-A	*F4805DRAFT_452677*	*Annulohypoxylon moriforme*	PP-C1-A-PP-C2	NP (Ala)	NP	
KAI1447257-A	*F5Y02DRAFT_40425*	*Annulohypoxylon stygium*	PP-C1-A-PP-C2	NP (PAB acid)	NP	T1PKS with DH and KR domains
KAI1440009-A	*F5Y02DRAFT_424682*	*Annulohypoxylon stygium*	C1-A-PP-C2	Phe (Phe)	NP	
KAI1445839-A	*F5Y02DRAFT_426441*	*Annulohypoxylon stygium*	PP-C1-A-PP-C2	Phe (Phe)	NP	
KAI1442102-A	*F5Y02DRAFT_269459*	*Annulohypoxylon stygium*	A-PP-C1-PP-C2	Ala (2SADD acid)	Dimethylcoprogen	
KAI1441715-A	*F5Y02DRAFT_421789*	*Annulohypoxylon stygium*	PP-C1-A-PP-C2	NP (Ala)	NP	
KAI1204509-A	*F4807DRAFT_331715*	*Annulohypoxylon truncatum*	PP-C1-A-PP-C2	NP (NP)	NP	
KAI1206044-A	*F4807DRAFT_243549*	*Annulohypoxylon truncatum*	A-PP-C1-PP-C2	Ala (2SADD acid)	Dimethylcoprogen	
KAI1208210-A	*F4807DRAFT_156117*	*Annulohypoxylon truncatum*	C1-A-PP-C2	Phe (Phe)	NP	
CAJ2504437-A	*KHLLAP_LOCUS4905*	*Anthostomella pinea*	C1-A-PP-C2	Phe (Phe)	Aspirochlorine	
CAJ2510586-A	*KHLLAP_LOCUS11054*	*Anthostomella pinea*	PP-C1-A-PP-C2	NP (Piperazic acid)	NP	T1PKS with DH and KR domains
CAJ2512564-A	*KHLLAP_LOCUS13032*	*Anthostomella pinea*	A-PP-C1-PP-C2	NP (Trp)	Dimethylcoprogen	
KAK8856632-A	*PGQ11_012544*	*Apiospora arundinis*	PP-C1-A-PP-C2	Phe (Phe)	Aspirochlorine	
KAK8867648-A	*PGQ11_006226*	*Apiospora arundinis*	A-PP-C1-PP-C2	NP (Trp)	Dimethylcoprogen	
KAK7909115-A	*PG985_014993*	*Apiospora marii*	PP-C1-A-PP-C2	Phe (Phe)	Aspirochlorine	
KAK7920142-A	*PG985_008164*	*Apiospora marii*	PP-PP-C1-A-PP-C2	NP (NP)	NP	
KAK7926898-A	*PG985_003896*	*Apiospora marii*	A-PP-C1-PP-C2	NP (Trp)	Dimethylcoprogen	T1PKS with DH, ER and KR domains
KAF3483164-A	*GIQ15_02488*	*Arthroderma uncinatum*	PP-C1-A-PP-C2	Phe (Phe)	Aspirochlorine	
KAF3492395-A	*GIQ15_01912*	*Arthroderma uncinatum*	PP-C1-A-PP-C2	NP (Ser)	NP	

KAE8337064-A	*BDV24DRAFT_177930*	*Aspergillus arachidicola*	PP-C1-A-PP-C2	Phe (Phe)	Aspirochlorine
KAE8340592-A	*BDV24DRAFT_164325*	*Aspergillus arachidicola*	A-PP-C1-PP-C2	NP (AMHOrn)	Aspergillicin A / aspergillicin F / aspergillicin G
KAL4920172-A	*BDW62DRAFT_177794*	*Aspergillus aurantiobrunneus*	A-PP-C1-PP-C2	NP (AMHOrn)	Metachelin C / metachelin A / metachelin A-CE / metachelin B / dimerumic acid 11-mannoside / dimerumic acid
KAL4916103-A	*BDW62DRAFT_218856*	*Aspergillus aurantiobrunneus*	PP-C1-A-PP-C2	NP (Ala)	NP
KAL4915959-A	*BDW62DRAFT_203115*	*Aspergillus aurantiobrunneus*	C1-A-PP-C2	Ala (Ala)	NP
KAL4913035-A	*BDW62DRAFT_205920*	*Aspergillus aurantiobrunneus*	PP-C1-A-PP-C2	Phe (Phe)	Acetylaranotin
KAE8148003-A	*BDV25DRAFT_142208*	*Aspergillus avenaceus*	A-PP-C1-PP-C2	NP (AMHOrn)	Metachelin C / metachelin A / metachelin A-CE / metachelin B / dimerumic acid 11-mannoside / dimerumic acid
KAE8152948-A	*BDV25DRAFT_169719*	*Aspergillus avenaceus*	C1-A-PP-C2	Phe (Phe)	Aspirochlorine
KAE8153506-A	*BDV25DRAFT_136797*	*Aspergillus avenaceus*	A-PP-C1-PP-C2	NP (Trp)	NP
KAE8154162-A	*BDV25DRAFT_136089*	*Aspergillus avenaceus*	A-PP-C1-PP-C2	NP (AMHOrn)	Metachelin C / metachelin A / metachelin A-CE / metachelin B / dimerumic acid 11-mannoside / dimerumic acid
GCB20026-A	*AAWM_02911*	*Aspergillus awamori*	PP-C1-A-PP-C2	Phe (Phe)	NP
GCB24294-A	*AAWM_07179*	*Aspergillus awamori*	A-PP-C1-PP-C2	NP (AMHOrn)	NP
OJJ66405-A	*ASPBRDRAFT_69823*	*Aspergillus brasiliensis*	PP-C1-A-PP-C2	Phe (Phe)	NP
OJJ68492-A	*ASPBRDRAFT_132942*	*Aspergillus brasiliensis*	A-PP-C1-PP-C2	NP (AMHOrn)	NP

OJJ76279-A	*ASPBRDRAFT_664586*	*Aspergillus brasiliensis*	A-PP-C1-PP-C2	Trp (Trp)	Notoamide F / (+)-semivioxanthin / waikikiamide B / waikikiamide C/ notoamide A / waikikiamide A	
KAE8360869-A	*BDV27DRAFT_161245*	*Aspergillus caelatus*	PP-C1-A-C2	NP (Pro)	NP	
KAE8367002-A	*BDV27DRAFT_170270*	*Aspergillus caelatus*	C1-A-PP-C2	Phe (Phe)	Aspirochlorine	T1PKS with SAT, DH and cMT domains
KAE8368439-A	*BDV27DRAFT_141969*	*Aspergillus caelatus*	A-PP-C1-PP-C2	NP (Trp)	NP	
KAE8368915-A	*BDV27DRAFT_153549*	*Aspergillus caelatus*	A-PP-C1-PP-C2	NP (AMHOrn)	Aspergillicin A / aspergillicin F / aspergillicin G	
PKY00251-A	*P168DRAFT_276125*	*Aspergillus campestris*	A-PP-C1-PP-C2	NP (Trp)	NP	
PKY00819-A	*P168DRAFT_243112*	*Aspergillus campestris*	A-PP-C1-PP-C2	NP (AMHOrn)	Metachelin C / metachelin A / metachelin A-CE / metachelin B / dimerumic acid 11-mannoside / dimerumic acid	
PKY03682-A	*P168DRAFT_270112*	*Aspergillus campestris*	PP-C1-A-PP-C2	Phe (Phe)	NP	
PLB34829-A	*BDW47DRAFT_128742*	*Aspergillus candidus*	A-PP-C1-PP-C2	NP (AMHOrn)	Metachelin C / metachelin A / metachelin A-CE / metachelin B / dimerumic acid 11-mannoside / dimerumic acid	
PLB35929-A	*BDW47DRAFT_127670*	*Aspergillus candidus*	PP-C1-A-PP-C2	NP (Ala)	NP	
PLB42136-A	*BDW47DRAFT_121922*	*Aspergillus candidus*	PP-C1-A-PP-C2	Phe (Phe)	NP	
KAJ0418003-A	*BJY00DRAFT_315399*	*Aspergillus*	PP-C1-A-PP-C2	Phe (Phe)	Acetylaranotin	
KAJ0422881-A	*BJY00DRAFT_322373*	*Aspergillus*	A-PP-C1-PP-C2	NP (AMHOrn)	NP	

EAW12140-A	*ACLA_061000*	*Aspergillus clavatus*	A-PP-C1-PP-C2	NP (AMHOrn)	Metachelin C / metachelin A / metachelin A-CE / metachelin B / dimerumic acid 11-mannoside / dimerumic acid	
RAK88632-A	*BO79DRAFT_237808*	*Aspergillus*	A-PP-C1-PP-C2	NP (AMHOrn)	NP	
RAK90455-A	*BO79DRAFT_262106*	*Aspergillus*	PP-C1-A-PP-C2	NP (Phe)	NP	
RAK93182-A	*BO79DRAFT_272577*	*Aspergillus costaricaensis*	A-PP-C1-PP-C2	NP (Trp)	Notoamide F / (+)-semivioxanthin / waikikiamide B / waikikiamide C/ notoamide A / waikikiamide A	
ODM18799-A	*SI65_05416*	*Aspergillus cristatus*	C1-A-PP-C2	Phe (Phe)	NP	
PWY61763-A	*BO83DRAFT_442433*	*Aspergillus eucalypticola*	A-PP-C1-PP-C2	NP (AMHOrn)	NP	
PWY68727-A	*BO83DRAFT_448773*	*Aspergillus eucalypticola*	PP-C1-A-PP-C2	Phe (Phe)	NP	
KAF7176351-A	*CNMCM7691_002276*	*Aspergillus felis*	PP-C1-A-PP-C2	Phe (Phe)	Aspirochlorine	
KAF7180871-A	*CNMCM7691_010162*	*Aspergillus felis*	C1-A-PP-C2	Phe (Phe)	Acetylaranotin	T1PKS with SAT, PT and TE domains
KAF7183064-A	*CNMCM7691_002899*	*Aspergillus felis*	A-PP-C1-PP-C2	NP (AMHOrn)	Metachelin C / metachelin A / metachelin A-CE / metachelin B / dimerumic acid 11-mannoside / dimerumic acid	
QMW25917-A	*G4B84_001162*	*Aspergillus flavus*	PP-C1-A-PP-C2	NP (Leu)	NP	
QMW32911-A	*G4B84_008342*	*Aspergillus flavus*	A-PP-C1-PP-C2	NP (Trp)	NP	
QMW34743-A	*G4B84_010209*	*Aspergillus flavus*	PP-C1-A-PP-C2	Phe (Phe)	Aspirochlorine	
QMW35998-A	*G4B84_011527*	*Aspergillus flavus*	A-PP-C1-PP-C2	NP (AMHOrn)	Aspergillicin A / aspergillicin F / aspergillicin G	

KAL5356154-A	*BJX96DRAFT_171343*	*Aspergillus floccosus*	A-PP-C1-PP-C2	NP (AMHOrn)	Metachelin C / metachelin A / metachelin A-CE / metachelin B / dimerumic acid 11-mannoside / dimerumic acid
KAL5356257-A	*BJX96DRAFT_171445*	*Aspergillus floccosus*	A-PP-C1-PP-C2	NP (AmHOrn)	Metachelin C / metachelin A / metachelin A-CE / metachelin B / dimerumic acid 11-mannoside / dimerumic acid
KAL5356675-A	*BJX96DRAFT_187398*	*Aspergillus floccosus*	PP-C1-A-PP-C2	Phe (Phe)	Acetylaranotin
KAL5364136-A	*BJX96DRAFT_187567*	*Aspergillus floccosus*	PP-C1-A-PP-C2	Phe (Phe)	Penigainamide A / penigainamide B / penigainamide C / adametizine A / FA2097 / outovirin A / outovirin C / pretrichodermamide C
KAL4760153-A	*BDW70DRAFT_160984*	*Aspergillus foveolatus*	A-PP-C1-PP-C2	NP (AMHOrn)	Metachelin C / metachelin A / metachelin A-CE / metachelin B / dimerumic acid 11-mannoside / dimerumic acid
KAL4756559-A	*BDW70DRAFT_165893*	*Aspergillus foveolatus*	PP-C1-A-PP-C2	Phe (Phe)	Aspirochlorine
KAF4230492-A	*CNMCM6805_000684*	*Aspergillus fumigatiaffinis*	Ech-A-PP-C1-PP-C2	NP (AMHOrn)	Metachelin C / metachelin A / metachelin A-CE / metachelin B / dimerumic acid 11-mannoside / dimerumic acid
KAF4234151-A	*CNMCM6805_008857*	*Aspergillus*	AmT-PP-C1-A-PP-	Phe (Phe)	Acetylaranotin
KAF4241475-A	*CNMCM6805_004133*	*Aspergillus*	PP-C1-A-PP-C2	Phe (Phe)	Aspirochlorine
KAL3493162-A	*BJX62DRAFT_224020*	*Aspergillus germanicus*	A-PP-C1-C2	Trp (Trp)	N-Acetyltryptophan
KAL3484364-A	*BJX62DRAFT_243995*	*Aspergillus germanicus*	PP-C1-A-PP-C2	Phe (Phe)	Acetylaranotin
KAL3496015-A	*BJX62DRAFT_252400*	*Aspergillus germanicus*	A-PP-C1-PP-C2	NP (AMHOrn)	NP

KAF7115455-A	*CNMCM5793_002413*	*Aspergillus hiratsukae*	A-PP-C1-PP-C2	NP (Trp)	NP
KAF7117609-A	*CNMCM5793_006701*	*Aspergillus hiratsukae*	PP-E-C1-A-PP-C2	NP (Pro)	NP
RAL15825-A	*BO97DRAFT_467997*	*Aspergillus homomorphus*	A-PP-C1-PP-C2	NP (AMHOrn)	Dimethylcoprogen
RAL17382-A	*BO97DRAFT_447748*	*Aspergillus homomorphus*	A-PP-C1-PP-PP-C2	NP (AMHOrn*)	NP
KAL4886428-A	*BJY04DRAFT_213196*	*Aspergillus karnatakaensis*	PP-C1-A-PP-C2	Phe (Phe)	Acetylaranotin
KAL4880999-A	*BJY04DRAFT_218520*	*Aspergillus karnatakaensis*	A-PP-C1-C2	Trp (Trp)	N-Acetyltryptophan
KAL4879115-A	*BJY04DRAFT_229461*	*Aspergillus karnatakaensis*	A-PP-C1-PP-C2	NP (AMHOrn)	NP
BCR96254-A	*AKAW2_21194S*	*Aspergillus luchuensis*	A-PP-C1-PP-C2	Trp (Trp)	Notoamide F / (+)-semivioxanthin / waikikiamide B / waikikiamide C/ notoamide A / waikikiamide A
BCR98364-A	*AKAW2_40047A*	*Aspergillus luchuensis*	PP-C1-A-PP-C2	Phe (Phe)	NP
BCS00409-A	*AKAW2_50750A*	*Aspergillus luchuensis*	A-PP-C1-PP-C2	NP (AMHOrn)	NP
BCS04213-A	*AKAW2_80014A*	*Aspergillus luchuensis*	PP-PP-C1-A-nMT-PP-C2	Phe (Ala)	NP
KAB8268027-A	*BDV30DRAFT_243780*	*Aspergillus minisclerotigenes*	A-PP-C1-PP-C2	NP (AMHOrn)	NP
KAB8276351-A	*BDV30DRAFT_235805*	*Aspergillus minisclerotigenes*	PP-C1-A-PP-C2	Pro (Ala)	NP
KAL6235270-A	*BDW75DRAFT_230453*	*Aspergillus navahoensis*	C1-A-PP-C2	Phe (Phe)	Acetylaranotin
KAL6235216-A	*BDW75DRAFT_240384*	*Aspergillus navahoensis*	A-PP-C1-PP-C2	NP (AMHOrn)	Metachelin C / metachelin A / metachelin A-CE / metachelin B / dimerumic acid 11-mannoside / dimerumic acid

KAL6232020-A	*BDW75DRAFT_243380*	*Aspergillus navahoensis*	PP-C1-A-PP-C2	NP (Ala)	NP	T1PKS with DH, cMT and KR domains
PYH29882-A	*BO87DRAFT_410144*	*Aspergillus neoniger*	PP-C1-A-PP-C2	Phe (Phe)	NP	
PYH30406-A	*BO87DRAFT_418949*	*Aspergillus neoniger*	A-PP-C1-PP-C2	NP (AMHOrn)	NP	
PYH36058-A	*BO87DRAFT_414615*	*Aspergillus neoniger*	A-PP-C1-PP-C2	Trp (Trp)	Notoamide F / (+)-semivioxanthin / waikikiamide B / waikikiamide C/ notoamide A / waikikiamide A	
KAL4777461-A	*BDW60DRAFT_202357*	*Aspergillus nidulans* var. *acristatus*	A-PP-C1-PP-C2	NP (AMHOrn)	Metachelin C / metachelin A / metachelin A-CE / metachelin B / dimerumic acid 11-mannoside / dimerumic acid	
KAL4767480-A	*BDW60DRAFT_226532*	*Aspergillus nidulans* var. *acristatus*	PP-C1-A-PP-C2	Phe (Phe)	Aspirochlorine	
PYH52902-A	*BO96DRAFT_482873*	*Aspergillus niger (Aspergillus lacticoffeatus)*	PP-C1-A-PP-C2	Phe (Phe)	NP	
PYH58350-A	*BO96DRAFT_491931*	*Aspergillus niger (Aspergillus lacticoffeatus)*	A-PP-C1-PP-C2	NP (AMHOrn)	NP	
PYH62947-A	*BO96DRAFT_473278*	*Aspergillus niger (Aspergillus lacticoffeatus)*	A-PP-C1-PP-C2	Trp (Trp)	Notoamide F / (+)-semivioxanthin / waikikiamide B / waikikiamide C/ notoamide A / waikikiamide A	

PKX88429-A	*P174DRAFT_380384*	*Aspergillus novofumigatus*	PP-C1-A-PP-C2	Phe (Phe)	Penigainamide A / penigainamide B / penigainamide C / adametizine A / FA2097 / outovirin A / outovirin C / pretrichodermamide C
PKX89086-A	*P174DRAFT_425623*	*Aspergillus novofumigatus*	A-C1-PP-C2	NP (Trp)	NP
PKX91457-A	*P174DRAFT_505534*	*Aspergillus novofumigatus*	A-PP-C1-PP-C2	NP (AMHOrn)	Metachelin C / metachelin A / metachelin A-CE / metachelin B / dimerumic acid 11-mannoside / dimerumic acid
PKX97582-A	*P174DRAFT_449186*	*Aspergillus novofumigatus*	PP-C1-A-PP-C2	NP (His)	NP
KAB8217278-A	*BDV33DRAFT_206547*	*Aspergillus novoparasiticus*	A-PP-C1-PP-C2	NP (AMHOrn)	Aspergillicin A / aspergillicin F / aspergillicin G
KAB8224445-A	*BDV33DRAFT_199479*	*Aspergillus novoparasiticus*	PP-C1-A-PP-C2	Phe (Phe)	Aspirochlorine
BAE55983-A	*AO090005000993*	*Aspergillus oryzae*	PP-C1-A-PP-C2	NP (Leu)	NP
BAE56606-A	*AO090001000043*	*Aspergillus oryzae*	PP-C1-A-PP-C2	Phe (Phe)	Aspirochlorine
BAE62663-A	*AO090120000024*	*Aspergillus oryzae*	A-PP-C1-PP-C2	NP (Trp)	NP
BAE65630-A	*AO090103000167*	*Aspergillus oryzae*	A-PP-C1-PP-C2	NP (AMHOrn)	Metachelin C / metachelin A / metachelin A-CE / metachelin B / dimerumic acid 11-mannoside / dimerumic acid
KJK66624-A	*P875_00128042*	*Aspergillus parasiticus*	PP-C1-A-PP-C2	Phe (Phe)	Aspirochlorine
RDK39207-A	*M752DRAFT_278670*	*Aspergillus phoenicis*	A-PP-C1-PP-C2	NP (AMHOrn)	NP
RDK41674-A	*M752DRAFT_302173*	*Aspergillus phoenicis*	PP-C1-A-PP-C2	Phe (Phe)	NP

RDK41754-A	*M752DRAFT_300980*	*Aspergillus phoenicis*	A-PP-C1-PP-C2	Trp (Trp)	Notoamide F / (+)-semivioxanthin / waikikiamide B / waikikiamide C/ notoamide A / waikikiamide A	
RAH52064-A	*BO85DRAFT_408578*	*Aspergillus piperis*	A-PP-C1-PP-C2	NP (AMHOrn)	NP	
RAH54362-A	*BO85DRAFT_379097*	*Aspergillus piperis*	PP-C1-A-PP-C2	Phe (Phe)	NP	
RAH59955-A	*BO85DRAFT_518702*	*Aspergillus piperis*	A-PP-C1-PP-C2	Trp (4SDHAP acid)	Notoamide F / (+)-semivioxanthin / waikikiamide B / waikikiamide C/ notoamide A / waikikiamide A	
KAE8416764-A	*BDV36DRAFT_309927*	*Aspergillus pseudocaelatus*	C1-A-PP-C2	Phe (Phe)	Aspirochlorine	T1PKS with SAT, DH and cMT domains
KAE8417931-A	*BDV36DRAFT_309038*	*Aspergillus pseudocaelatus*	A-PP-C1-PP-C2	NP (Trp)	NP	
KAE8135387-A	*BDV38DRAFT_294703*	*Aspergillus pseudotamarii*	A-PP-C1-PP-C2	NP (Trp)	NP	
KAE8137495-A	*BDV38DRAFT_292943*	*Aspergillus pseudotamarii*	C1-A-PP-C2	Phe (Phe)	Aspirochlorine	T1PKS with SAT, DH and cMT domains
KAE8141335-A	*BDV38DRAFT_279237*	*Aspergillus pseudotamarii*	A-PP-C1-PP-C2	NP (AMHOrn)	NP	
KAL2851960-A	*BJY01DRAFT_232726*	*Aspergillus pseudoustus*	A-PP-C1-PP-C2	NP (AMHOrn)	NP	
KAL2842714-A	*BJY01DRAFT_248979*	*Aspergillus pseudoustus*	PP-C1-A-PP-C2	Phe (Phe)	Acetylaranotin	
GIJ86440-A	*Asppvi_005329*	*Aspergillus pseudoviridinutans*	PP-C1-A-PP-C2	Phe (Phe)	Acetylaranotin	Two T1PKS with SAT, PT and TE domains

GIJ87087-A	*Asppvi_005990*	*Aspergillus pseudoviridinutans*	A-PP-C1-PP-C2	NP (AMHOrn)	NP
GIJ88184-A	*Asppvi_007102*	*Aspergillus pseudoviridinutans*	PP-C1-A-PP-C2	Phe (Phe)	Aspirochlorine
BCS25380-A	*APUU_50091A*	*Aspergillus*	PP-C1-A-PP-C2	NP (Ala)	NP
BCS26540-A	*APUU_51251A*	*Aspergillus*	A-PP-C1-PP-C2	NP (AMHOrn)	NP
BCS28409-A	*APUU_61457S*	*Aspergillus*	PP-C1-A-PP-C2	Phe (Phe)	Acetylaranotin
RJE23318-A	*PHISCL_04348*	*Aspergillus sclerotialis*	A-PP-C1-PP-C2	NP (AMHOrn)	Metachelin C / metachelin A / metachelin A-CE / metachelin B / dimerumic acid 11-mannoside / dimerumic acid
RJE25041-A	*PHISCL_02634*	*Aspergillus sclerotialis*	PP-C1-A-PP-C2	Phe (Phe)	NP
RJE25399-A	*PHISCL_02290*	*Aspergillus sclerotialis*	PP-C1-A-PP-C2	Phe (Phe)	NP
PYI03216-A	*BO78DRAFT_452037*	*Aspergillus sclerotiicarbonarius*	C1-A-PP-C2	Phe (Phe)	NP
PYI03599-A	*BO78DRAFT_451681*	*Aspergillus sclerotiicarbonarius*	A-PP-C1-PP-C2	NP (AMHOrn)	NP
KAE8327714-A	*BDV39DRAFT_204731*	*Aspergillus sergii*	A-PP-C1-PP-C2	NP (Trp)	NP
KAE8327816-A	*BDV39DRAFT_204530*	*Aspergillus sergii*	PP-C1-A-PP-C2	Phe (Phe)	Aspirochlorine
KAE8330591-A	*BDV39DRAFT_201999*	*Aspergillus sergii*	A-PP-C1-PP-C2	NP (AMHOrn)	NP
KAL4741677-A	*BDV11DRAFT_168043*	*Aspergillus similis*	PP-C1-A-PP-C2	Phe (Phe)	Acetylaranotin
KAL4741514-A	*BDV11DRAFT_168099*	*Aspergillus similis*	A-PP-C1-PP-C2	NP (AMHOrn)	Metachelin C / metachelin A / metachelin A-CE / metachelin B / dimerumic acid 11-mannoside / dimerumic acid
KAL4739819-A	*BDV11DRAFT_169733*	*Aspergillus similis*	A-PP-C1-C2	Trp (Trp)	N-Acetyltryptophan
KAL4735231-A	*BDV11DRAFT_212182*	*Aspergillus similis*	PP-C1-A-PP-C2	NP (Ala)	NP
PLN76560-A	*BDW42DRAFT_197087*	*Aspergillus*	PP-C1-A-PP-C2	Phe (Phe)	NP
PLN80181-A	*BDW42DRAFT_201350*	*Aspergillus*	A-PP-C1-PP-C2	Trp (NP)	NP

PLN84696-A	*BDW42DRAFT_199198*	*Aspergillus taichungensis*	A-PP-C1-PP-C2	NP (AMHOrn)	Metachelin C / metachelin A / metachelin A-CE / metachelin B / dimerumic acid 11-mannoside / dimerumic acid
KAE8160437-A	*BDV40DRAFT_302251*	*Aspergillus tamarii*	A-PP-C1-PP-C2	NP (AMHOrn)	Metachelin C / metachelin A / metachelin A-CE / metachelin B / dimerumic acid 11-mannoside / dimerumic acid
KAE8163394-A	*BDV40DRAFT_311763*	*Aspergillus tamarii*	C1-A-PP-C2	Phe (Phe)	Aspirochlorine
KAA8643828-A	*ATNIH1004_010603*	*Aspergillus tanneri*	A-PP-C1-PP-C2	NP (AMHOrn)	Metachelin C / metachelin A / metachelin A-CE / metachelin B / dimerumic acid 11-mannoside / dimerumic acid
KAA8644653-A	*ATNIH1004_008859*	*Aspergillus tanneri*	C1-A-PP-C2	Leu (Pro)	NP
KAA8645290-A	*ATNIH1004_006709*	*Aspergillus tanneri*	PP-C1-A-PP-C2	Phe (Phe)	Acetylaranotin
KAA8647877-A	*ATNIH1004_006579*	*Aspergillus tanneri*	PP-C1-A-PP-C2	Phe (Phe)	NP
KAA8648042-A	*ATNIH1004_003925*	*Aspergillus tanneri*	PP-C1-A-PP-C2	NP (Leu)	Choline
KAA8652098-A	*ATNIH1004_001002*	*Aspergillus tanneri*	A-PP-C1-PP-C2	NP (Trp)	Nidulanin A
EAU31750-A	*ATEG_07488*	*Aspergillus terreus*	A-PP-C1-PP-C2	NP (AMHOrn)	Metachelin C / metachelin A / metachelin A-CE / metachelin B / dimerumic acid 11-mannoside / dimerumic acid
EAU33874-A	*ATEG_06113*	*Aspergillus terreus*	PP-E-C1-A-PP-C2	Val (Val)	NP
EAU34769-A	*ATEG_04322*	*Aspergillus terreus*	C1-A-PP-C2	NP (Ala)	NP
EAU36744-A	*ATEG_03470*	*Aspergillus terreus*	C1-A-PP-C2	Phe (Phe)	Acetylaranotin
KAL4754638-A	*BDW72DRAFT_189935*	*Aspergillus terricola* var. *indicus*	A-PP-C1-PP-C2	NP (AMHOrn)	Metachelin C / metachelin A / metachelin A-CE / metachelin B / dimerumic acid 11-mannoside / dimerumic acid

KAL4751559-A	*BDW72DRAFT_192753*	*Aspergillus terricola* var. *indicus*	A-PP-C1-C2	Trp (Trp)	N-Acetyltryptophan
KAL4744987-A	*BDW72DRAFT_208574*	*Aspergillus terricola* var. *indicus*	PP-C1-A-PP-C2	NP (Ala)	NP
KAL4744360-A	*BDW72DRAFT_199672*	*Aspergillus terricola* var. *indicus*	PP-C1-A-PP-C2	Phe (Phe)	NP
KAL3439431-A	*BDV09DRAFT_191000*	*Aspergillus tetrazonus*	A-PP-C1-PP-C2	NP (AMHOrn)	Metachelin C / metachelin A / metachelin A-CE / metachelin B / dimerumic acid 11-mannoside / dimerumic acid
KAL3432252-A	*BDV09DRAFT_187705*	*Aspergillus tetrazonus*	C1-A-PP-C2	Phe (Phe)	Acetylaranotin
RHZ46026-A	*CDV56_102363*	*Aspergillus thermomutatus*	PP-C1-A-PP-C2	Phe (Phe)	Acetylaranotin
RHZ55542-A	*CDV56_103361*	*Aspergillus thermomutatus*	PP-C1-A-PP-C2	Phe (Phe)	Penigainamide A / penigainamide B / penigainamide C / adametizine A / FA2097 / outovirin A / outovirin C / pretrichodermamide C
RHZ65485-A	*CDV56_103512*	*Aspergillus thermomutatus*	A-PP-C1-PP-C2	NP (AMHOrn)	Metachelin C / metachelin A / metachelin A-CE / metachelin B / dimerumic acid 11-mannoside / dimerumic acid
KAE8309918-A	*BDV41DRAFT_590794*	*Aspergillus transmontanensis*	PP-C1-A-PP-C2	Phe (Phe)	Aspirochlorine
KAE8310171-A	*BDV41DRAFT_566806*	*Aspergillus transmontanensis*	A-PP-C1-PP-C2	NP (AMHOrn)	Aspergillicin A / aspergillicin F / aspergillicin G
KAE8311551-A	*BDV41DRAFT_578444*	*Aspergillus transmontanensis*	A-PP-C1-PP-C2	NP (Trp)	NP

GFN16130-A	*AtWU_05931*	*Aspergillus tubingensis*	A-PP-C1-PP-C2	NP (Trp)	Notoamide F / (+)-semivioxanthin / waikikiamide B / waikikiamide C/ notoamide A / waikikiamide A
GFN20808-A	*AtWU_10615*	*Aspergillus tubingensis*	A-PP-C1-PP-C2	NP (AMHOrn)	NP
GFN21647-A	*AtWU_11456*	*Aspergillus tubingensis*	PP-C1-A-PP-C2	Phe (Phe)	NP
RHZ48763-A	*CDV55_101528*	*Aspergillus turcosus*	A-PP-C1-PP-C2	NP (Trp)	NP
GIC86392-A	*Aud_002763*	*Aspergillus udagawae*	A-PP-C1-PP-C2	NP (AMHOrn)	Metachelin C / metachelin A / metachelin A-CE / metachelin B / dimerumic acid 11-mannoside / dimerumic acid
GIC87488-A	*Aud_003872*	*Aspergillus udagawae*	PP-C1-A-PP-C2	Phe (Phe)	Penigainamide A / penigainamide B / penigainamide C / adametizine A / FA2097 / outovirin A / outovirin C / pretrichodermamide C
KIA75386-A	*HK57_00105*	*Aspergillus ustus*	PP-C1-A-PP-C2	Phe (Phe)	Acetylaranotin
KIA75496-A	*HK57_00018*	*Aspergillus ustus*	A-PP-C1-C2	Trp (Trp)	N-Acetyltryptophan
KIA75527-A	*HK57_00716*	*Aspergillus ustus*	A-PP-C1-PP-C2	NP (AMHOrn)	NP
PYH65841-A	*BO88DRAFT_470052*	*Aspergillus vadensis*	PP-C1-A-PP-C2	Phe (Phe)	NP
PYH68690-A	*BO88DRAFT_63428*	*Aspergillus vadensis*	A-PP-C1-PP-C2	NP (AMHOrn)	NP
PYH74752-A	*BO88DRAFT_460400*	*Aspergillus vadensis*	A-PP-C1-PP-C2	Trp (Trp)	Notoamide F / (+)-semivioxanthin / waikikiamide B / waikikiamide C/ notoamide A / waikikiamide A
KAL4783354-A	*BJX76DRAFT_368477*	*Aspergillus varians*	PP-C1-A-PP-C2	Phe (Phe)	Acetylaranotin

KAL4781623-A	*BJX76DRAFT_11798*	*Aspergillus varians*	A-PP-C1-PP-C2	NP (AMHOrn)	Metachelin C / metachelin A / metachelin A-CE / metachelin B / dimerumic acid 11-mannoside / dimerumic acid
GIK07511-A	*Aspvir_003177*	*Aspergillus viridinutans*	PP-C1-A-PP-C2	Phe (Phe)	Penigainamide A / penigainamide B / penigainamide C / adametizine A / FA2097 / outovirin A / outovirin C / pretrichodermamide C
RDH29534-A	*BDQ94DRAFT_173806*	*Aspergillus welwitschiae*	A-PP-C1-PP-C2	NP (AMHOrn)	NP
RDH32322-A	*BDQ94DRAFT_171089*	*Aspergillus welwitschiae*	PP-C1-A-PP-C2	Phe (Phe)	NP
RDH36767-A	*BDQ94DRAFT_157614*	*Aspergillus welwitschiae*	PP-A-PP-C1-nMT-	NP (Leu)	NP
EMD58405-A	*COCSADRAFT_350779*	*Bipolaris sorokiniana*	C1-A-PP-C2	NP (Phe)	Sporidesmin
EMD64880-A	*COCSADRAFT_25865*	*Bipolaris sorokiniana*	C1-A-PP-C2	NP (Pro)	NP
EMD68320-A	*COCSADRAFT_156762*	*Bipolaris sorokiniana*	A-PP-C1-PP-PP-C2	NP (HMMTyr)	Metachelin C / metachelin A / metachelin A-CE / metachelin B / dimerumic acid 11-mannoside / dimerumic acid
TEY30136-A	*BOTCAL_0894g00010*	*Botryotinia calthae*	PP-C1-A-PP-C2	Phe (Phe)	NP
TEY35935-A	*BOTCAL_0573g00010*	*Botryotinia calthae*	PP-C1-A-PP-C2	Phe (Phe)	NP
TGO46046-A	*BCON_0348g00080*	*Botryotinia convoluta*	PP-C1-A-PP-C2	Phe (Phe)	NP
TGO58604-A	*BCON_0053g00390*	*Botryotinia convoluta*	A-PP-C1-PP-C2	NP (AMHOrn)	NP
KAF7886174-A	*EAF00_010277*	*Botryotinia globosa*	PP-C1-A-PP-C2	Phe (Phe)	NP
KAF7906074-A	*EAF00_000353*	*Botryotinia globosa*	A-PP-C1-PP-C2	NP (AMHOrn)	Metachelin C / metachelin A / metachelin A-CE / metachelin B / dimerumic acid 11-mannoside / dimerumic acid

TGO57096-A	*BOTNAR_0208g00080*	*Botryotinia narcissicola*	A-PP-C1-PP-C2	NP (AMHOrn)	Metachelin C / metachelin A / metachelin A-CE / metachelin B / dimerumic acid 11-mannoside / dimerumic acid
TGO62446-A	*BOTNAR_0113g00090*	*Botryotinia narcissicola*	PP-C1-A-PP-C2	Phe (Phe)	NP
KAF7947889-A	*EAE96_008965*	*Botrytis aclada*	PP-C1-A-PP-C2	Phe (Phe)	NP
KAF7958495-A	*EAE96_002038*	*Botrytis aclada*	A-PP-C1-PP-C2	NP (AMHOrn)	Metachelin C / metachelin A / metachelin A-CE / metachelin B / dimerumic acid 11-mannoside / dimerumic acid
KAF7937730-A	*EAE97_007526*	*Botrytis byssoidea*	A-PP-C1-PP-C2	NP (AMHOrn)	Metachelin C / metachelin A / metachelin A-CE / metachelin B / dimerumic acid 11-mannoside / dimerumic acid
KAF7946051-A	*EAE97_005089*	*Botrytis byssoidea*	PP-C1-A-PP-C2	Phe (Phe)	NP
EMR86853-A	*BcDW1_4492*	*Botrytis cinerea*	PP-C1-A-PP-C2	Phe (Phe)	NP
EMR90612-A	*BcDW1_747*	*Botrytis cinerea*	A-PP-C1-PP-C2	NP (AMHOrn)	Metachelin C / metachelin A / metachelin A-CE / metachelin B / dimerumic acid 11-mannoside / dimerumic acid
KAF7919517-A	*EAE98_009357*	*Botrytis deweyae*	A-PP-C1-PP-C2	NP (AMHOrn)	Metachelin C / metachelin A / metachelin A-CE / metachelin B / dimerumic acid 11-mannoside / dimerumic acid
KAF7925494-A	*EAE98_006719*	*Botrytis deweyae*	PP-C1-A-PP-C2	Phe (Phe)	NP

KAF7915766-A	*EAE99_010017*	*Botrytis elliptica*	A-PP-C1-PP-C2	NP (AMHOrn)	Metachelin C / metachelin A / metachelin A-CE / metachelin B / dimerumic acid 11-mannoside / dimerumic acid
KAF7922661-A	*EAE99_007238*	*Botrytis elliptica*	PP-C1-A-PP-C2	Phe (Phe)	NP
KAF5872659-A	*Bfra_006021*	*Botrytis fragariae*	PP-C1-A-PP-C2	Phe (Phe)	NP
KAF5878648-A	*Bfra_000815*	*Botrytis fragariae*	A-PP-C1-PP-C2	NP (AMHOrn)	Metachelin C / metachelin A / metachelin A-CE / metachelin B / dimerumic acid 11-mannoside / dimerumic acid
THV46312-A	*BGAL_0396g00050*	*Botrytis galanthina*	PP-C1-A-PP-C2	Phe (Phe)	NP
THV48601-A	*BGAL_0238g00020*	*Botrytis galanthina*	A-PP-C1-PP-C2	NP (AMHOrn)	NP
TGO38806-A	*BHYA_0067g00370*	*Botrytis hyacinthi*	A-PP-C1-PP-C2	NP (AMHOrn)	NP
TGO38936-A	*BHYA_0064g00320*	*Botrytis hyacinthi*	PP-C1-A-PP-C2	Phe (Phe)	NP
TGO21575-A	*BPAE_0212g00170*	*Botrytis paeoniae*	PP-C1-A-PP-C2	Phe (Phe)	NP
TGO28991-A	*BPAE_0020g00360*	*Botrytis paeoniae*	A-PP-C1-PP-C2	NP (AMHOrn)	Metachelin C / metachelin A / metachelin A-CE / metachelin B / dimerumic acid 11-mannoside / dimerumic acid
KAF7903301-A	*EAF01_006350*	*Botrytis porri*	PP-C1-A-PP-C2	Phe (Phe)	NP
KAF7913783-A	*EAF01_000189*	*Botrytis porri*	A-PP-C1-PP-C2	NP (AMHOrn)	Metachelin C / metachelin A / metachelin A-CE / metachelin B / dimerumic acid 11-mannoside / dimerumic acid
KAF7872880-A	*EAF02_008951*	*Botrytis sinoallii*	PP-C1-A-PP-C2	Phe (Phe)	NP
KAF7892628-A	*EAF02_000166*	*Botrytis sinoallii*	A-PP-C1-PP-C2	NP (AMHOrn)	Metachelin C / metachelin A / metachelin A-CE / metachelin B / dimerumic acid 11-mannoside / dimerumic acid

TGO08137-A	*BTUL_0224g00100*	*Botrytis tulipae*	PP-C1-A-PP-C2	Phe (Phe)	NP
TGO15469-A	*BTUL_0040g00770*	*Botrytis tulipae*	A-PP-C1-PP-C2	NP (AMHOrn)	NP
KAF1949492-A	*CC80DRAFT_555373*	*Byssothecium circinans*	PP-C1-A-PP-C2	NP (Tyr)	NP
KAF1949863-A	*CC80DRAFT_428182*	*Byssothecium circinans*	PP-C1-A-C2	Phe (Phe)	NP
KAF1950193-A	*CC80DRAFT_578867*	*Byssothecium circinans*	PP-C1-A-PP-C2	Phe (Phe)	Aspirochlorine
KAF1957109-A	*CC80DRAFT_515958*	*Byssothecium circinans*	A-PP-C1-PP-PP-C2	Tyr (HMMTyr)	Dimethylcoprogen
KAI4287149-A	*L6R35_003595*	*Caloplaca aegaea*	C1-A-PP-C2	Ival (Ala)	NP
KAI4288945-A	*L6R35_001783*	*Caloplaca aegaea*	C1-A-PP-C2	Phe (Phe)	Acetylaranotin
KAI5919185-A	*F4810DRAFT_518884*	*Camillea tinctor*	A-PP-C1-PP-C2	Ala (2SADD acid)	Dimethylcoprogen
KAI5920513-A	*F4810DRAFT_682235*	*Camillea tinctor*	PP-C1-A-PP-C2	NP (Tyr)	NP
KAI5920857-A	*F4810DRAFT_713056*	*Camillea tinctor*	PP-PP-C1-A-PP-C2	NP (Arg)	NP
KAI5922718-A	*F4810DRAFT_249720*	*Camillea tinctor*	PP-C1-A-PP-C2	Phe (Phe)	NP
GIZ40460-A	*CKM354_000379600*	*Cercospora kikuchii*	C1-A-PP-C2	Phe (Phe)	Aspirochlorine
KAI9649640-A	*NHQ30_002221*	*Ciborinia camelliae*	PP-C1-A-PP-C2	Phe (Phe)	NP
KAI9650511-A	*NHQ30_000527*	*Ciborinia camelliae*	A-PP-C1-PP-C2	NP (AMHOrn)	Metachelin C / metachelin A / metachelin A-CE / metachelin B / dimerumic acid 11-mannoside / dimerumic acid
KAK4459840-A	*QBC42DRAFT_289127*	*Cladorrhinum samala*	A-PP-C1-PP-C2	NP (AMHOrn)	NP
KAK4460133-A	*QBC42DRAFT_339890*	*Cladorrhinum samala*	A-PP-C1-PP-C2	NP (AMHOrn)	Metachelin C / metachelin A / metachelin A-CE / metachelin B / dimerumic acid 11-mannoside / dimerumic acid
KAK4463569-A	*QBC42DRAFT_324105*	*Cladorrhinum samala*	E-C1-A-PP-C2	NP (SHA acid)	Chaetolivacine A / chaetolivacine B / chaetolivacine C
KAK4464486-A	*QBC42DRAFT_336948*	*Cladorrhinum samala*	PP-C1-A-PP-C2	Phe (Phe)	Aspirochlorine
KAF1937772-A	*EJ02DRAFT_385005*	*Clathrospora elynae*	E-C1-A-PP-C2	NP (Asp)	Peramine
KAF1938005-A	*EJ02DRAFT_384572*	*Clathrospora elynae*	PP-C1-A-PP-C2	Phe (Phe)	NP

KAF1941350-A	*EJ02DRAFT_434902*	*Clathrospora elynae*	A-PP-C1-PP-PP-C2	NP (HMMTyr)	Metachelin C / metachelin A / metachelin A-CE / metachelin B / dimerumic acid 11-mannoside / dimerumic acid	
KAF0316791-A	*GQ607_015993*	*Colletotrichum asianum*	PP-E-C1-A-PP-C2	NP (NP)	NP	
KAF0318226-A	*GQ607_014594*	*Colletotrichum asianum*	C1-A-PP-C2	Phe (Phe)	NP	
KAF0325582-A	*GQ607_007024*	*Colletotrichum asianum*	C1-A-PP-C2	Ala (6SMP acid)	NP	T1PKS with DH, cMT, ER and KR domains
KAF0328139-A	*GQ607_004619*	*Colletotrichum asianum*	A-PP-C1-PP-C2	NP (AMHOrn)	Metachelin C / metachelin A / metachelin A-CE / metachelin B / dimerumic acid 11-mannoside / dimerumic acid	
KAK2052305-A	*LY76DRAFT_671583*	*Colletotrichum caudatum*	E-C1-A-PP-C2	NP (NP)	NP	
KAK2054704-A	*LY76DRAFT_663931*	*Colletotrichum caudatum*	PP-C1-A-PP-C2	Phe (Phe)	Penigainamide A / penigainamide B / penigainamide C / adametizine A / FA2097 / outovirin A / outovirin C / pretrichodermamide C	T1PKS with DH, cMT, ER and KR domains
KAK2063014-A	*LY76DRAFT_244099*	*Colletotrichum caudatum*	A-PP-C1-PP-C2	NP (AMHOrn)	Metachelin C / metachelin A / metachelin A-CE / metachelin B / dimerumic acid 11-mannoside / dimerumic acid	
KAJ0364075-A	*COL26b_012664*	*Colletotrichum chrysophilum*	C1-A-PP-C2	NP (Ala)	Chrysogine	

KAJ0380503-A	*COL26b_001208*	*Colletotrichum chrysophilum*	A-PP-C1-PP-C2	NP (AMHOrn)	Metachelin C / metachelin A / metachelin A-CE / metachelin B / dimerumic acid 11-mannoside / dimerumic acid
KAK2013431-A	*LZ32DRAFT_557783*	*Colletotrichum eremochloae*	E-C1-A-PP-C2	NP (NP)	NP
KAK2016423-A	*LZ32DRAFT_487341*	*Colletotrichum eremochloae*	PP-C1-A-PP-C2	Phe (Phe)	Aspirochlorine
KAK2016595-A	*LZ32DRAFT_688938*	*Colletotrichum eremochloae*	A-PP-C1-PP-C2	NP (AMHOrn)	Metachelin C / metachelin A / metachelin A-CE / metachelin B / dimerumic acid 11-mannoside / dimerumic acid
KAF4474935-A	*CGGC5_v016496*	*Colletotrichum fructicola*	C1-A-PP-C2	Ala (6SMP acid)	NP
KAF4476715-A	*CGGC5_v014276*	*Colletotrichum fructicola*	C1-A-PP-C2	Phe (Phe)	NP
KAF4482969-A	*CGGC5_v010030*	*Colletotrichum fructicola*	A-PP-C1-PP-C2	NP (AMHOrn)	Metachelin C / metachelin A / metachelin A-CE / metachelin B / dimerumic acid 11-mannoside / dimerumic acid
KAF4483857-A	*CGGC5_v007960*	*Colletotrichum fructicola*	PP-C1-A-PP-C2	NP (Ser)	Gliovirin
KAF3797717-A	*GCG54_00014615*	*Colletotrichum gloeosporioides*	C1-A-PP-C2	Phe (Phe)	NP
KAF3800591-A	*GCG54_00003489*	*Colletotrichum gloeosporioides*	A-PP-C1-PP-C2	NP (AMHOrn)	Metachelin C / metachelin A / metachelin A-CE / metachelin B / dimerumic acid 11-mannoside / dimerumic acid
KAF3811826-A	*GCG54_00014582*	*Colletotrichum gloeosporioides*	PP-E-C1-A-PP-C2	NP (NP)	NP

KAF9878551-A	*CkaCkLH20_04043*	*Colletotrichum karsti*	A-PP-C1-PP-C2	NP (AMHOrn)	Metachelin C / metachelin A / metachelin A-CE / metachelin B / dimerumic acid 11-mannoside / dimerumic acid	
KAF6816598-A	*CMUS01_12244*	*Colletotrichum musicola*	PP-C1-A-PP-C2	Phe (Phe)	Penigainamide A / penigainamide B / penigainamide C / adametizine A / FA2097 / outovirin A / outovirin C / pretrichodermamide C	
KAF6823353-A	*CMUS01_10736*	*Colletotrichum musicola*	PP-C1-A-PP-C2	Pro (Ala)	NP	
KAF6839137-A	*CMUS01_04384*	*Colletotrichum musicola*	A-PP-C1-PP-C2	NP (AMHOrn)	Metachelin C / metachelin A / metachelin A-CE / metachelin B / dimerumic acid 11-mannoside / dimerumic acid	
KAJ0315918-A	*Brms1b_005755*	*Colletotrichum noveboracense*	PP-E-C1-A-C2	NP (NP)	NP	
KAJ0318744-A	*Brms1b_004233*	*Colletotrichum noveboracense*	PP-C1-A-PP-C2	Ser (Ser)	Gliovirin	
KAJ0322629-A	*Brms1b_002008*	*Colletotrichum noveboracense*	A-PP-C1-PP-C2	NP (AMHOrn)	Metachelin C / metachelin A / metachelin A-CE / metachelin B / dimerumic acid 11-mannoside / dimerumic acid	
TDZ15087-A	*Cob_v011948*	*Colletotrichum orbiculare*	C1-A-PP-C2	Phe (Phe)	NP	T1PKS with DH, cMT, ER and KR domains
TDZ24611-A	*Cob_v002531*	*Colletotrichum orbiculare*	E-C1-A-PP-C2	Pro (NP)	NP	

TDZ25090-A	*Cob_v001899*	*Colletotrichum orbiculare*	A-PP-C1-PP-C2	NP (AMHOrn)	Metachelin C / metachelin A / metachelin A-CE / metachelin B / dimerumic acid 11-mannoside / dimerumic acid
KAF5485085-A	*CGCS363_v013982*	*Colletotrichum siamense*	PP-E-C1-A-PP-C2	NP (NP)	NP
KAF5496972-A	*CGCS363_v009026*	*Colletotrichum siamense*	C1-A-PP-C2	Ala (6SMP acid)	NP
KAF5511477-A	*CGCS363_v002680*	*Colletotrichum siamense*	A-PP-C1-PP-C2	NP (AMHOrn)	Metachelin C / metachelin A / metachelin A-CE / metachelin B / dimerumic acid 11-mannoside / dimerumic acid
TDZ27773-A	*C8035_v008664*	*Colletotrichum spinosum*	C1-A-PP-C2	Phe (Phe)	Aspirochlorine
TDZ34689-A	*C8035_v002636*	*Colletotrichum spinosum*	A-PP-C1-PP-C2	NP (AMHOrn)	Metachelin C / metachelin A / metachelin A-CE / metachelin B / dimerumic acid 11-mannoside / dimerumic acid
TDZ40487-A	*C8035_v004147*	*Colletotrichum spinosum*	C1-A-PP-C2	Pro (NP)	NP
KDN63632-A	*CSUB01_02311*	*Colletotrichum sublineola*	A-PP-C1-PP-C2	NP (AMHOrn)	Metachelin C / metachelin A / metachelin A-CE / metachelin B / dimerumic acid 11-mannoside / dimerumic acid
KDN68423-A	*CSUB01_07544*	*Colletotrichum*	E-C1-A-PP-C2	NP (NP)	NP
KDN70286-A	*CSUB01_11975*	*Colletotrichum*	PP-C1-A-PP-C2	Phe (Phe)	NP

TDZ40339-A	*CTRI78_v010317*	*Colletotrichum trifolii*	A-PP-C1-PP-C2	NP (AMHOrn)	Metachelin C / metachelin A / metachelin A-CE / metachelin B / dimerumic acid 11-mannoside / dimerumic acid
TDZ67766-A	*CTRI78_v002657*	*Colletotrichum trifolii*	PP-C1-A-PP-C2	Phe (Phe)	NP
KAK2023925-A	*LX32DRAFT_706773*	*Colletotrichum zoysiae*	PP-C1-A-PP-C2	Phe (Phe)	Aspirochlorine
KAK2027481-A	*LX32DRAFT_683828*	*Colletotrichum zoysiae*	PP-C1-A-PP-C2	NP (Trp)	Fusarubin / 1233A / 1233B / NG-391 /
KAK2033414-A	*LX32DRAFT_610316*	*Colletotrichum zoysiae*	A-PP-C1-PP-C2	NP (AMHOrn)	Metachelin C / metachelin A / metachelin A-CE / metachelin B / dimerumic acid 11-mannoside / dimerumic acid
PSR78376-A	*BD289DRAFT_486084*	*Coniella lustricola*	A-PP-C1-PP-C2	NP (AMHOrn)	Metachelin C / metachelin A / metachelin A-CE / metachelin B / dimerumic acid 11-mannoside / dimerumic acid
PSR83623-A	*BD289DRAFT_369743*	*Coniella lustricola*	PP-C1-A-PP-C2	Phe (Phe)	NP
PSR99255-A	*BD289DRAFT_48521*	*Coniella lustricola*	C1-A-PP-C2	NP (NP)	NP
PSN66998-A	*BS50DRAFT_552846*	*Corynespora cassiicola*	C1-A-PP-C2	NP (Phe)	Aspirochlorine
PSN73352-A	*BS50DRAFT_482162*	*Corynespora cassiicola*	A-PP-C1-PP-PP-C2	NP (HMMTyr)	Metachelin C / metachelin A / metachelin A-CE / metachelin B / dimerumic acid 11-mannoside / dimerumic acid
KAF3765438-A	*M406DRAFT_37645*	*Cryphonectria parasitica*	A-PP-C1-PP-C2	NP (AMHOrn)	Metachelin C / metachelin A / metachelin A-CE / metachelin B / dimerumic acid 11-mannoside / dimerumic acid
KAF3765631-A	*M406DRAFT_37891*	*Cryphonectria parasitica*	C1-A-PP-C2	NP (NP)	NP
KAF3768171-A	*M406DRAFT_253009*	*Cryphonectria parasitica*	PP-C1-A-PP-C2	Phe (Phe)	NP

KAF1840228-A	*K460DRAFT_348435*	*Cucurbitaria berberidis*	PP-C1-A-PP-C2	Phe (Phe)	NP	
KAF1845025-A	*K460DRAFT_336831*	*Cucurbitaria berberidis*	A-PP-C1-PP-PP-C2	NP (HMMTyr)	Metachelin C / metachelin A / metachelin A-CE / metachelin B / dimerumic acid 11-mannoside / dimerumic acid	
KAI1799247-A	*F4811DRAFT_116497*	*Daldinia bambusicola*	E-C1-A-PP-C2	Pro (Piperazic acid)	NP	
KAI1801775-A	*F4811DRAFT_436855*	*Daldinia bambusicola*	A-PP-C1-PP-C2	Ala (2SADD acid)	Dimethylcoprogen	
KAI1803204-A	*F4811DRAFT_572278*	*Daldinia bambusicola*	PP-C1-A-PP-C2	Phe (Phe)	NP	
KAI1463795-A	*F4812DRAFT_468416*	*Daldinia caldariorum*	A-PP-C1-PP-C2	Ala (2SADD acid)	Dimethylcoprogen	
KAI1468206-A	*F4812DRAFT_358665*	*Daldinia caldariorum*	PP-C1-A-PP-C2	Phe (Phe)	NP	
KAI1471778-A	*F4812DRAFT_192016*	*Daldinia caldariorum*	PP-E-C1-A-PP-C2	NP (NP)	NP	T1PKS with DH and KR domains
KAF3059496-A	*GL218_04719*	*Daldinia childiae*	PP-C1-A-PP-C2	Phe (Phe)	NP	
KAF3065948-A	*GL218_09261*	*Daldinia childiae*	A-PP-C1-PP-C2	Ala (2SADD acid)	Dimethylcoprogen	
KAI1653754-A	*F4813DRAFT_244513*	*Daldinia decipiens*	A-PP-C1-PP-C2	Ala (2SADD acid)	Dimethylcoprogen	
KAI1654151-A	*F4813DRAFT_371661*	*Daldinia decipiens*	C1-A-PP-C2	Ala (NP)	NP	T1PKS with DH and KR domains
KAI1660484-A	*F4813DRAFT_295572*	*Daldinia decipiens*	PP-C1-A-PP-C2	Phe (Phe)	NP	
KAK6952758-A	*Daesc_005052*	*Daldinia eschscholtzii*	PP-C1-A-C2	Phe (Phe)	NP	
KAK6953758-A	*Daesc_003720*	*Daldinia eschscholtzii*	A-PP-C1-PP-C2	Ala (2SADD acid)	Dimethylcoprogen	
KAK6955200-A	*Daesc_002831*	*Daldinia eschscholtzii*	PP-C1-A-PP-C2	NP (Piperazic acid)	NP	T1PKS with DH and KR domains
KAI0121841-A	*F4814DRAFT_192075*	*Daldinia grandis*	PP-C1-A-C2	Phe (Phe)	NP	
KAI0131498-A	*F4814DRAFT_445636*	*Daldinia grandis*	C1-A-PP-C2	NP (NP)	NP	T1PKS with DH and KR domains
KAI0134830-A	*F4814DRAFT_399093*	*Daldinia grandis*	A-PP-C1-PP-C2	Ala (2SADD acid)	Dimethylcoprogen	

KAI1643091-A	*F4817DRAFT_269891*	*Daldinia loculata*	A-PP-C1-PP-C2	Ala (2SADD acid)	Dimethylcoprogen	
KAI1643793-A	*F4817DRAFT_244150*	*Daldinia loculata*	PP-C1-A-PP-C2	Phe (Phe)	NP	
KAI0846350-A	*F5Y00DRAFT_272165*	*Daldinia vernicosa*	PP-C1-A-PP-C2	Phe (Phe)	NP	
KAI0846538-A	*F5Y00DRAFT_271997*	*Daldinia vernicosa*	PP-C1-A-PP-C2	NP (2SHI acid)	NP	T1PKS with DH and KR domains
KAI0846711-A	*F5Y00DRAFT_264325*	*Daldinia vernicosa*	A-PP-C1-PP-C2	Ala (2SADD acid)	Dimethylcoprogen	
KAF2198590-A	*GQ43DRAFT_150754*	*Delitschia confertaspora*	PP-C1-A-PP-C2	NP (Trp)	NP	
KAF2204309-A	*GQ43DRAFT_494648*	*Delitschia confertaspora*	PP-C1-A-PP-C2	Phe (Phe)	NP	
KAJ0120638-A	*J7T55_015368*	*Diaporthe amygdali*	A-PP-C1-PP-C2	NP (AMHOrn)	NP	
KAJ0121088-A	*J7T55_008248*	*Diaporthe amygdali*	C1-A-PP-C2	Phe (Phe)	Aspirochlorine	
KAJ0124390-A	*J7T55_005728*	*Diaporthe amygdali*	C1-A-C2	NP (NP)	NP	
KAL1868956-A	*Daus18300_005792*	*Diaporthe australafricana*	PP-C1-A-PP-C2	NP (Ala)	Astechrome	
KAL1856869-A	*Daus18300_010632*	*Diaporthe australafricana*	C1-A-PP-C2	Phe (Phe)	Penigainamide A / penigainamide B / penigainamide C / adametizine A / FA2097 / outovirin A / outovirin C / pretrichodermamide C	
KAL1856313-A	*Daus18300_010798*	*Diaporthe*	A-PP-C1-PP-C2	NP (AMHOrn)	NP	
KAL1848678-A	*Daus18300_013548*	*Diaporthe*	C1-A-C2	NP (NP)	NP	
POS75433-A	*DHEL01_v206175*	*Diaporthe helianthi*	PP-C1-A-PP-C2	Phe (Phe)	Penigainamide A / penigainamide B / penigainamide C / adametizine A / FA2097 / outovirin A / outovirin C / pretrichodermamide C	
POS78043-A	*DHEL01_v203558*	*Diaporthe helianthi*	C1-A-C2	NP (NP)	NP	
POS79672-A	*DHEL01_v201930*	*Diaporthe helianthi*	PP-C1-A-PP-C2	Phe (Phe)	Aspirochlorine	
POS81277-A	*DHEL01_v200327*	*Diaporthe helianthi*	A-PP-C1-PP-C2	NP (AMHOrn)	NP	
KAJ4350472-A	*N0V89_009093*	*Didymosphaeria*	PP-C1-A-PP-E-C2-	Ala (Ala)	NP	
KAJ4357164-A	*N0V89_001739*	*Didymosphaeria*	PP-C1-A-PP-C2	Phe (Phe)	Aspirochlorine	

OJD28775-A	*BKCO1_1130002*	*Diplodia corticola*	A-PP-C1-PP-C2	NP (AMHOrn)	Metachelin C / metachelin A / metachelin A-CE / metachelin B / dimerumic acid 11-mannoside / dimerumic acid
KAF2128303-A	*P153DRAFT_317943*	*Dothidotthia symphoricarpi*	C1-A-PP-C2	Phe (Phe)	Aspirochlorine
KAF2130328-A	*P153DRAFT_384608*	*Dothidotthia symphoricarpi*	A-PP-C1-PP-PP-C2	NP (HMMTyr)	Metachelin C / metachelin A / metachelin A-CE / metachelin B / dimerumic acid 11-mannoside / dimerumic acid
OSS43307-A	*B5807_12053*	*Epicoccum nigrum*	PP-C1-A-PP-E-C2	NP (Leu)	NP
OSS54489-A	*B5807_01040*	*Epicoccum nigrum*	A-PP-C1-PP-PP-C2	NP (HMMTyr)	Metachelin C / metachelin A / metachelin A-CE / metachelin B / dimerumic acid 11-mannoside / dimerumic acid
KAJ4595950-A	*HRR84_005071*	*Exophiala dermatitidis*	A-PP-C1-PP-C2	NP (AMHOrn)	Metachelin C / metachelin A / metachelin A-CE / metachelin B / dimerumic acid 11-mannoside / dimerumic acid
RVX69419-A	*B0A52_06482*	*Exophiala mesophila*	A-PP-C1-PP-C2	NP (AMHOrn)	Metachelin C / metachelin A / metachelin A-CE / metachelin B / dimerumic acid 11-mannoside / dimerumic acid
RVX72051-A	*B0A52_04649*	*Exophiala mesophila*	C1-A-PP-C2	Phe (Phe)	Acetylaranotin
KAK6363998-A	*LTS17_012627*	*Exophiala oligosperma*	PP-C1-A-PP-C2	Phe (Phe)	Acetylaranotin

KAK6364741-A	*LTS17_011846*	*Exophiala oligosperma*	A-PP-C1-PP-C2	NP (AMHOrn)	Metachelin C / metachelin A / metachelin A-CE / metachelin B / dimerumic acid 11-mannoside / dimerumic acid
KAK6365512-A	*LTS17_011231*	*Exophiala oligosperma*	A-PP-C1-PP-C2	NP (AMHOrn)	Metachelin C / metachelin A / metachelin A-CE / metachelin B / dimerumic acid 11-mannoside / dimerumic acid
KAF5231323-A	*FANTH_13452*	*Fusarium anthophilum*	C1-nMT-PP-C2-A-PP-TD	NP (NCPAla)	NP
KAF5238390-A	*FANTH_10337*	*Fusarium anthophilum*	A-PP-C1-PP-C2	NP (Trp)	NP
KAF5243264-A	*FANTH_8273*	*Fusarium anthophilum*	PP-C1-A-PP-C2	Phe (Phe)	Aspirochlorine
KAF5253754-A	*FANTH_1446*	*Fusarium anthophilum*	C1-A-PP-C2	NP (NP)	NP
KAF4335433-A	*FBEOM_10721*	*Fusarium beomiforme*	PP-C1-A-PP-C2	Phe (Phe)	Acetylaranotin
KAF4339322-A	*FBEOM_6736*	*Fusarium beomiforme*	C1-A-PP-C2	NP (NP)	NP
KAF4345070-A	*FBEOM_945*	*Fusarium beomiforme*	A-PP-C1-PP-C2	NP (Trp)	NP
KAF5977093-A	*FBULB1_6693*	*Fusarium bulbicola*	C1-A-PP-C2	NP (NP)	NP
KAF5979554-A	*FBULB1_5702*	*Fusarium bulbicola*	A-PP-C1-PP-C2	NP (Trp)	NP
KAF5964526-A	*FCOIX_13379*	*Fusarium coicis*	A-C1-PP-C2	NP (AMHOrn*)	NP
KAF5966553-A	*FCOIX_12449*	*Fusarium coicis*	PP-C1-A-PP-C2	Phe (Phe)	Penigainamide A / penigainamide B / penigainamide C / adametizine A / FA2097 / outovirin A / outovirin C / pretrichodermamide C
KAF5981065-A	*FCOIX_4403*	*Fusarium coicis*	A-PP-C1-PP-C2	NP (Trp)	NP
CCT68163-A	*FFUJ_06929*	*Fusarium fujikuroi*	C1-A-PP-C2	NP (NP)	NP
CCT69460-A	*FFUJ_05347*	*Fusarium fujikuroi*	PP-C1-A-PP-C2	Phe (Phe)	Acetylaranotin
CCT74675-A	*FFUJ_10736*	*Fusarium fujikuroi*	A-PP-C1-PP-C2	NP (Trp)	NP

KAF4948216-A	*FGADI_9808*	*Fusarium gaditjirri*	PP-C1-A-PP-C2-AmT	Phe (Phe)	Penigainamide A / penigainamide B / penigainamide C / adametizine A / FA2097 / outovirin A / outovirin C / pretrichodermamide C
KAF4961589-A	*FGADI_213*	*Fusarium gaditjirri*	A-PP-C1-PP-C2	NP (Trp)	NP
KAF5710403-A	*FGLOB1_5493*	*Fusarium globosum*	PP-C1-A-PP-C2	Phe (Phe)	Penigainamide A / penigainamide B / penigainamide C / adametizine A / FA2097 / outovirin A / outovirin C / pretrichodermamide C
KAF5716714-A	*FGLOB1_2434*	*Fusarium globosum*	C1-A-PP-C2	NP (NP)	NP
KAF5721595-A	*FGLOB1_139*	*Fusarium globosum*	A-PP-C1-PP-C2	NP (Trp)	NP
CVK92064-A	*FMAN_07066*	*Fusarium mangiferae*	C1-A-PP-C2	NP (NP)	NP
CVK95614-A	*FMAN_13615*	*Fusarium mangiferae*	A-PP-C1-PP-C2	NP (Trp)	NP
CVK98965-A	*FMAN_08343*	*Fusarium mangiferae*	PP-C1-A-PP-C2	Phe (Phe)	Aspirochlorine
KAF5535871-A	*FMEXI_10603*	*Fusarium mexicanum*	C1-A-PP-C2	NP (NP)	NP
KAF5543114-A	*FMEXI_7189*	*Fusarium mexicanum*	A-C1-E-C2	Trp (Trp)	Fusarochromanone / desacetylfusarochromene / fusarochromene / 4-hydroxykynurenine
KAF5557429-A	*FMEXI_752*	*Fusarium mexicanum*	A-PP-C1-PP-C2	NP (Trp)	NP
KAG8672856-A	*FPOAC1_006151*	*Fusarium musae*	A-PP-C1-PP-C2	NP (Trp)	NP
KAG8677647-A	*FPOAC1_003672*	*Fusarium musae*	C1-A-PP-C2	NP (NP)	NP
KAG9496067-A	*J7337_012642*	*Fusarium musae*	A-PP-C1-PP-C2	NP (Trp)	NP
KAG9503295-A	*J7337_006138*	*Fusarium musae*	C1-A-PP-C2	NP (NP)	NP
KAF5562361-A	*FNAPI_3257*	*Fusarium napiforme*	PP-C1-A-PP-C2	Phe (Phe)	Penigainamide A / penigainamide B / penigainamide C / adametizine A / FA2097 / outovirin A / outovirin C / pretrichodermamide C

KAF5565035-A	*FNAPI_1854*	*Fusarium napiforme*	C1-A-PP-C2	NP (NP)	NP
KAF5565206-A	*FNAPI_1741*	*Fusarium napiforme*	A-PP-C1-PP-C2	NP (Trp)	NP
CZR43611-A	*FPRO_07472*	*Fusarium proliferatum*	C1-A-PP-C2	NP (NP)	NP
CZR44510-A	*FPRO_14263*	*Fusarium proliferatum*	A-PP-C1-PP-C2	NP (Trp)	NP
CZR49444-A	*FPRO_08815*	*Fusarium proliferatum*	PP-C1-A-PP-C2	Phe (Phe)	Acetylaranotin
KAF5572592-A	*FPANT_12972*	*Fusarium pseudoanthophilum*	PP-C1-A-PP-C2	Phe (Phe)	Penigainamide A / penigainamide B / penigainamide C / adametizine A / FA2097 / outovirin A / outovirin C / pretrichodermamide C
KAF5592482-A	*FPANT_5405*	*Fusarium pseudoanthophilum*	C1-A-PP-C2	NP (NP)	NP
KAF5608689-A	*FPANT_392*	*Fusarium pseudoanthophilum*	A-PP-C1-PP-C2	NP (Trp)	NP
KAF5581613-A	*FPCIR_10055*	*Fusarium pseudocircinatum*	PP-C1-A-PP-C2	Phe (Phe)	NP
KAF5586055-A	*FPCIR_8075*	*Fusarium pseudocircinatum*	C1-A-PP-C2	NP (NP)	NP
KAJ3459448-A	*MRS44_015521*	*Fusarium solani*	A-PP-C1-PP-C2	NP (Trp)	NP
KAJ3461906-A	*MRS44_010459*	*Fusarium solani*	A-PP-C1-PP-C2	NP (AMHOrn)	Metachelin C / metachelin A / metachelin A-CE / metachelin B / dimerumic acid 11-mannoside / dimerumic acid
KAJ3471879-A	*MRS44_001978*	*Fusarium solani*	C1-A-PP-C2	NP (NP)	NP
KAF5623762-A	*FTJAE_10523*	*Fusarium tjaetaba*	PP-C1-A-PP-C2-AmT	Phe (Phe)	Penigainamide A / penigainamide B / penigainamide C / adametizine A / FA2097 / outovirin A / outovirin C / pretrichodermamide C
KAF5627738-A	*FTJAE_9136*	*Fusarium tjaetaba*	A-PP-C1-PP-C2	NP (Trp)	NP
KAF5637380-A	*FTJAE_5795*	*Fusarium tjaetaba*	C1-A-C2	NP (NP)	NP

RBQ98941-A	*FVER53263_03243*	*Fusarium verticillioides*	C1-A-PP-C2	NP (NP)	NP	
RBQ99980-A	*FVER53263_08697*	*Fusarium verticillioides*	A-PP-C1-PP-C2	NP (Trp)	NP	
OCL06618-A	*AOQ84DRAFT_296655*	*Glonium stellatum*	C1-A-PP-C2	Phe (Phe)	Aspirochlorine	
OCL04400-A	*AOQ84DRAFT_416366*	*Glonium stellatum*	A-PP-C1-PP-C2	NP (AMHOrn)	Metachelin C / metachelin A / metachelin A-CE / metachelin B / dimerumic acid 11-mannoside / dimerumic acid	
KAH6666328-A	*B0J14DRAFT_643166*	*Halenospora varia*	PP-C1-A-PP-C2	Phe (Phe)	Aspirochlorine	
KAI1775853-A	*F4818DRAFT_389984*	*Hypoxylon cercidicola*	A-PP-C1-PP-C2	Ala (Pro)	Dimethylcoprogen	
KAI1778794-A	*F4818DRAFT_229631*	*Hypoxylon cercidicola*	PP-C1-A-PP-C2	Phe (Phe)	Aspirochlorine	
KAI1405013-A	*F4819DRAFT_502670*	*Hypoxylon fuscum*	PP-C1-A-C2	NP (NP)	NP	
KAI4864703-A	*F4820DRAFT_326513*	*Hypoxylon rubiginosum*	A-PP-C1-PP-C2	Ala (Pro)	Dimethylcoprogen	
MCJ1352279-A	*MMC33_002263*	*Icmadophila ericetorum*	PP-C1-A-PP-C2	Phe (Phe)	Aspirochlorine	
KAK0612095-A	*B0T14DRAFT_489412*	*Immersiella caudata*	PP-PP-E-C1-A-PP-	Pro (NP)	NP	
KAK0613181-A	*B0T14DRAFT_437412*	*Immersiella caudata*	A-PP-C1-PP-C2	NP (AMHOrn)	NP	
KAK0617477-A	*B0T14DRAFT_590199*	*Immersiella caudata*	PP-C1-A-PP-C2	Phe (Phe)	Acetylaranotin	T1PKS with DH, cMT, ER and KR domains
KAI1103822-A	*F4804DRAFT_308935*	*Jackrogersella minutella*	A-PP-C1-PP-C2	Ala (2SADD acid)	Dimethylcoprogen	
KAI1104793-A	*F4804DRAFT_331908*	*Jackrogersella minutella*	C1-A-PP-C2	NP (SBHEA)	NP	
KAI1105352-A	*F4804DRAFT_304606*	*Jackrogersella minutella*	C1-A-PP-C2	Phe (Phe)	Acetylaranotin	
KAF2446656-A	*P171DRAFT_453957*	*Karstenula rhodostoma*	C1-A-PP-C2	Phe (Phe)	Aspirochlorine	
KAF2449195-A	*P171DRAFT_516953*	*Karstenula rhodostoma*	A-PP-C1-PP-PP-C2	Tyr (HMMTyr)	Metachelin C / metachelin A / metachelin A-CE / metachelin B / dimerumic acid 11-mannoside / dimerumic acid	
KAJ9628429-A	*H2204_009266*	*Knufia peltigerae*	A-PP-C1-PP-C2	NP (AMHOrn)	NP	

KAJ9634430-A	*H2204_006255*	*Knufia peltigerae*	A-PP-C1-PP-C2	NP (AMHOrn)	Metachelin C / metachelin A / metachelin A-CE / metachelin B / dimerumic acid 11-mannoside / dimerumic acid
TVY93795-A	*LAWI1_G001474*	*Lachnellula willkommii*	C1-A-PP-C2	Phe (Phe)	Penigainamide A / penigainamide B / penigainamide C / adametizine A / FA2097 / outovirin A / outovirin C / pretrichodermamide C
KAJ2975050-A	*NQ176_g5733*	*Lecanicillium fungicola*	PP-C1-A-PP-C2	Phe (Phe)	NP
KAJ2983224-A	*NQ176_g857*	*Lecanicillium fungicola*	A-PP-C1-PP-C2	NP (AMHOrn)	Metachelin C / metachelin A / metachelin A-CE / metachelin B / dimerumic acid 11-mannoside / dimerumic acid
KAJ2983267-A	*NQ176_g816*	*Lecanicillium fungicola*	PP-C1-A-PP-C2	NP (Ala)	NP
KAF2677297-A	*K458DRAFT_378803*	*Lentithecium fluviatile*	C1-A-PP-C2	Phe (Phe)	Aspirochlorine
KAF2689882-A	*K458DRAFT_289563*	*Lentithecium fluviatile*	A-PP-C1-PP-PP-C2	NP (HMMTyr)	Metachelin C / metachelin A / metachelin A-CE / metachelin B / dimerumic acid 11-mannoside / dimerumic acid
KAK3167851-A	*OEA41_004297*	*Lepraria neglecta*	C1-A-PP-C2	Phe (Phe)	NP
KAF1362625-A	*EJ07DRAFT_105106*	*Lizonia empirigonia*	PP-C1-A-PP-C2	NP (Phe)	Penigainamide A / penigainamide B / penigainamide C / adametizine A / FA2097 / outovirin A / outovirin C / pretrichodermamide C

KAF1363150-A	*EJ07DRAFT_104604*	*Lizonia empirigonia*	A-PP-C1-PP-PP-C2	NP (HMMTyr)	Metachelin C / metachelin A / metachelin A-CE / metachelin B / dimerumic acid 11-mannoside / dimerumic acid
KAF1365607-A	*EJ07DRAFT_150123*	*Lizonia empirigonia*	C1-A-PP-C2	Ala (Ala)	NP
MCJ1269302-A	*MMC22_009193*	*Lobaria immixta*	PP-C1-A-PP-C2	Phe (Phe)	Gliovirin
KAF2259674-A	*CC78DRAFT_555928*	*Lojkania enalia*	PP-C1-A-PP-C2	Phe (Phe)	Aspirochlorine
KAF2259745-A	*CC78DRAFT_53242*	*Lojkania enalia*	A-PP-C1-PP-PP-C2	NP (HMMTyr)	Metachelin C / metachelin A / metachelin A-CE / metachelin B / dimerumic acid 11-mannoside / dimerumic acid
KAF2106355-A	*BDV96DRAFT_625896*	*Lophiotrema nucula*	PP-C1-A-PP-C2	Phe (Phe)	Penigainamide A / penigainamide B / penigainamide C / adametizine A / FA2097 / outovirin A / outovirin C / pretrichodermamide C
KAF2110732-A	*BDV96DRAFT_650835*	*Lophiotrema nucula*	A-PP-C1-PP-PP-C2	NP (HMMTyr)	Metachelin C / metachelin A / metachelin A-CE / metachelin B / dimerumic acid 11-mannoside / dimerumic acid
KAF2122844-A	*BDV96DRAFT_608694*	*Lophiotrema nucula*	PP-C1-A-PP-C2	Phe (Phe)	Aspirochlorine
KAF2490812-A	*BU16DRAFT_595397*	*Lophium mytilinum*	PP-C1-A-PP-C2	Phe (Phe)	NP
KAF2498220-A	*BU16DRAFT_570781*	*Lophium mytilinum*	A-PP-C1-PP-C2	NP (AMHOrn)	Metachelin C / metachelin A / metachelin A-CE / metachelin B / dimerumic acid 11-mannoside / dimerumic acid

GAB1312644-A	*MFIFM68171_02854*	*Madurella fahalii*	A-PP-C1-PP-C2	NP (AMHOrn)	Metachelin C / metachelin A / metachelin A-CE / metachelin B / dimerumic acid 11-mannoside / dimerumic acid	
GAB1316519-A	*MFIFM68171_06729*	*Madurella fahalii*	A-PP-C1-C2	NP (Leu)	NP	T1PKS with DH, cMT, ER and KR domains
GAB1317434-A	*MFIFM68171_07644*	*Madurella fahalii*	C1-A-PP-C2	Phe (Phe)	NP	
KXX75868-A	*MMYC01_209074*	*Madurella mycetomatis*	A-PP-C1-PP-C2	NP (AMHOrn)	Metachelin C / metachelin A / metachelin A-CE / metachelin B / dimerumic acid 11-mannoside / dimerumic acid	
KXX77992-A	*MMYC01_205164*	*Madurella mycetomatis*	C1-A-PP-C2	Phe (Phe)	Aspirochlorine	
KXX83232-A	*MMYC01_200280*	*Madurella mycetomatis*	C1-A-PP-C2	Phe (Phe)	Aspirochlorine	
KAF2793291-A	*K505DRAFT_362133*	*Melanomma pulvis-pyrius*	A-PP-C1-PP-PP-C2	NP (Arg)	Metachelin C / metachelin A / metachelin A-CE / metachelin B / dimerumic acid 11-mannoside / dimerumic acid	
KAA8571073-A	*EYC84_000428*	*Monilinia fructicola*	PP-C1-A-PP-C2	Phe (Phe)	NP	
KAB8298143-A	*EYC80_001899*	*Monilinia laxa*	PP-C1-A-PP-C2	Phe (Phe)	NP	
KAB8302857-A	*EYC80_006191*	*Monilinia laxa*	A-PP-C1-PP-C2	NP (AMHOrn)	Metachelin C / metachelin A / metachelin A-CE / metachelin B / dimerumic acid 11-mannoside / dimerumic acid	
QSZ30080-A	*DSL72_004600*	*Monilinia vaccinii-corymbosi*	A-PP-C1-PP-C2	NP (AMHOrn)	Metachelin C / metachelin A / metachelin A-CE / metachelin B / dimerumic acid 11-mannoside / dimerumic acid	

QSZ34268-A	*DSL72_005858*	*Monilinia vaccinii-corymbosi*	PP-C1-A-PP-C2	Phe (Phe)	NP
RYO92810-A	*DL764_008084*	*Monosporascus ibericus*	A-PP-C1-PP-C2	Ala (2SADD acid)	Dimethylcoprogen
RYP04563-A	*DL764_004371*	*Monosporascus ibericus*	PP-C1-A-PP-C2	Phe (Phe)	Aspirochlorine
KAF2801822-A	*BDZ99DRAFT_483370*	*Mytilinidion resinicola*	A-PP-C1-PP-C2	NP (AMHOrn)	Metachelin C / metachelin A / metachelin A-CE / metachelin B / dimerumic acid 11-mannoside / dimerumic acid
KAF2809436-A	*BDZ99DRAFT_531747*	*Mytilinidion resinicola*	PP-C1-A-PP-C2	Phe (Phe)	NP
EFQ97796-A	*MGYG_00836*	*Nannizzia gypsea*	A-PP-C1-PP-C2-PP	NP (Ser)	NP
EFQ99850-A	*MGYG_02862*	*Nannizzia gypsea*	A-PP-C1-PP-C2	Trp (Trp)	NP
EFR00303-A	*MGYG_03304*	*Nannizzia gypsea*	PP-C1-A-PP-C2	Phe (Phe)	Aspirochlorine
KAI1120239-A	*F5Y10DRAFT_290157*	*Nemania abortiva*	PP-C1-A-PP-C2	Phe (Phe)	Aspirochlorine
KAI1120260-A	*F5Y10DRAFT_290050*	*Nemania abortiva*	PP-C1-A-PP-C2	Phe (Phe)	NP
KAI1121805-A	*F5Y10DRAFT_282207*	*Nemania abortiva*	PP-C1-A-PP-C2	NP (Piperazic	NP
KAI1130283-A	*F5Y10DRAFT_289738*	*Nemania abortiva*	A-PP-C1-PP-C2	NP (Pro)	NP
KAI1144963-A	*F4825DRAFT_445056*	*Nemania diffusa*	C1-A-PP-C2-PP-E	NP (Benzoxazolinate)	NP
KAI1151500-A	*F4825DRAFT_462577*	*Nemania diffusa*	C1-A-PP-C2	NP (Piperazic acid)	NP
KAI1151527-A	*F4825DRAFT_353948*	*Nemania diffusa*	A-PP-C1-PP-C2	NP (Trp)	Dimethylcoprogen
KAI1200699-A	*F5X97DRAFT_77716*	*Nemania serpens*	A-PP-C1-PP-C2	NP (Trp)	Dimethylcoprogen
KAI1201602-A	*F5X97DRAFT_290058*	*Nemania serpens*	PP-C1-A-PP-C2	NP (Arg)	NP
KAJ4371135-A	*N0V83_004351*	*Neocucurbitaria cava*	A-PP-C1-PP-PP-C2	NP (HMMTyr)	Metachelin C / metachelin A / metachelin A-CE / metachelin B / dimerumic acid 11-mannoside / dimerumic acid
KAJ4375875-A	*N0V83_001153*	*Neocucurbitaria cava*	C1-A-PP-C2	Phe (Phe)	NP

KIM94802-A	*OIDMADRAFT_60571*	*Oidiodendron maius*	C1-A-PP-C2	NP (2-aminoisobutyric acid)	UNII-YC2Q1O94PT	T1PKS with DH, cMT, ER and KR domains
KIM95670-A	*OIDMADRAFT_171109*	*Oidiodendron maius*	PP-C1-A-PP-C2	Phe (Phe)	Aspirochlorine	
KIM97226-A	*OIDMADRAFT_203951*	*Oidiodendron maius*	C1-A-PP-C2	Leu (leu)	NP	
KAI1938759-A	*LOZ57_006305*	*Ophidiomyces ophidiicola*	A-PP-C1-PP-C2	NP (Pro)	Metachelin C / metachelin A / metachelin A-CE / metachelin B / dimerumic acid 11-mannoside / dimerumic acid	
KAI1942313-A	*LOZ57_005337*	*Ophidiomyces*	A-PP-C1-PP-C2	Trp (Trp)	NP	
KAI1942954-A	*LOZ57_005251*	*Ophidiomyces ophidiicola*	PP-C1-A-PP-C2	Phe (6SMP acid)	Penigainamide A / penigainamide B / penigainamide C / adametizine A / FA2097 / outovirin A / outovirin C / pretrichodermamide C	
KAK4031934-A	*C8A01DRAFT_20928*	*Parachaetomium inaequale*	PP-C1-A-PP-C2	Phe (Phe)	NP	
KAK4032257-A	*C8A01DRAFT_41308*	*Parachaetomium inaequale*	A-PP-C1-PP-C2	NP (AMHOrn)	NP	
KAK4035449-A	*C8A01DRAFT_17873*	*Parachaetomium inaequale*	E-C1-A-PP-C2	Pro (NP)	NP	
KAL1591655-A	*SLS60_011653*	*Paraconiothyrium brasiliense*	A-PP-C1-PP-PP-C2	Tyr (HMMTyr)	Metachelin C / metachelin A / metachelin A-CE / metachelin B / dimerumic acid 11-mannoside / dimerumic acid	
KAL1599497-A	*SLS60_007300*	*Paraconiothyrium brasiliense*	PP-C1-A-PP-C2	Pro (Ala)	NP	
KAL1604776-A	*SLS60_004316*	*Paraconiothyrium brasiliense*	PP-C1-A-PP-C2	Phe (Phe)	Aspirochlorine	
OAG08642-A	*CC84DRAFT_1239987*	*Paraphaeosphaeria sporulosa*	PP-C1-A-PP-C2	Phe (Phe)	NP	

OAG12374-A	*CC84DRAFT_1080080*	*Paraphaeosphaeria sporulosa*	A-PP-C1-PP-PP-C2	Tyr (HMMTyr)	Metachelin C / metachelin A / metachelin A-CE / metachelin B / dimerumic acid 11-mannoside / dimerumic acid	
KAJ5288884-A	*N7478_001914*	*Penicillium angulare*	A-PP-C1-PP-C2	NP (AMHOrn)	Metachelin C / metachelin A / metachelin A-CE / metachelin B / dimerumic acid 11-mannoside / dimerumic acid	
OGE48516-A	*PENARI_c028G08770*	*Penicillium arizonense*	C1-A-PP-C2	Phe (Phe)	Acetylaranotin	
OGE53631-A	*PENARI_c007G01568*	*Penicillium arizonense*	A-PP-C1-PP-C2	NP (AMHOrn)	NP	
OGE56315-A	*PENARI_c003G00612*	*Penicillium arizonense*	A-PP-C1-PP-C2	NP (AMHOrn)	Metachelin C / metachelin A / metachelin A-CE / metachelin B / dimerumic acid 11-mannoside / dimerumic acid	
CRL18528-A	*PCAMFM013_S002g000398*	*Penicillium camemberti*	A-PP-C1-PP-C2	NP (AMHOrn)	NP	
CRL19235-A	*PCAMFM013_S003g000026*	*Penicillium camemberti*	A-PP-C1-PP-C2	NP (AMHOrn)	Metachelin C / metachelin A / metachelin A-CE / metachelin B / dimerumic acid 11-mannoside / dimerumic acid	T1PKS with DH, ER and KR domains
KAJ6042402-A	*N7446_013468*	*Penicillium canescens*	A-PP-C1-PP-C2	NP (AMHOrn)	Metachelin C / metachelin A / metachelin A-CE / metachelin B / dimerumic acid 11-mannoside / dimerumic acid	
KAJ6048215-A	*N7446_010898*	*Penicillium canescens*	A-PP-C1-PP-C2	Trp (Trp)	NP	

KAJ6077522-A	*N7446_000458*	*Penicillium canescens*	A-PP-C1-PP-C2	NP (AMHOrn)	Metachelin C / metachelin A / metachelin A-CE / metachelin B / dimerumic acid 11-mannoside / dimerumic acid	
KAJ5358649-A	*N7496_011062*	*Penicillium cataractarum*	A-PP-C1-PP-C2	NP (3SMAsp acid)	NP	
KAJ5368012-A	*N7496_007772*	*Penicillium cataractarum*	A-PP-C1-PP-C2	NP (AMHOrn)	NP	
KAJ5381434-A	*N7496_003862*	*Penicillium cataractarum*	PP-C1-A-PP-C2	Phe (Phe)	Penigainamide A / penigainamide B / penigainamide C / adametizine A / FA2097 / outovirin A / outovirin C / pretrichodermamide C	
KAJ5249273-A	*N7468_000724*	*Penicillium chermesinum*	PP-C1-A-PP-C2	Phe (Phe)	Acetylaranotin	
KAJ5373177-A	*N7517_005183*	*Penicillium concentricum*	A-PP-C1-PP-C2	NP (AMHOrn)	NP	
KAJ5375404-A	*N7517_007410*	*Penicillium concentricum*	PP-C1-A-PP-C2	NP (Phe)	NP	
KAJ5382955-A	*N7517_000866*	*Penicillium concentricum*	A-PP-C1-PP-C2	NP (AMHOrn)	Metachelin C / metachelin A / metachelin A-CE / metachelin B / dimerumic acid 11-mannoside / dimerumic acid	T1PKS with DH, ER and KR domains
KGO51028-A	*PEX2_094940*	*Penicillium expansum*	A-PP-C1-PP-C2	NP (AMHOrn)	NP	
KGO52259-A	*PEX2_109110*	*Penicillium expansum*	PP-C1-A-PP-C2	NP (Phe)	Andrastin A	T1PKS with SAT, DH and cMT domains, and T1PKS with DH, ER and KR domains
CAI7566436-A	*PGLAN3C_LOCUS264*	*Penicillium glandicola*	A-PP-C1-PP-C2	NP (AMHOrn)	NP	

CAI7588210-A	*PGLAN3C_LOCUS2480*	*Penicillium glandicola*	A-PP-C1-PP-C2	NP (AMHOrn)	Metachelin C / metachelin A / metachelin A-CE / metachelin B / dimerumic acid 11-mannoside / dimerumic acid	T1PKS with DH, ER and KR domains
CAI7596063-A	*PGLAN3C_LOCUS3267*	*Penicillium glandicola*	C1-A-PP-C2	Asp (Glu)	NP	
CAI7613184-A	*PGLAN3C_LOCUS5018*	*Penicillium glandicola*	PP-C1-A-PP-C2	NP (Leu)	NP	
CAI7629519-A	*PGLAN3C_LOCUS6663*	*Penicillium glandicola*	PP-C1-A-PP-C2	NP (Phe)	NP	
KXG46753-A	*PGRI_034990*	*Penicillium griseofulvum*	A-PP-C1-PP-C2	NP (AMHOrn)	Metachelin C / metachelin A / metachelin A-CE / metachelin B / dimerumic acid 11-mannoside / dimerumic acid	T1PKS with DH, ER and KR domains
KXG54093-A	*PGRI_072370*	*Penicillium griseofulvum*	A-PP-C1-PP-C2	NP (AMHOrn)	NP	
KAJ5589047-A	*N7537_011725*	*Penicillium hordei*	A-PP-C1-PP-C2	NP (AMHOrn)	Metachelin C / metachelin A / metachelin A-CE / metachelin B / dimerumic acid 11-mannoside / dimerumic acid	Two T1PKS with DH, ER and KR domains
KAJ5603921-A	*N7537_006877*	*Penicillium hordei*	C1-A-PP-C2	Phe (Phe)	Acetylaranotin	
KAJ5608003-A	*N7537_004622*	*Penicillium hordei*	A-PP-C1-PP-C2	NP (AMHOrn)	NP	
KGO66584-A	*PITC_079840*	*Penicillium italicum*	PP-C1-A-PP-C2	NP (Phe)	NP	
KGO76863-A	*PITC_004900*	*Penicillium italicum*	A-PP-C1-PP-C2	NP (AMHOrn)	NP	
KAJ5639099-A	*N7484_006961*	*Penicillium longicatenatum*	A-PP-C1-PP-C2	NP (AMHOrn)	Metachelin C / metachelin A / metachelin A-CE / metachelin B / dimerumic acid 11-mannoside / dimerumic acid	
KAJ5649907-A	*N7484_003630*	*Penicillium longicatenatum*	A-PP-C1-PP-C2	NP (R-beta-Phe)	Sordarial	T1PKS with SAT, DH and cMT domains, and T1PKS with DH, ER and KR domains

KAJ5661076-A	*N7484_000448*	*Penicillium longicatenatum*	C1-A-PP-C2	Phe (Phe)	Acetylaranotin	
KOS38153-A	*ACN38_g11035*	*Penicillium nordicum*	C1-A-PP-C2	Phe (Phe)	Acetylaranotin	
KOS38326-A	*ACN38_g10847*	*Penicillium nordicum*	A-PP-C1-PP-C2	NP (AMHOrn)	NP	
KOS42736-A	*ACN38_g6384*	*Penicillium nordicum*	A-PP-C1-PP-C2	NP (AMHOrn)	Metachelin C / metachelin A / metachelin A-CE / metachelin B / dimerumic acid 11-mannoside / dimerumic acid	
KAF3396087-A	*F1880_007220*	*Penicillium rolfsii*	PP-C1-A-PP-C2	Phe (Phe)	Penigainamide A / penigainamide B / penigainamide C / adametizine A / FA2097 / outovirin A / outovirin C / pretrichodermamide C	
KAF3399990-A	*F1880_008096*	*Penicillium rolfsii*	A-PP-C1-PP-C2	NP (AMHOrn)	Oxaleimide	
KAF3402030-A	*F1880_009960*	*Penicillium rolfsii*	A-PP-C1-PP-C2	His (R-beta-Phe)	Sordarial	T1PKS with DH, ER and KR domains
OQE19175-A	*PENSTE_c016G06968*	*Penicillium steckii*	A-PP-C1-PP-C2	NP (AMHOrn)	Metachelin C / metachelin A / metachelin A-CE / metachelin B / dimerumic acid 11-mannoside / dimerumic acid	
OQE22569-A	*PENSTE_c010G02589*	*Penicillium steckii*	PP-C1-A-PP-C2	Phe (Phe)	Penigainamide A / penigainamide B / penigainamide C / adametizine A / FA2097 / outovirin A / outovirin C / pretrichodermamide C	
OQE29477-A	*PENSTE_c002G01760*	*Penicillium steckii*	A-PP-C1-PP-C2	NP (AMHOrn)	NP	
KAJ9481718-A	*VN97_g11748*	*Penicillium thymicola*	PP-C1-A-PP-C2	Ala (Ala)	BII-rafflesfungin	

KAJ9485221-A	*VN97_g8138*	*Penicillium thymicola*	A-PP-C1-PP-C2	NP (AMHOrn)	Metachelin C / metachelin A / metachelin A-CE / metachelin B / dimerumic acid 11-mannoside / dimerumic acid	T1PKS with DH, ER and KR domains
KAJ9488960-A	*VN97_g4325*	*Penicillium thymicola*	C1-A-PP-C2	Phe (Phe)	Acetylaranotin	
KAJ9492309-A	*VN97_g923*	*Penicillium thymicola*	A-PP-C1-PP-C2	NP (AMHOrn)	NP	
KAJ5926620-A	*N7516_008393*	*Penicillium verrucosum*	A-PP-C1-PP-C2	NP (AMHOrn)	Metachelin C / metachelin A / metachelin A-CE / metachelin B / dimerumic acid 11-mannoside / dimerumic acid	Two T1PKS with DH, ER and KR domains
KAJ5931744-A	*N7516_006233*	*Penicillium verrucosum*	A-PP-C1-PP-C2	NP (AMHOrn)	NP	
KAJ5940503-A	*N7516_000671*	*Penicillium verrucosum*	C1-A-PP-C2	Phe (Phe)	Acetylaranotin	
CAI6288118-A	*PDIGIT_LOCUS2378*	*Periconia digitata*	PP-C1-A-PP-C2	Phe (Phe)	NP	
CAI6326507-A	*PDIGIT_LOCUS3837*	*Periconia digitata*	A-PP-C1-PP-PP-C2	Tyr (HMMTyr)	Metachelin C / metachelin A / metachelin A-CE / metachelin B / dimerumic acid 11-mannoside / dimerumic acid	
CAI6334286-A	*PDIGIT_LOCUS7343*	*Periconia digitata*	PP-C1-A-PP-C2	Phe (Phe)	Aspirochlorine	
CAI6342491-A	*PDIGIT_LOCUS15698*	*Periconia digitata*	nMT-PP-C1-A-PP-E-C2	NP (Arg)	NP	
PVI00661-A	*DM02DRAFT_562775*	*Periconia macrospinosa*	PP-C1-A-PP-C2	Phe (Phe)	NP	
PVI02653-A	*DM02DRAFT_701133*	*Periconia macrospinosa*	PP-C1-A-PP-C2	Phe (Phe)	Aspirochlorine	T1PKS with DH, ER and KR domains
PVI02684-A	*DM02DRAFT_522428*	*Periconia macrospinosa*	PP-C1-A-PP-PP-C2	Phe (Phe)	NP	
PVI04083-A	*DM02DRAFT_556789*	*Periconia macrospinosa*	A-PP-C1-PP-PP-C2	Tyr (HMMTyr)	Metachelin C / metachelin A / metachelin A-CE / metachelin B / dimerumic acid 11-mannoside / dimerumic acid	

ETS73670-A	*PFICI_14616*	*Pestalotiopsis fici*	A-PP-C1-PP-C2	NP (Trp)	Dimethylcoprogen
ETS77601-A	*PFICI_09663*	*Pestalotiopsis fici*	PP-C1-A-PP-C2	Phe (Phe)	Aspirochlorine
ETS80856-A	*PFICI_08385*	*Pestalotiopsis fici*	PP-C1-A-C2	NP (Piperazic acid)	NP
ETS84288-A	*PFICI_02313*	*Pestalotiopsis fici*	PP-C1-A-PP-C2	Ala (Ala)	NP
KAL3421707-A	*PVAG01_05863*	*Phlyctema vagabunda*	C1-A-PP-C2	NP (Trp)	NP
KAL3422110-A	*PVAG01_06266*	*Phlyctema vagabunda*	C1-A-PP-C2	Phe (Phe)	Aspirochlorine
KAL3420092-A	*PVAG01_08591*	*Phlyctema vagabunda*	PP-C1-A-PP-C2	Ala (PC acid)	NP
KAL3418386-A	*PVAG01_10102*	*Phlyctema vagabunda*	A-PP-C1-PP-C2	NP (AMHOrn)	Metachelin C / metachelin A / metachelin A-CE / metachelin B / dimerumic acid 11-mannoside / dimerumic acid
KAG6988951-A	*G7Y79_00067g095660*	*Physcia stellaris*	C1-A-PP-C2	Phe (Phe)	NP
KAG7006651-A	*G7Y79_00013g034230*	*Physcia stellaris*	PP-C1-A-PP-C2-PP	NP (Leu)	NP
KAH9864443-A	*J1614_010377*	*Plenodomus biglobosus*	A-PP-C1-PP-PP-C2	NP (HMMTyr)	Metachelin C / metachelin A / metachelin A-CE / metachelin B / dimerumic acid 11-mannoside / dimerumic acid
KAH9861152-A	*IAQ61_010889*	*Plenodomus lingam*	A-PP-C1-PP-PP-C2	NP (HMMTyr)	Metachelin C / metachelin A / metachelin A-CE / metachelin B / dimerumic acid 11-mannoside / dimerumic acid
KAI9880683-A	*M1830_001316*	*Pleopsidium flavum*	C1-A-PP-C2	Phe (Phe)	Aspirochlorine
KAK4184506-A	*QBC35DRAFT_517514*	*Podospora australis*	PP-C1-A-PP-C2	Phe (Phe)	Penigainamide A / penigainamide B / penigainamide C / adametizine A / FA2097 / outovirin A / outovirin C / pretrichodermamide C

KAK4185106-A	*QBC35DRAFT_30052*	*Podospora australis*	A-PP-C1-PP-C2	NP (AMHOrn)	Metachelin C / metachelin A / metachelin A-CE / metachelin B / dimerumic acid 11-mannoside / dimerumic acid
KAK4187683-A	*QBC35DRAFT_452099*	*Podospora australis*	PP-E-C1-A-PP-C2	NP (NP)	NP
KAF2729070-A	*EJ04DRAFT_580912*	*Polyplosphaeria fusca*	PP-C1-A-PP-C2	Phe (Phe)	Aspirochlorine
KAF2729233-A	*EJ04DRAFT_447728*	*Polyplosphaeria fusca*	A-PP-C1-PP-PP-C2	Ala (HMMTyr)	Metachelin C / metachelin A / metachelin A-CE / metachelin B / dimerumic acid 11-mannoside / dimerumic acid
PGH15370-A	*AJ80_05554*	*Polytolypa hystricis*	PP-C1-A-PP-C2	Phe (6SMP acid)	Penigainamide A / penigainamide B / penigainamide C / adametizine A / FA2097 / outovirin A / outovirin C / pretrichodermamide C
PGH19362-A	*AJ80_04002*	*Polytolypa hystricis*	A-PP-C1-PP-C2	NP (AMHOrn)	Metachelin C / metachelin A / metachelin A-CE / metachelin B / dimerumic acid 11-mannoside / dimerumic acid
KAF2756230-A	*EJ05DRAFT_467526*	*Pseudovirgaria*	PP-C1-A-PP-C2	Phe (Phe)	NP
KAJ6440626-A	*O9K51_06416*	*Purpureocillium*	C1-A-PP-C2	NP (NP)	NP
KAJ6440810-A	*O9K51_06602*	*Purpureocillium lavendulum*	A-PP-C1-PP-C2	NP (AMHOrn)	Metachelin C / metachelin A / metachelin A-CE / metachelin B / dimerumic acid 11-mannoside / dimerumic acid
KAJ6445344-A	*O9K51_00103*	*Purpureocillium lavendulum*	PP-C1-A-PP-C2	Phe (Phe)	Aspirochlorine

GJN68316-A	*PLICBS_002359*	*Purpureocillium lilacinum*	A-PP-C1-PP-C2	NP (AMHOrn)	Metachelin C / metachelin A / metachelin A-CE / metachelin B / dimerumic acid 11-mannoside / dimerumic acid
GJN72625-A	*PLICBS_006700*	*Purpureocillium lilacinum*	PP-C1-A-PP-C2	Phe (Phe)	Aspirochlorine
GJN76426-A	*PLICBS_010539*	*Purpureocillium lilacinum*	C1-A-PP-C2	NP (NP)	NP
UNI23721-A	*JDV02_009523*	*Purpureocillium takamizusanense*	C1-A-PP-C2	Phe (Phe)	Aspirochlorine
TLD14182-A	*PgNI_04432*	*Pyricularia grisea*	A-PP-C1-PP-C2	NP (AMHOrn)	Metachelin C / metachelin A / metachelin A-CE / metachelin B / dimerumic acid 11-mannoside / dimerumic acid
TLD15771-A	*PgNI_02067*	*Pyricularia grisea*	C1-A-PP-C2	Gly (Gly)	Enniatin
TLD17678-A	*PgNI_00637*	*Pyricularia grisea*	PP-E-C1-A-PP-C2	NP (NP)	NP
QBZ55850-A	*PoMZ_00756*	*Pyricularia oryzae*	A-PP-C1-PP-C2	NP (AMHOrn)	Metachelin C / metachelin A / metachelin A-CE / metachelin B / dimerumic acid 11-mannoside / dimerumic acid
QBZ59770-A	*PoMZ_04734*	*Pyricularia oryzae*	PP-C1-A-PP-C2	Phe (Phe)	Penigainamide A / penigainamide B / penigainamide C / adametizine A / FA2097 / outovirin A / outovirin C / pretrichodermamide C
QBZ60755-A	*PoMZ_07697*	*Pyricularia oryzae*	PP-E-C1-A-PP-C2	NP (NP)	NP
CZT20576-A	*RCC_06434*	*Ramularia collo-cygni*	C1-A-PP-C2	Leu (leu)	NP

CZT21192-A	*RCC_07054*	*Ramularia collo-cygni*	C1-A-PP-C2	Phe (Phe)	Penigainamide A / penigainamide B / penigainamide C / adametizine A / FA2097 / outovirin A / outovirin C / pretrichodermamide C	T1PKS with DH, cMT, ER and KR domains
KIX07787-A	*Z518_02441*	*Rhinocladiella mackenziei*	A-PP-C1-PP-C2	NP (AMHOrn)	Metachelin C / metachelin A / metachelin A-CE / metachelin B / dimerumic acid 11-mannoside / dimerumic acid	
KIX09578-A	*Z518_00658*	*Rhinocladiella mackenziei*	PP-C1-A-PP-C2	Phe (Phe)	Penigainamide A / penigainamide B / penigainamide C / adametizine A / FA2097 / outovirin A / outovirin C / pretrichodermamide C	
KAH8168337-A	*LIA77_11601*	*Sarocladium implicatum*	E-C1-A-PP-C2	NP (NP)	NP	
KAH8168870-A	*LIA77_10996*	*Sarocladium implicatum*	A-PP-C1-PP-C2	NP (AMHOrn)	Metachelin C / metachelin A / metachelin A-CE / metachelin B / dimerumic acid 11-mannoside / dimerumic acid	
KAH8172387-A	*LIA77_06642*	*Sarocladium implicatum*	PP-C1-A-PP-C2	Phe (Phe)	Acetylaranotin	T1PKS with DH, ER and KR domains
KEZ45505-A	*SAPIO_CDS1828*	*Scedosporium apiospermum*	C1-A-PP-C2	Phe (Phe)	Aspirochlorine	T1PKS with DH, cMT, ER and KR domains
SAPIO_CDS2806p-A*	*SAPIO_CDS2806*	*Scedosporium apiospermum*	A-PP-C1-PP-C2	NP (AMHOrn)	NP	

ESZ92372-A	*SBOR_7227*	*Sclerotinia borealis*	ECH-A-PP-C1-PP-C2	NP (AMHOrn)	Metachelin C / metachelin A / metachelin A-CE / metachelin B / dimerumic acid 11-mannoside / dimerumic acid
ESZ95931-A	*SBOR_3744*	*Sclerotinia borealis*	PP-C1-A-PP-C2	Phe (Phe)	NP
EDN92698-A	*SS1G_08561*	*Sclerotinia sclerotiorum*	PP-C1-A-PP-C2	Phe (Phe)	NP
EDO01775-A	*SS1G_04250*	*Sclerotinia sclerotiorum*	A-PP-C1-PP-C2	NP (AMHOrn)	Metachelin C / metachelin A / metachelin A-CE / metachelin B / dimerumic acid 11-mannoside / dimerumic acid
CAD6448039-A	*SCLTRI_LOCUS7830*	*Sclerotinia trifoliorum*	A-PP-C1-PP-C2	NP (AMHOrn)	Metachelin C / metachelin A / metachelin A-CE / metachelin B / dimerumic acid 11-mannoside / dimerumic acid
CAD6448539-A	*SCLTRI_LOCUS8331*	*Sclerotinia trifoliorum*	PP-C1-A-PP-C2	Phe (Phe)	NP
KAH7303238-A	*B0I35DRAFT_498680*	*Stachybotrys elegans*	PP-C1-A-PP-C2	Phe (Phe)	Aspirochlorine
KAH7311724-A	*B0I35DRAFT_357393*	*Stachybotrys elegans*	A-PP-C1-PP-C2	NP (AMHOrn)	Metachelin C / metachelin A / metachelin A-CE / metachelin B / dimerumic acid 11-mannoside / dimerumic acid
KAH7312752-A	*B0I35DRAFT_411425*	*Stachybotrys elegans*	E-C1-A-PP-C2	Pro (NP)	NP
KAH7318045-A	*B0I35DRAFT_353657*	*Stachybotrys elegans*	C1-A-PP-C2	Phe (Phe)	NP
KAF7867404-A	*EAF04_005487*	*Stromatinia cepivora*	A-PP-C1-PP-C2	NP (AMHOrn)	Metachelin C / metachelin A / metachelin A-CE / metachelin B / dimerumic acid 11-mannoside / dimerumic acid
KAF7869782-A	*EAF04_004566*	*Stromatinia cepivora*	PP-C1-A-PP-C2	Phe (Phe)	NP
CRG92077-A	*PISL3812_09132*	*Talaromyces islandicus*	PP-C1-A-PP-C2	Phe (Phe)	NP

CRG92450-A	*PISL3812_09510*	*Talaromyces islandicus*	A-PP-C1-PP-C2	NP (R-beta-Phe)	NP	
CRG92456-A	*PISL3812_09516*	*Talaromyces islandicus*	A-PP-C1-PP-C2	NP (ILeu)	NP	
MCJ1325230-A	*MMC10_001892*	*Thelotrema lepadinum*	C1-A-PP-C2	Phe (Phe)	Penigainamide A / penigainamide B / penigainamide C / adametizine A / FA2097 / outovirin A / outovirin C / pretrichodermamide C	
MCJ1334644-A	*MMC10_011356*	*Thelotrema lepadinum*	C1-A-PP-C2	Phe (Phe)	NP	
TPX12938-A	*E0L32_006583*	*Thyridium curvatum*	C1-A-PP-C2	NP (NP)	NP	
TPX19017-A	*E0L32_011261*	*Thyridium curvatum*	C1-A-PP-C2	Phe (Phe)	Aspirochlorine	
TPX19125-A	*E0L32_011198*	*Thyridium curvatum*	A-PP-C1-PP-C2	NP (AMHOrn)	Metachelin C / metachelin A / metachelin A-CE / metachelin B / dimerumic acid 11-mannoside / dimerumic acid	
MCJ1247511-A	*MMC30_004725*	*Trapelia coarctata*	PP-C1-A-PP-C2	Phe (Phe)	Clapurines	
KAF2243876-A	*BU26DRAFT_436075*	*Trematosphaeria pertusa*	A-PP-C1-PP-PP-C2	NP (HMMTyr)	Metachelin C / metachelin A / metachelin A-CE / metachelin B / dimerumic acid 11-mannoside / dimerumic acid	
KAF2246847-A	*BU26DRAFT_576904*	*Trematosphaeria*	C1-A-PP-C2	Phe (Phe)	Aspirochlorine	
RFU77899-A	*TARUN_4297*	*Trichoderma arundinaceum*	C1-A-C2	Phe (Phe)	NP	
RFU78082-A	*TARUN_4143*	*Trichoderma arundinaceum*	PP-C1-A-PP-C2	NP (Ser)	Melinacidin IV	T1PKS with DH, cMT, ER and KR domains, and T1PKS with DH, ER and KR domains

RFU79859-A	*TARUN_2348*	*Trichoderma arundinaceum*	A-PP-C1-PP-C2	NP (AMHOrn)	Metachelin C / metachelin A / metachelin A-CE / metachelin B / dimerumic acid 11-mannoside / dimerumic acid
QYS97964-A	*H0G86_005166*	*Trichoderma simmonsii*	PP-C1-A-PP-C2	Phe (Phe)	Penigainamide A / penigainamide B / penigainamide C / adametizine A / FA2097 / outovirin A / outovirin C / pretrichodermamide C
QYS99530-A	*H0G86_006656*	*Trichoderma simmonsii*	PP-C1-A-PP-C2-PP	NP (Ser)	Melinacidin IV
QYT04173-A	*H0G86_011099*	*Trichoderma simmonsii*	A-PP-C1-PP-C2	NP (AMHOrn)	Metachelin C / metachelin A / metachelin A-CE / metachelin B / dimerumic acid 11-mannoside / dimerumic acid
QYT04980-A	*H0G86_011878*	*Trichoderma simmonsii*	A-PP-C1-PP-C2	NP (AMHOrn)	NP
EHK18682-A	*TRIVIDRAFT_44273*	*Trichoderma virens*	A-PP-C1-PP-C2	NP (AMHOrn)	Metachelin C / metachelin A / metachelin A-CE / metachelin B / dimerumic acid 11-mannoside / dimerumic acid
EHK21211-A	*TRIVIDRAFT_192365*	*Trichoderma virens*	A-PP-C1-PP-C2	NP (AMHOrn)	NP
EFE29754-A	*ARB_03095*	*Trichophyton*	PP-C1-A-PP-C2	Phe (Phe)	Aspirochlorine
EFE32737-A	*ARB_00195*	*Trichophyton*	A-PP-C1-PP-C2	Trp (Trp)	NP
EGE01737-A	*TEQG_00781*	*Trichophyton equinum*	A-PP-C1-PP-C2	Trp (Trp)	NP
EGE02744-A	*TEQG_01781*	*Trichophyton equinum*	C1-A-PP-E-C2-PP	NP (Arg)	NP
EGE04746-A	*TEQG_03919*	*Trichophyton equinum*	PP-C1-A-PP-C2	Phe (Phe)	Aspirochlorine
KAF3896673-A	*GY631_1970*	*Trichophyton*	PP-C1-A-PP-C2	Phe (Phe)	Aspirochlorine
KAG5204594-A	*GY631_7049*	*Trichophyton*	PP-C1-A-PP-C2	NP (2SMO)	NP
OAL64728-A	*A7C99_4162*	*Trichophyton rubrum*	PP-C1-A-PP-C2	Phe (Phe)	Aspirochlorine
EGD97620-A	*TESG_05025*	*Trichophyton tonsurans*	PP-C1-A-C2	Phe (Phe)	Aspirochlorine

EFE39655-A	*TRV_05651*	*Trichophyton*	A-PP-C1-PP-C2	Trp (Trp)	NP	
EFE43464-A	*TRV_01781*	*Trichophyton*	PP-C1-A-PP-PP-C2	Phe (Phe)	Acetylaranotin	
OAL70276-A	*A7D00_5242*	*Trichophyton violaceum*	PP-C1-A-PP-C2	Phe (Phe)	Aspirochlorine	
KAH6639924-A	*BKA67DRAFT_587913*	*Truncatella angustata*	PP-PP-C1-A-PP-C2	NP (Piperazic acid)	NP	
KAH6648185-A	*BKA67DRAFT_629600*	*Truncatella angustata*	C1-A-PP-C2	NP (Leu)	NP	
KAH6652741-A	*BKA67DRAFT_518577*	*Truncatella angustata*	PP-C1-A-PP-C2	Phe (2SADD acid)	NP	
KAH6657444-A	*BKA67DRAFT_673254*	*Truncatella angustata*	PP-C1-A-PP-C2	Phe (Phe)	Aspirochlorine	
KAH6657933-A	*BKA67DRAFT_533115*	*Truncatella angustata*	C1-A-PP-C2	Phe (Phe)	NP	
KAH6661027-A	*BKA67DRAFT_509664*	*Truncatella angustata*	A-PP-C1-PP-C2	NP (Trp)	Dimethylcoprogen	
KAI4125440-A	*LQ338_004243*	*Usnochroma*	PP-C1-A-PP-C2	Phe (Phe)	NP	
KAI3329769-A	*F4824DRAFT_377332*	*Ustulina deusta*	PP-C1-A-PP-C2	NP (NP)	NP	
KAI3330379-A	*F4824DRAFT_479012*	*Ustulina deusta*	A-PP-C1-PP-C2	NP (Trp)	Dimethylcoprogen	
KAI4150174-A	*LQ341_001162*	*Variospora aurantia*	C1-A-PP-C2	Phe (Phe)	Acetylaranotin	
KAI4154548-A	*LQ341_000331*	*Variospora aurantia*	C1-A-PP-C2	IVal (2-aminoisobutyric acid)	NP	
KAF2276642-A	*EI97DRAFT_432883*	*Westerdykella ornata*	C1-A-PP-C2	Phe (Phe)	Aspirochlorine	
KAF2276892-A	*EI97DRAFT_376305*	*Westerdykella ornata*	A-PP-C1-PP-PP-C2	NP (HMMTyr)	Metachelin C / metachelin A / metachelin A-CE / metachelin B / dimerumic acid 11-mannoside / dimerumic acid	
CAL8579366-A	*XPA_005116*	*Xanthoria parietina*	PP-C1-A-PP-C2	Phe (Phe)	NP	
KAI0454197-A	*F5B21DRAFT_504488*	*Xylaria acuta*	C1-A-PP-C2	NP (Arg)	NP	
KAI0455770-A	*F5B21DRAFT_181629*	*Xylaria acuta*	C1-A-PP-C2	Phe (Phe)	Gregatin A	T1PKS with DH, cMT and KR domains
KAI0456058-A	*F5B21DRAFT_523055*	*Xylaria acuta*	C1-A-PP-C2	Phe (Phe)	Acetylaranotin	
KAI0460296-A	*F5B21DRAFT_140310*	*Xylaria acuta*	A-PP-C1-PP-C2	NP (Trp)	Dimethylcoprogen	
KAI0966071-A	*F4678DRAFT_450681*	*Xylaria arbuscula*	PP-C1-A-PP-C2	NP (Piperazic acid)	NP	

KAI0967226-A	*F4678DRAFT_255703*	*Xylaria arbuscula*	A-PP-C1-PP-C2	NP (Trp)	Dimethylcoprogen	
KAI0970900-A	*F4678DRAFT_435040*	*Xylaria arbuscula*	PP-C1-A-PP-C2	Phe (Phe)	NP	
KAI0508614-A	*F5B22DRAFT_649909*	*Xylaria bambusicola*	C1-A-PP-C2	Phe (Phe)	Aspirochlorine	
KAI0508854-A	*F5B22DRAFT_378036*	*Xylaria bambusicola*	A-PP-C1-PP-C2	NP (Trp)	Dimethylcoprogen	
KAI0515171-A	*F5B22DRAFT_205661*	*Xylaria bambusicola*	PP-C1-A-PP-C2	NP (Piperazic acid)	NP	
KAI0517006-A	*F5B22DRAFT_142252*	*Xylaria bambusicola*	PP-C1-A-PP-C2	Phe (Phe)	Aspirochlorine	
KAI1746690-A	*F4782DRAFT_41571*	*Xylaria castorea*	A-PP-C1-PP-C2	NP (Trp)	Dimethylcoprogen	
KAI1750374-A	*F4782DRAFT_509491*	*Xylaria castorea*	C1-A-PP-C2	NP (SBHEA)	NP	
KAI1758248-A	*F4782DRAFT_544577*	*Xylaria castorea*	PP-C1-A-PP-C2	Phe (Phe)	NP	T1PKS with DH and KR domains
KAI0466119-A	*F4859DRAFT_338501*	*Xylaria cf. heliscus*	PP-C1-A-PP-C2	NP (Piperazic acid)	NP	
KAI0467726-A	*F4859DRAFT_231729*	*Xylaria cf. heliscus*	A-PP-C1-PP-C2	NP (Trp)	NP	
KAI0468885-A	*F4859DRAFT_188887*	*Xylaria cf. heliscus*	C1-A-PP-C2	Phe (Phe)	Gregatin A	T1PKS with DH, cMT and KR domains
KAI0856054-A	*F4860DRAFT_425507*	*Xylaria cubensis*	A-PP-C1-PP-C2	NP (Trp)	NP	
KAI0856113-A	*F4860DRAFT_494121*	*Xylaria cubensis*	PP-C1-A-PP-C2	NP (Phe)	Aspirochlorine	
KAI0859916-A	*F4860DRAFT_236272*	*Xylaria cubensis*	C1-A-PP-C2	NP (Piperazic acid)	NP	
KAI0545353-A	*F4679DRAFT_478540*	*Xylaria curta*	C1-A-PP-C2	NP (Piperazic acid)	NP	
KAI0549811-A	*F4679DRAFT_545657*	*Xylaria curta*	C1-A-PP-C2	Phe (Phe)	Aspirochlorine	
KAI0551290-A	*F4679DRAFT_582767*	*Xylaria curta*	A-PP-C1-PP-C2	NP (Trp)	NP	
KAI0532277-A	*GGR58DRAFT_490818*	*Xylaria digitata*	A-PP-C1-PP-C2	NP (Trp)	Dimethylcoprogen	
KAI0534854-A	*GGR58DRAFT_504887*	*Xylaria digitata*	PP-C1-A-PP-C2	NP (Piperazic acid)	NP	
KAI0540238-A	*GGR58DRAFT_499439*	*Xylaria digitata*	A-PP-C1-C2	NP (Trp)	NP	
TRX90974-A	*FHL15_008179*	*Xylaria flabelliformis*	PP-C1-A-PP-C2	NP (Phe)	Aspirochlorine	
TRX91033-A	*FHL15_008015*	*Xylaria flabelliformis*	C1-A-PP-C2	NP (Piperazic acid)	NP	
TRX93540-A	*FHL15_005512*	*Xylaria flabelliformis*	A-PP-C1-PP-C2	NP (Trp)	NP	
RWA06478-A	*EKO27_g8628*	*Xylaria grammica*	PP-C1-A-PP-C2	Phe (Phe)	NP	

RWA07639-A	*EKO27_g7466*	*Xylaria grammica*	PP-C1-A-PP-C2	NP (Piperazic acid)	NP
RWA07771-A	*EKO27_g7339*	*Xylaria grammica*	A-PP-C1-PP-C2	NP (Arg)	Dimethylcoprogen
RWA08673-A	*EKO27_g6431*	*Xylaria grammica*	C1-A-PP-C2	Phe (Phe)	Aspirochlorine
RWA11889-A	*EKO27_g3185*	*Xylaria grammica*	PP-C1-A-PP-C2	NP (Ala)	NP
TGJ76594-A	*E0Z10_g10864*	*Xylaria hypoxylon*	A-PP-C1-C2	NP (NCPAla)	NP
TGJ80893-A	*E0Z10_g7887*	*Xylaria hypoxylon*	PP-C1-A-PP-C2	Phe (Phe)	NP
TGJ82397-A	*E0Z10_g6365*	*Xylaria hypoxylon*	PP-C1-A-PP-C2	NP (Piperazic acid)	NP
KAI1820251-A	*F4861DRAFT_75428*	*Xylaria intraflava*	PP-C1-A-PP-C2	Phe (Phe)	NP
KAI1823954-A	*F4861DRAFT_539556*	*Xylaria intraflava*	C1-A-PP-C2	NP (Piperazic acid)	NP
KAI1824916-A	*F4861DRAFT_224639*	*Xylaria intraflava*	PP-C1-A-PP-C2	Phe (Phe)	Penigainamide A / penigainamide B / penigainamide C / adametizine A / FA2097 / outovirin A / outovirin C / pretrichodermamide C
KAI1825838-A	*F4861DRAFT_500026*	*Xylaria intraflava*	PP-C1-A-PP-C2	Phe (Phe)	NP
KAI8943992-A	*F4801DRAFT_573266*	*Xylaria longipes*	PP-C1-A-PP-C2	NP (Piperazic acid)	NP
KAI8946985-A	*F4801DRAFT_30349*	*Xylaria longipes*	PP-C1-A-PP-C2	Phe (Phe)	NP
KAI8953066-A	*F4801DRAFT_153683*	*Xylaria longipes*	A-PP-C1-PP-C2	NP (Trp)	Dimethylcoprogen
KAF2963041-A	*GQX73_g10533*	*Xylaria multiplex*	A-PP-C1-PP-C2	NP (Trp)	Dimethylcoprogen
KAF2970581-A	*GQX73_g3008*	*Xylaria multiplex*	PP-C1-A-PP-C2	NP (Piperazic acid)	NP
KAF2973016-A	*GQX73_g509*	*Xylaria multiplex*	C1-A-PP-C2	Phe (Phe)	Penigainamide A / penigainamide B / penigainamide C / adametizine A / FA2097 / outovirin A / outovirin C / pretrichodermamide C
KAI0399994-A	*F4802DRAFT_514907*	*Xylaria palmicola*	PP-C1-A-PP-C2	NP (Piperazic acid)	NP

KAI0401758-A	*F4802DRAFT_579052*	*Xylaria palmicola*	C1-A-PP-C2	Phe (Phe)	NP	
KAI0402334-A	*F4802DRAFT_576027*	*Xylaria palmicola*	PP-C1-A-PP-C2	Phe (Phe)	Aspirochlorine	
KAI0403809-A	*F4802DRAFT_279302*	*Xylaria palmicola*	A-PP-C1-PP-C2	NP (Trp)	Dimethylcoprogen	
KAH8160861-A	*CIB48_g7393*	*Xylaria polymorpha*	PP-C1-A-PP-C2	Phe (Phe)	NP	
KAH8160895-A	*CIB48_g7352*	*Xylaria polymorpha*	C1-A-PP-C2	Phe (Phe)	Aspirochlorine	
KAH8166011-A	*CIB48_g2253*	*Xylaria polymorpha*	A-PP-C1-PP-C2	NP (Phe)	Dimethylcoprogen	
KAI0442943-A	*F4803DRAFT_342885*	*Xylaria telfairii*	A-PP-C1-PP-C2	NP (Trp)	Dimethylcoprogen	
KAI0444032-A	*F4803DRAFT_513124*	*Xylaria telfairii*	C1-A-PP-C2	NP (Piperazic acid)	NP	
KAI0445790-A	*F4803DRAFT_506436*	*Xylaria telfairii*	C1-A-PP-C2	Phe (Phe)	Gregatin A	T1PKS with DH, cMT and KR domains
KAI0447487-A	*F4803DRAFT_574690*	*Xylaria telfairii*	PP-C1-A-PP-C2	Phe (Phe)	NP	
MCJ1380199-A	*MMC17_003302*	*Xylographa soralifera*	PP-C1-A-PP-C2	Phe (Phe)	Aspirochlorine	
MCJ1401062-A	*MMC11_004274*	*Xylographa trunciseda*	PP-C1-A-PP-C2	Phe (Phe)	Aspirochlorine	
KAJ2904207-A	*MKZ38_008537*	*Zalerion maritima*	PP-C1-A-PP-C2	Phe (Phe)	NP	
KAJ2904358-A	*MKZ38_008274*	*Zalerion maritima*	PP-E-C1-A-PP-C2	NP (NP)	NP	
KAJ2904839-A	*MKZ38_007031*	*Zalerion maritima*	A-PP-C1-PP-C2	NP (Trp)	Metachelin C / metachelin A / metachelin A-CE / metachelin B / dimerumic acid 11-mannoside / dimerumic acid	
KAF2179233-A	*K469DRAFT_674382*	*Zopfia rhizophila*	PP-C1-A-PP-C2	Phe (Phe)	Aspirochlorine	
KAF2192164-A	*K469DRAFT_620172*	*Zopfia rhizophila*	PP-C1-A-PP-C2	Phe (Phe)	NP	
KAF2193542-A	*K469DRAFT_551298*	*Zopfia rhizophila*	A-PP-C1-PP-C2	Ala (2SADD acid)	Metachelin C / metachelin A / metachelin A-CE / metachelin B / dimerumic acid 11-mannoside / dimerumic acid	
KJX98398-A	*TI39_contig413g00001*	*Zymoseptoria brevis*	C1-A-PP-C2	Phe (Phe)	NP	T1PKS with DH, cMT, ER and KR domains

EGP89696-A	*MYCGRDRAFT_19958*	*Zymoseptoria tritici*	C1-A-PP-C2	Phe (Phe)	Acetylaranotin	T1PKS with DH, cMT, ER and KR domains

*Not identified by anti-SMASH as the corresponding gene was erroneously considered as a pseudogene during the automated annotation of the genome using Augustus.

A: adenylation domain; C: condensation domain; NP: no prediction. PP: phosphopantetheine (for thiolation domain).
2SADD acid: 2S-aminododecanoic acid; 2SHI acid: 2S-hydroxyisovaleric acid; 2SMO: 2S-methyl-3-oxobutyrine; 3SMAsp acid: 3S-methyl-D-aspartic acid branched; 4SDHAP acid: 4,S5-dihydroxy-2S-aminopentanoic acid; 6SMP acid: 6S-methyl-pipecolic acid; AMHOrn: N5-cis-anhydromevalonyl-N5-hydroxyornithine; AMHOrn*: N5-trans-anhydromevalonyl-N5-hydroxyornithine; HMMTyr: 3-hydroxy-O-methyl-5-methyltyrosine; NCPAla: 3-(2-nitrocyclopropylalanine); PAB acid: Para-aminobenzoic acid; PC acid: 1-pyrroline-5-carboxylic acid; SBHEA: S-beta-hydroxyenduracididine; SHA acid: Succinyl-hydrazinoacetic acid.

Supplementary Table S3: The amino acid sequence of the acyl carrier protein domain (PP domain) of selected PKSs allowing the assembly of a well-defined number of condensation units, and of KEZ45498 and its orthologs.

Protein Genbank accession number	Gene GenBank accession number	Fungal species and strain	Metabolite produced	Amino acid sequence of the acyl-carrier protein domain (PP domain)	Number of malonyl-CoA units
BAJ09789-PP	*AB514562.1* (*Sol1*)	*Alternaria solani*	Solanapyrone	AERSRTVALVTDAIVKTVAQMLFVDASGVDASRTVADYGVDSLIAAELRNWFNVAFGADVSMLEMLDTAT	7
KAI4646669-PP	*J4E93_004891*	*Alternaria ventricosa* BMP 2768	Solanapyrone	ALALVTSAISNAVATMLLLDPATVNCARAVSDHGVDSLIAAELRNWFHVALGYKVSMVDLLDSRMSIAALAERIVR	7
KAF9696274-PP	*EKO04_005193*	*Ascochyta lentis* AI4	Mellein	LSRVLHLDVEEIDDRAAIADLGVDSVMTVALRQQLQKAMGITVPPTLTWNHPTVGHLVE	4
KAF9700418-PP	*EKO04_001183*	*Ascochyta lentis* AI4	Monacolin K	QAAISESLSDKMRGVLHIPPEESVNADAPLLDQGVDSLGAITVASWFSKQLLVEIPILRILSGASINDLAAEGASR	8
RAH67332-PP	*BO66DRAFT_458223*	*Aspergillus aculeatinus* CBS 121060	Monacolin K	ILDGLSGRIRGALQLTAADELNLTVPLIDQGVDSLSAVTVGSWFTKNLSIDIPLLKILGGASVADLIDEAVSR	8
KAF7175177-PP	*CNMCM7691_006581*	*Aspergillus felis* CNM-CM7691	Fumagillin	RAAAITVVGARLAKQLRLTDALDSARPLSYYGLDSLAAVELRSWVRMTLGIELTTLDVMNAASLGELCDKA	5
KAF7178407-PP	*CNMCM7691_007098*	*Aspergillus felis* CNM-CM7691	Monacolin K	RIRGALQLTPADELNLTVPLIDQGVDSLSAVTVGSWFTKNLNIDIPLLKILGGASVTDLVDEAVSR	8
RAK77236-PP	*BO72DRAFT_527717*	*Aspergillus fijiensis* CBS 313.89	Monacolin K	ILDGLSGRIRGALQLTAADELNLTVPLIDQGVDSLSAVTVGSWFTKNLSIEIPLLKILGGASAADLIDEAVSR	8
KAF4239406-PP	*CNMCM6805_005828*	*Aspergillus fumigatiaffinis* CNM-CM6805	Fumagillin	DRAAVRAAAITVVGARLAKQLRLTDALDSARPLSYYGLDSLAAVELRNWVRMTLAIELTTLDVINAASVGELCDKMV	5
EAL85129-PP	*AFUA_8G00370*	*Aspergillus fumigatus* Af293	Fumagillin	RAALRAAAITVVGARLAKQLRLTDAVDPARPLSYYGLDSLAAVELRTWVRMTLAIELTTLDVMNAASLGELCEKV	5

EAA65604-PP	*AN1036.2*	*Aspergillus nidulans* FGSC A4	Asperfuranone	NYVGGAIATKLADIFMVPVADIDLTKPPSAYGVDSLVAVELRNMLVLQAACDVSIFSILQSVSLAALAGMVVEK	3
EAU29410-PP	*ATEG_09961*	*Aspergillus terreus* NIH2624	Monacolin K	TNLDQGVDSLGAVTVGTWFSKQLYLDLPLLKVLGGASIADLADEAAARL	8
EAU31921-PP	*ATEG_07659*	*Aspergillus terreus* NIH2624	Asperfuranone	ESSDAAARCVGDAIATKLADIFMVPVDDIDLSKPPSAYGVDSLVAVELRNMLVLQAACDVSIFSILQSASLAALALDVVA	3
GIC92256-PP	*Aud_008722*	*Aspergillus udagawae* IFM 46973	Fumagillin	DRAAVRAAAITVVGARLAKQLRLTDALDSARPLSYYGLDSLAAVELRNWVRMTLGIELTTLDVMNAASLGELCDKVIG	5
GIK06546-PP	*Aspvir_002196*	*Aspergillus viridinutans* IFM 47045	Fumagillin	RAVAITVVGARLAKQLRITDALDSARPLSYYGLDSLAAVELRSWVRMTLGIELTTLDVMNAASLGELCDKV	5
KAF4303810-PP	*GTA08_BOTSDO08126*	*Botryosphaeria dothidea* sdau11-99	Mellein	RPAGGPELKAWLNVKIRECIAAVLMIGDIEEVDPRAAVSDLGVDSVMTVALRQKLQAGMGIKVPPTLTWNHPTVVHLVEWF	4
KAF1957322-PP	*CC80DRAFT_503688*	*Byssothecium circinans* CBS 675.92	Mellein	EKVKGCLSTVLHLDVEEIDDRAAIADLGVDSVMSVGLRMEMQKTLGIKVPPSLLWKEDTVARLVDWVCGQL	4
KAI4289203-PP	*L6R35_001529*	*Caloplaca aegaea* LIQ143CAAG	Solanapyrone	EKAKTITLLTGAIVTAVAEMLFIDGSGINPVRTVADLGVDSLIAAELRNWFLVALGYDISMLDLLDAHTSINALAANIVE	7
KAK0671960-PP	*QBC41DRAFT_344286*	*Cercophora samala* CBS 307.81	Solanapyrone	AAARAATVGLVTEGVTQTVAGMLFIDASMVDPAKSIAEHGVDSLIAAELRSWFHQALKTNLKMGELLDAQTSIRALAENI	7
KAK3291710-PP	*B0H64DRAFT_329829*	*Chaetomium fimeti* CBS 168.71	Solanapyrone	VESAITNAVPEMLFVDVESIDPAKSVADLGVDSLIAAELRNWFLQALGTNISMLDLLD	7
KAK3293714-PP	*B0H64DRAFT_434836*	*Chaetomium fimeti* CBS 168.71	Solanapyrone	AVVLVTGGVCSAIAEMLFIDVGNVNPGKSVAEHGVDSLIAAELRNWFLQALRADMRNLLDSTLSIRALAE	7
KAK5994069-PP	*PT974_07509*	*Cladobotryum mycophilum* ATHUM6906	Solanapyrone	AQDAIVNIVAEMLFVDVESIDAAKSVADLGVDSLIAAELRNWFRQALGATISMLNLLD	7
KAK7222205-PP	*V2G26_010208*	*Clonostachys chloroleuca* Cc878	Solanapyrone	AEDLAAAAIASTVAQMVFIDAAQVSPSRSVSDYGVDSLIAAELRHWFSLAFGSDISMLELLDTKRSIKSIAKTLV	7

CAH0025332-PP	*CRHIZ90672A_00008122*	*Clonostachys rhizophaga*	Solanapyrone	AEDLAAAAIASTVAQMVFIDAAQVSPSRSVSDYGVDSLIAAELRHWFSLAFGSDISMLELLDTKRSIKSIAKTLV	7
VUC28461-PP	*CLO192961_LOCUS236010*	*Clonostachys rosea*	Solanapyrone	EDLAAATISSTVAQMVFIDAAQVSPSRSVSDYGVDSLIAAELRHWFSLAFGSDISMLELLDTKRSIKSIAKTLV	7
RDW74769-PP	*BP6252_05911*	*Coleophoma cylindrospora* BP6	Betaenone	FAAQLRNILQMTMADDDLMEMRSNDIGLDSLVSVDIRTWFLKNYQVSIPVLKIMGNDTMGNLAQHALE	7
KAF0320406-PP	*GQ607_012332*	*Colletotrichum asianum* ICMP 18580	Betaenone	IQESFSAQLRSILQISTPDDDLMVMRSVNLGLDSLVSVDIRSWFLKNFQVSIPVLKIMSND	7
KAK2054714-PP	*LY76DRAFT_638245*	*Colletotrichum caudatum* CBS 131602	Boydins	QELARRILEALAQELRRMFGSADVDVSRDLTAHGVDSLVAVEIRNWLAAQTGVGLSVLDLIQAPSLTDLADCVAER	
KAJ0365225-PP	*COL26b_012164*	*Colletotrichum chrysophilum* AFK26	Betaenone	IQESFSAQLRSILQISTPDDDLMVMRSVNLGLDSLVSVDIRSWFLKNFQVSIPVLKIMSND	7
KAJ0367107-PP	*COL26b_011392*	*Colletotrichum chrysophilum* AFK26	Solanapyrone	ELREKALEFVTASISGTVATMLLLDESAINASRAVSEHGVDSLIAAELRSWFHIALGSKISMVDLLDPRTSINALAAKV	7
Ccliv733p-PP	*JANWGG010000008.1:2756602-2766301*	*Colletotrichum cliviicola* YN31 yn31-8	Boydins	LARRIMEALAQELRRMFSSADVDMSRDLTAHGVDSLVAVEIRNWLAAQTGISL	
KAF4479090-PP	*CGGC5_v012906*	*Colletotrichum fructicola* Nara gc5	Betaenone	IQESFSAQLRSILQISTPDDDLMVMRSVNLGLDSLVSVDIRSWFLKNFQVSIPVLKIMSN	7
KAF4487404-PP	*CGGC5_v005066*	*Colletotrichum fructicola* Nara gc5	Solanapyrone	ELREKALEFVTASISGTVATMLLLDESAINATRAVSEHGVDSLIAAELRSWFHIALGSKISMVDLLDPRTSINALAAKV	7
KAF3799748-PP	*GCG54_00000994*	*Colletotrichum gloeosporioides* Lc1	Betaenone	QESFSAQLRSILQISTTDDDLMVMRSVNLGLDSLVSVDIRSWFLKNFQVSIPVLKIMSND	7
KAF3806818-PP	*GCG54_00007067*	*Colletotrichum gloeosporioides* Lc1	Solanapyrone	ALAQGAIVKAVAEMLFVDAESIDPAKSVADLGVDSLIAAELRNWFLQALGANISMLDLLD	7
KAF3809602-PP	*GCG54_00012888*	*Colletotrichum gloeosporioides* Lc1	Solanapyrone	ELREKALEFVMASISGTVATMLLLDESAINAARAVSEHGVDSLIATELRSWFHIALGSKISMVDLLDPRTSINALAAKV	7
UQC80483-PP	*CLUP02_05966*	*Colletotrichum lupini* IMI 504893	Monacolin K	DEVRDIIIDGLSQKMRGILHIPADEKVNATTPLIDQGVDSLGAITVGSWFSKTLMIDIPLLRVVSGASIAELAEEAAGRL	8

KAF6838975-PP	*CMUS01_04436*	*Colletotrichum musicola* LFN0074	Boydins	LREALPRDLGPKKLARRILEALAQELRRMFSSADVDVSRDLTAHGVDSLVAVEIRNWLAAQTGIGLSVLDLIQAPSLTDLADCVGRR	
KAJ0314002-PP	*Brms1b_007328*	*Colletotrichum noveboracense* PMBrms-1	Solanapyrone	ELREKALEFVTASISGTVATMLLLDESAINASRAVSEHGVDSLIAAELRSWFHIALGSKISMVDLLDPRTSINALAAKV	7
KAJ0281340-PP	*CBS470a_008332*	*Colletotrichum nupharicola* CBS470	Solanapyrone	ELREKALEFVTASISGTVATMLLLDESAINASRAVSEHGVDSLIAAELRSWFHIALGSKISMVDLLDPRTSINALAAKV	7
TDZ17454-PP	*Cob_v009566*	*Colletotrichum orbiculare* MAFF 240422	Monacolin K	EVRGIIADGLSQKMRGILHIPADEQVDAAAPLIDQGVDSLGAITVGSWFSKTLMIDMPLLRVVSGASIAELADEAAGRL	8
KAF5491715-PP	*CGCS363_v010439*	*Colletotrichum siamense* Cg363	Betaenone	RVIQESFSAQLRSILQISTPDDDLMVMRSVNLGLDSLVSVDIRSWFLKNFQVSIPVLKIMSNES	7
KAF5496806-PP	*CGCS363_v007959*	*Colletotrichum siamense* Cg363	Solanapyrone	ELREKALEFVTASISGTVATMLLLDESAINASRAVSEHGVDSLIAAELRSWFHNALGSKISMVDLLDPRTSINALAAKV	7
TDZ32535-PP	*C8035_v011561*	*Colletotrichum spinosum* CBS 515.97	Monacolin K	EVRGIIADGLSQKMRGILHIPADEQVDAAAPLIDQGVDSLGAITVGSWFPKTLMIDMPLLRVVSGASIAELADEAAGRL	8
TDZ67308-PP	*CTRI78_v002929*	*Colletotrichum trifolii* 543-2	Monacolin K	ATTVQEVRRIIADGLSQKMRGILHIPADEQVDAAAPLIDQGVDSLGAITVGSWFSKTLMIDMPLLRVVSGASIAELADEAAGRL	8
KAK2603556-PP	*QQS21_004236*	*Conoideocrella luteorostrata* ARSEF 14590	Betaenone	TAANSEKVIREAFAALLRQELQITDKEDAELMKMRSNEIGLDSLVSVDIRSWFLKATGVNIPVMRILSHNNMESLVQLAAR	7
KAF1843183-PP	*K460DRAFT_387332*	*Cucurbitaria berberidis* CBS 394.84	Solanapyrone	SKDIGEATLLVTKSIIARMADMLFISAEGIDATKGVAAYGMDSLIAAELRNWFVSTYKCTISFLKLLDTATSITELADIV	7
KAF1848288-PP	*K460DRAFT_414864*	*Cucurbitaria berberidis* CBS 394.84	Monacolin K	KAVTIEEVRQIITDGVSGKIRVALQVAADDPISITTPLIDEGVDSLSAVTIGAWFSKNLDIDIPLLKILGGASVTDLVEEVIERL	8
KAK7734288-PP	*SLS53_007938*	*Cytospora paraplurivora* FDS-564	Solanapyrone	AETLAFVTEAIVTAVAEILFIDASGVDPARPVAAYGVDSLIAAELRNWFHVALGSDIRTLDLLDAHMSIAALAVD	7

KAK7739675-PP	*SLS53_005642*	*Cytospora paraplurivora* FDS-564	Betaenone	IKRAFAAQLRNILQVTTSDDDLMASRSSEIGLDS LVSVDIRSWFLKALGVSIPVLQIMGNDTMSNLVQ HAAE	7
KAI1799436-PP	*F4811DRAFT_568286*	*Daldinia bambusicola* CBS 122872	Solanapyrone	SKTQALVTSAVAATVAQVLFIDVSGVDTNRTVAD YGVDSLIAAELRNWFNTAFGADISMLDLLDTRT	7
KAI1462952-PP	*F4812DRAFT_469679*	*Daldinia caldariorum* CBS 122874	Solanapyrone	NKTHALVTSAVVATVAQVLFIDVSGVDANRTVAD YGVDSLIAAELRNWFNTAFGADISMLDLLDTRT	7
KAF3064415-PP	*GL218_01327*	*Daldinia childiae* JS-1345	Solanapyrone	NKTQALVTSAVVATVAQVLFIDVSGVDANRTVAD YGVDSLIAAELRNWFNTAFGADISMLDLLDTRT	7
KAI1661868-PP	*F4813DRAFT_175408*	*Daldinia decipiens* CBS 113046	Solanapyrone	NKTQALVTSAVVATVAQVLFIDVSGVDANRTVAD YGVDSLIAAELRNWFNTAFGADISMLDLLDTRT	7
KAK6950575-PP	*Daesc_008903*	*Daldinia eschscholtzii* MFLUCC 19-0629	Solanapyrone	NKTQALVTSAVVATVAQVLFIDVSGVDANRTVAD YGVDSLIAAELRNWFNTAFGADISMLDLLDTRT	7
KAI0115381-PP	*F4814DRAFT_449214*	*Daldinia grandis* CBS 11473	Solanapyrone	NKTQALVTSAVVATVAQVLFIDVSGVDANRTVAD YGVDSLIAAELRNWFNTAFGADISMLDLLDTRT	7
KAI1647504-PP	*F4817DRAFT_315683*	*Daldinia loculata* CBS 113971	Solanapyrone	NKTQALVTSAVVATVAQVLFIDVSGVDANRTVAD YGVDSLIAAELRNWFNTAFGADISMLDLLDTRT	7
KAI0843956-PP	*F5Y00DRAFT_256398*	*Daldinia vernicosa* CBS 139.73	Solanapyrone	APERAKIVGFVTSAISTAVAEMLFVDVSNTNPGR SVADHGVDSLITAELRNWFHQALGAKINTLELLD ARLSIAALADRIV	7
KAI0846075-PP	*F5Y00DRAFT_272316*	*Daldinia vernicosa* CBS 139.73	Solanapyrone	NKTQALVTSAVVATVAQVLFIDVSGVDANRTVAD YGVDSLIAAELRNWFNTAFGADISMLDLLDTRT	7
KAJ0117744-PP	*J7T55_001944*	*Diaporthe amygdali* CAA958	Betaenone	AKEGLAAQLRTILQLSTADDDLMSMRSVDLGLDS LVSVDIRSWFLKNFEVSIPVLKIMA	7
KAI7776540-PP	*LA080_004798*	*Diaporthe eres* CBS 160.32	Mellein	GPEFKEWLNVTIRECLAAVTMGEVDEIDPRAPMS DLGVDSVMTVVLSQKLQAAMGIKVPVTLTWNHPT IL	4

KAI7777429-PP	*LA080_003524*	*Diaporthe eres* CBS 160.32	Betaenone	ELLKIIKDTFAAQLRNVLQMSTPDEDLMAMRSSEIGLDSLISVDVRAWFLKHYHVSVPVLKIMGNDTMVNIAEHVAEN	7
POS74005-PP	*DHEL01_v207603*	*Diaporthe helianthi* 7/96	Solanapyrone	EKVVGFVVDAITKAVAEMLFIDVVSINPANSVAGHGVDSLIAAELRSWFHQALGHGSIKTQELLDTGKSITQLARTV	7
KAJ4354064-PP	*N0V89_005797*	*Didymosphaeria variabile* MI 356815	Mellein	IRECIGAVLHMDVEDIDVRAAIADLGVDSVMTVSLRQKLLSVLGVKVPPTLLWKEPTVGHLVEW	4
OJD30788-PP	*BKCO1_5600057*	*Diplodia corticola* CBS 112549	Mellein	IRECIGAVLMMNDIEDIDPRAAVADLGVDSVMTVALRTKLQQAMSIKVPPTLTWNHPTVVHLVEW	4
KAL1640659-PP	*SLS58_006673*	*Diplodia intermedia* M45-28	Mellein	IRECIGAVLMMNDIDDIDPRAAVADLGVDSVMTVALRTKLQQAMAIKVPPTLTWNHPTVVHLVEW	4
KAF2128650-PP	*P153DRAFT_423820*	*Dothidotthia symphoricarpi* CBS 119687	Mellein	IRECLCAVLHLDIEDIDDRAAIADLGVDSVMTVSLRQQLQKVMGIKVPPTLTWSHPTVIHLIDW	4
KAF2129141-PP	*P153DRAFT_292080*	*Dothidotthia symphoricarpi* CBS 119687	Betaenone	TLQVVQQAFGAQLRRILQVSTSDDDLMMMRGVDLGFDSLLSVDVRSWFLKNFQVSIPVLKIM	7
KAI6780255-PP	*J7T54_003034*	*Emericellopsis cladophorae* MUM 19.33	Mellein	EGLRQWLEVSVRQCIAAVLMMPDITEIDGGTMVNDLGIDSVMTVALRRQFQEFFQIKVPPTLTWKHPTVDSMVPWFAA	4
OSS45548-PP	*B5807_10259*	*Epicoccum nigrum* ICMP 19927	Mellein	SRVLYMDVDDIEDRVAIADLGVDSVMTVALRQQLQKTMGVTVPPTLTWNHPTVAHLVE	4
KAF5718368-PP	*FGLOB1_1668*	*Fusarium globosum* NRRL 26131	Solanapyrone	VELVTGAISKALSDMLFIDILNINPANSIAGHGVDSLIAAELRNWFHQAFGISIMTGQLLDADTGIKDLA	7
KAH0960594-PP	*HRG_08749*	*Hirsutella rhossiliensis* HR02	Monacolin K	SRICGALQLASADELNLITPLIDLGVDSLSAVTIGSWFSKNLSIDIPLLKILGGASVNDLADEAVSR	8
KAH0964015-PP	*HRG_04443*	*Hirsutella rhossiliensis* HR02	Solanapyrone	ASAVALVQSAIINVVAEMLFVDVESIDPAKSVADLGVDSLIAAELRNWFLLGLGTNISMLDLLD	7
KAH0964812-PP	*HRG_02828*	*Hirsutella rhossiliensis* HR02	Betaenone	IKEALGTQLRKALLVSTSDDDLMRMGGVELGFDSLVAVDLRTWMIKNFQANIPVLKMM	7
KAI0868438-PP	*GGS24DRAFT_506785*	*Hypoxylon argillaceum* CBS 527.63	Solanapyrone	TTQMAQRIIIAAVAEMLFVDVESIDPAKSVAELGVDSLIAAELRSWFVQALGARISMLDLLD	7
KAI1773027-PP	*F4818DRAFT_453383*	*Hypoxylon cercidicola* CBS 119009	Solanapyrone	VQGAIINMVAEMLFVDIESIDPAKSMADLGVDSLVAAELRNWFLQALGANISMLDLL	7

KAI1398297-PP	*F4819DRAFT_489710*	*Hypoxylon fuscum* CBS 119018	Solanapyrone	LALVTGGVTTAVAEMLFIEESGVNLGKTVADHGVDSLIAAELRNWFHVALGYNITMLDLLDAHTSINALAAKI	7
KAI1405297-PP	*F4819DRAFT_501936*	*Hypoxylon fuscum* CBS 119018	Solanapyrone	PTTVELVTDGISKAIAEMLFIDAGNVNISKSVAEHGVDSLIAAELRNWFHQALNADLRNLL	7
KAK0617489-PP	*B0T14DRAFT_484399*	*Immersiella caudata* CBS 606.72	Boydins	ILGELSKELMKMFGTQEVPDAGKDLTAHGVDSLVAVEIRNWFAAQAGVEFSILDLIQSPSLTHLAGVAAARL	
KAI1100574-PP	*F4804DRAFT_348396*	*Jackrogersella minutella* CBS 135445	Betaenone	QVVERAFATQLRVVLQVTTSDHDLMASRSSEIGLDSLVSLDIRSWFLKSLQVNIPVLKIMGNNAMTSLVDYAIERL	7
KAK0609813-PP	*DIS24_g12234*	*Lasiodiplodia hormozganensis* CBS 339.90	Mellein	IRECIGAVLMMNDIEDIDPRAAVSDLGVDSVMTVALRQKLQAAMAPVKVPPTLTWNHPTVVHLVEW	4
KAJ8130860-PP	*O1611_g2768*	*Lasiodiplodia mahajangana* VT137	Solanapyrone	AQRIIVAAVAEMLFVDAESIDPAKSVAELGVDSLIAAELRSWFVQALGADISMLDLLD	7
KAJ8131214-PP	*O1611_g2411*	*Lasiodiplodia mahajangana* VT137	Solanapyrone	VTNATTTAVAETLFVDVSTVNPARSVAEHGVDSLIAAELRNWFHQALEANITMQELLDAKTS	7
KAF4545462-PP	*LTHEOB_5295*	*Lasiodiplodia theobromae* AM2As	Mellein	IRECIGAVLMMNDIEDIDPRAAVSDLGVDSVMTVALRQKLQAAMAPVKVPPTLTWNHPTVVHLVEW	4
KAF2680119-PP	*K458DRAFT_479964*	*Lentithecium fluviatile* CBS 122367	Mellein	KSGPELKEYITAQVKQCLSTVLHLDVEDIDTRAVVADLGVDSVMMGALRWEMQKVLGLKVPPTLVWKAGTVVVLVDWA	4
KAF2685876-PP	*K458DRAFT_300205*	*Lentithecium fluviatile* CBS 122367	Mellein	ELKTWLDVKIRECIAFVMKICDIEEIDPRIPLSDFGVDSVMTIALRQKLQNSLKVKVPQTLTWNHPTVIAMVEWFLK	4
KAK3178717-PP	*OEA41_000854*	*Lepraria neglecta* Allen 5258	Solanapyrone	RATTVELVTDGIAKTIAEMLFIDAGNVNPSKSVAEHGVDSLIAAELRNWFHQALRTKLQNLLDSQTSIKMLAEQI	7
KAF1351738-PP	*EJ07DRAFT_159267*	*Lizonia empirigonia* CBS 542.76	Mellein	KSGPELKAHLNAEIKGCVSKVLHLDVEEIDDRAAIADLGVDSVMTVALRQQLQKAMGITVPPTLTWNHPTVGHLVEW	4
MCJ1265005-PP	*MMC22_004880*	*Lobaria immixta* Werth ST12-03b	Betaenone	KQAFAAQLCRILQTSMADVNLMSMRGVDLGLDSLISVDLRSWFMKIFQVSIPVLKIMA	7
KAF2265025-PP	*CC78DRAFT_616181*	*Lojkania enalia* CBS 304.66	Solanapyrone	DLIADAVMQRTATLLMISFENMEAGKSIAEYGVDSLIAVELRNWIAVTWETNVPLLVLLDERLSIRKLADG	7

KAF2107588-PP	*BDV96DRAFT_653667*	*Lophiotrema nucula* CBS 627.86	Mellein	TKVRESLGAVLHITDLEDIDVRSAVSDLGVDSVMTVSLRQKLQSVLGVKVPPTLTWNCPTIGHLVE	4
EKG12695-PP	*MPH_10194*	*Macrophomina phaseolina* MS6	Mellein	IRECIAAVLMISDIEEVDPRAAVSDLGVDSVMTVALRQKLQGAMNIKVPPTLTWNHPTVVHLVEWFYTKL	4
KXX82176-PP	*MMYC01_201685*	*Madurella mycetomatis* mm55	Betaenone	RVIEHAFADTLRAILQVTTSDEDLMASRSNEIGLDSLVSVNIRSWFLRHLQVGIPVLRIMSNNTMRSLVQLA	7
KXX82904-PP	*MMYC01_200634*	*Madurella mycetomatis* mm55	Solanapyrone	AATVALVQDAIVRAVAEMLFVDVEGIDPAKSVAELGVDSLIAAELRSWFLKALGASISMLDLLD	7
RYP01991-PP	*DL764_005996*	*Monosporascus ibericus* CBS 110550	Betaenone	AQLRRILQVSTTDDDLMMMRGADLGFDSLISVDVRSWFLKNFQVSIPVLKIM	7
KAF2806012-PP	*BDZ99DRAFT_539327*	*Mytilinidion resinicola* CBS 304.34	Solanapyrone	ARAVELVTAALKTTIAGMLLMDESTVNAARAVSDHGVDSLIATEFRNWLLLALGAKISMVDLLDPRTTINALAAKV	7
KAI1122634-PP	*F5Y10DRAFT_281543*	*Nemania abortiva* FL1152	Mellein	DRQQWLTTKIRECIAAVLMIPDIDEIDIRKPLSDFGVDSVMTVALRQKLQMAFKIKVPATLTWNHPTVNHLVAWF	4
KAJ4366994-PP	*N0V83_007524*	*Neocucurbitaria cava* IMI 356814	Mellein	IRQCLSTVLHLDAEDIDERSAIADLGVDSVMTVALRQQLQKEMGVTVPPTLTWNCPTMEHLVE	4
GME33674-PP	*NpNSSI1_00002891*	*Neofusicoccum parvum* NSSI1	Mellein	AAGPELKAWLNVRIRECIAAVLMIGDIEEVDPRAAVSDLGVDSVMTVALRQKLQGAMNVKVPPTLTWNHPTVVHLVEWF	4
KAL1637766-PP	*SLS56_000321*	*Neofusicoccum ribis* M1-105	Mellein	ESRPAAGPELKAWLNVRIRECIAAVLMIGDIDEVDPRAAVSDLGVDSVMTVALRQKLQGAMNVKVPPTLTWNHPTVVHLVEWF	4
EGO58521-PP	*NEUTE1DRAFT_77860*	*Neurospora tetrasperma* FGSC 2508	Solanapyrone	RTVALVQDAITKVVAEMLFVDVESIDPAKSVAELGVDSLIAAELRNWFIQALGTNISMLDLLD	7
KAL1608443-PP	*SLS60_003385*	*Paraconiothyrium brasiliense* M42-189	Mellein	IRECVGAVLHMDVEDIDVRAAIADLGVDSVMTVSLRQKLFSVLGVKVPPTLLWKEPTVGHLVEW	4
KAI9158144-PP	*HJFPF1_06134*	*Paramyrothecium foliicola* TJWQPF1	Mellein	IRECIASVLLMGDIDDIDSRMPVADLGVDSVMTVALRQKLQTALNVKVPPTLTWNHPTVNHLV	4
KAI9163194-PP	*HJFPF1_04793*	*Paramyrothecium foliicola* TJWQPF1	Monacolin K	AKTLDEVRDIVIDGLSQKMRGILHIPADEEVNVTAPLIDQGVDSLGAITVGSWFSKTLMIDIPLLRVVSGASIAELAEEAAGRL	8
KAI9163910-PP	*HJFPF1_05542*	*Paramyrothecium foliicola* TJWQPF1	Monacolin K	ALQLTNADELNLATPLIDQGVDSLSAVTIASWFSKNLNIDIPLLKILGGASVNDLVD	8

OAG05415-PP	*CC84DRAFT_1243646*	*Paraphaeosphaeria sporulosa* AP3s5-JAC2a	Mellein	KIRECIGAVLHMDVEDIDVRAAIADLGVDSVMTVSLRQKLLSVLGIKVPPTLLWKEPTVGHLVEW	4
OAG05883-PP	*CC84DRAFT_1244536*	*Paraphaeosphaeria sporulosa* AP3s5-JAC2a	Solanapyrone	GRKLVIAATKDKMSGLLGIPVGTIQENKSVAEYGVDSLIAVELRNWFLTTFKTAIPLLRLLDE	7
KAH7092165-PP	*FB567DRAFT_434875*	*Paraphoma chrysanthemicola* MPI-SDFR-AT-0120	Mellein	IDVQIRECVATVMKIVDIEEIDPRVPLADFGVDSVMTIALRQKLQSSLKVKVPQTLMWNYPTVAAMSEW	4
KAH7094847-PP	*FB567DRAFT_543251*	*Paraphoma chrysanthemicola* MPI-SDFR-AT-0120	Mellein	SRVLHLDVEEIDDRTAIADLGVDSVMTVALRQQLQKAMSITVPPTLTWSCPTVIHLVD	4
KAH6006611-PP	*HBI84_063510*	*Parastagonospora nodorum* 16FG159	Mellein	ADLKPWLDVKIRECVALVMGVEDIEEIDTRVPLSDYGVDSIMTIALRQKLQSKLKIKVPQTLMWNYPTVSAMVGWF	4
KAH6012763-PP	*HBI84_033690*	*Parastagonospora nodorum* 16FG159	Betaenone	SQADITKVVQQAYCAQLRRILQVSTADEDLMMMRGVDLGFDSLLSVDVRSWFLKNFRVSIPVLKIM	7
CRL28213-PP	*PCAMFM013_S026g000078*	*Penicillium camemberti* FM013	Solanapyrone	TTSSIATTVATMLLMDISAVNTARAVSDHGVDSLIATELRNWFHLALGSKIGMVDLLDPRTSINALAAKV	7
KAJ6042278-PP	*N7446_013344*	*Penicillium canescens* IBT 15451	Fumagillin	AITVVGARLAKQLRLTGGLDSARPLSYYGLDSLAAVELRNWVRMTFGVELTT	5
KAJ5201517-PP	*N7498_006180*	*Penicillium cinerascens* IBT 15544	Solanapyrone	IATTVATMLLMDISAVNTARAVSDHGVDSLIAAELRNWFHLALRSKISMVDLLDPRTSINALAAKV	7
KAJ5365630-PP	*N7517_008516*	*Penicillium concentricum* IBT 3081	Solanapyrone	TSQWIATTIATMLLVDISSINTARAVTDHGLDSLIAVELRNWFHLALGFKVTMLDLLDSRTSIKSLAARVV	7
KGO76199-PP	*PITC_048990*	*Penicillium italicum* PHI-1	Solanapyrone	DKSLTLVCDAIIGAMAAMLSISPASISLSKGVADYGVDSLIAAELRNWFSQAFRANISLLDLLDAHTSIRQLA	7
KAJ580654-PP	*N7503_004145*	*Penicillium pulvis* IBT 33274	Monacolin K	DEVRAIILEGVSAKIRGALQVAASDDINLDSPLVDQGVDSLSAVTIGTWFSKNLGVDVPLLKILGGASVSDLVE	8
KAJ5877506-PP	*N7455_000971*	*Penicillium solitum* IBT 25940	Solanapyrone	DRNKVEEMVTGAIRNTVAEMLFIDASGVDPTRTVAEYGVDSLIAAELRNWFNTSFMADISMLDLLDTRMSMKALAK	7
KAJ5861069-PP	*N7529_008379*	*Penicillium soppii* IBT 18220	Fumagillin	AAVRTAAIVVVGSRLTKQLRLTDALDPARPLSYYGLDSLAAVELRNWVRITLGVEVTTLDVMNAASLGEFCDKVVG	5

OQE31496-PP	*PENSTE_c001G10122*	*Penicillium steckii* IBT 24891	Monacolin K	DEVRAIILEGISAKIRVALQVAASDDINLNSPLVDQGVDSLSAVTIGTWFSKNLSVDVPLLKILGGASVSDLVE	8
KAJ5883737-PP	*N7473_010623*	*Penicillium subrubescens* IBT 31985	Solanapyrone	ETVAFVLGAITRAVAEMLFVDVESIDPAKSLADLGVDSLIAAELRNWFLQALGVNISMLDLL	7
ETS75984-PP	*PFICI_12928*	*Pestalotiopsis fici* W106-1	Mellein	LRECLASVLKIDDIEEIDDRVALTDIGVDSVMTIVLRQKLQSVLKIKVPQTLTWNYPTVVAMVDWF	4
ETS86665-PP	*PFICI_00493*	*Pestalotiopsis fici* W106-1	Solanapyrone	SATELVSEAISNAVAEMLFIDASGVNSAMTVAAHGVDSLIAAELRNWFHQAFKFKISMPDLLDAHTSISELAAKV	7
KAK3202464-PP	*GRF29_161g1276295*	*Pseudopithomyces chartarum* AGR01	Mellein	KIRECIGAVLHMDIEDIDVRAAVADLGVDSVITVALRQKFQNVLGVKVPPTLMWKEPTVEHLVKWFNKQ	4
TLD06378-PP	*PgNI_08287*	*Pyricularia grisea* NI907	Monacolin K	AATDMEEVQAIISETLSEKMRGVLHIPAEESVNASAPLLDQGIDSLGAITVASWFSKQLLVEIPILRVLSGASIEELAAEGASRL	8
TLD10904-PP	*PgNI_06555*	*Pyricularia grisea* NI907	Betaenone	RVVKSAFGAQLRRTIQVSTADEDLMTMRGIELGFDSLISVDLRSWFFRTFQVSIPVLKIMAN	7
QBZ60730-PP	*PoMZ_07672*	*Pyricularia oryzae* MZ5-1-6	Monacolin K	EEVQDIISQTLSEKMRGVLHIPPEESVNASAPLLDQGIDSLGAITVASWFSKQLLVEIPILRVLSGASIEELAAEGASR	8
TLS27255-PP	*PpBr36_04254*	*Pyricularia pennisetigena* Br36	Monacolin K	MEEVQAIISESLSEKMRGVLHIPPEESVNASAPLLDQGIDSLGAITVASWFSKQLLVEIPILRILSGASIEELAAEGASR	8
CZT21184-PP	*RCC_07046*	*Ramularia collo-cygni* URUG2	Boydins	SMTRDHATQVVLEEISVKLEEMFGKSRIDHSRDFSASGVDSLVAIELRNWLGRQTGLAVTVMDLMQASSIHDFASEMA	
CZT22763-PP	*RCC_08468*	*Ramularia collo-cygni* URUG2	Betaenone	TEQEIHRVLQQAFAAQLRRILFIKLSDEEIMESASSNLGLDSLIAVDIRSWILKTFGVNVPVLKIMS	7
KAH8172381-PP	*LIA77_06636*	*Sarocladium implicatum* TR	Boydins ?	ASKTIQEARQTIISVFTDHLGKFLQIPTTEIDTSRPLFTYGVDSLVAMEMRNWIATEFKADLGLFDITAHVPITDLVGKIAEK	
KEZ45498-PP	*SAPIO_CDS1819*	*Scedosporium apiospermum* IHEM 14462	Boydins	TEEVDVSKDLTAHGVDSLVAVEIRNWLVAQTGVELSILDLIQSKSLTELAELVAVQ	

SaurPKS-PP	*SJVH01000046.1:52298-61732*	*Scedosporium aurantiacum* MUT 6114	Boydins	TDEVDVSRDLTAHGVDSLVAVEIRNWLAAQTGIELSILDLIQSNSLTELAELVAARL	
SboydPKS-PP	*NJFT01000058.1:150537-159137*	*Scedosporium boydii* IHEM 23826	Boydins	TEEVDVSKDLTAHGVDSLVAVEIRNWLVAQTGIELSILDLIQSKSLTELAELVAVQ	
SdehoogPKS-PP	*PGIR01000017.1:40098-48674*	*Scedosporium dehoogii* UA120008799-01/4	Boydins	TEEVDVSKDLTAHGVDSLVAVEIRNWLVAQTGIELSILDLIQSKSLAELAELVAVQ	
ROT40784-PP	*SODALDRAFT_396446*	*Sodiomyces alkalinus* F11	Solanapyrone	EALALVTRAISATVAGMLSIDASGVSPAKTVADYGVDSLIAAELRNWFNTAFRANISLLDLLDTHTSMEKLAGYVVE	7
KEY69314-PP	*S7711_01764*	*Stachybotrys chartarum* IBT 7711	Mellein	ARPSDPQQLEKWLNLKIRASIAAVLLMNDIDDIDARTPVADLGVDSVMTVALRQKLQAVLKVKVPPTLTWNHPTVNHLV	4
KFA68718-PP	*S40285_08959*	*Stachybotrys chlorohalonatus* IBT 40285	Mellein	ARPSDPQQLEKWLNLRIRESIAAVLLMNDVDDIDARTPVADLGVDSVMTVALRQKLQAVLKVKVPPTLTWNHPTVNHLVAW	4
KAH7312479-PP	*B0I35DRAFT_356106*	*Stachybotrys elegans* MPI-CAGE-CH-0235	Mellein	QELEKWLNVKIRECIAAVLLIADIDDIDVRMPVSDLGVDSVMTVALRQKLQAVLKVKVPPTLTWNHPTVNHLV	4
KAH7322416-PP	*B0I35DRAFT_389319*	*Stachybotrys elegans* MPI-CAGE-CH-0235	Betaenone	RVVTSTFCNQLRSILQVTTSDVDLLVARSSEIGLDSLVSLDLQTWILKNLQVNIPVFKIMGNHAMSQLVEYIVE	7
KAH7324402-PP	*B0I35DRAFT_161335*	*Stachybotrys elegans* MPI-CAGE-CH-0235	Betaenone	LIQERFAVQLRKMLQMTTPDEELMGMRSSEIGLDSLVSVDIRGWFLKNFKVGIPVLKIMGNEAMSSLVQHAAE	7
EED16637-PP	*TSTA_017120*	*Talaromyces stipitatus* ATCC 10500	Betaenone	KAIEHAFASQLRRVLQVTSSDEEIMAAHGADIGLDSLVSVDIRSWFLNTLQASIPVLKIMGNESMSSLVSYAV	7
KAI4201996-PP	*LQ350_002901*	*Teloschistes chrysophthalmus* LIQ78T CHR-2	Solanapyrone	VQKAITDVVAGMLFIDAESIDPAKSVAELGVDSLIAAELRNWFLQALGTGISMLDLLD	7
KAH6866263-PP	*B0T10DRAFT_451978*	*Thelonectria olida* MPI-CAGE-CH-0241	Mellein	LCQCLGEILKINEIDEIDPRIALTDIGVDSVMTIVLRQKLQSVLKVKVPQTLTWNYPTVSAMVDW	4
AEO58317-PP	*MYCTH_49393*	*Thermothelomyces thermophilus* ATCC 42464	Betaenone	VKSAFAAQLRRILQISTGDDDMMNMRSIDLGLDSLISVDIRSWFLKNLQVSIPVLKIM	7

TPX07903-PP	*E0L32_010358*	*Thyridium curvatum* D216	Boydins	ATACILGELTKELIRMFGAIEIDPSKDLAFHGVDSLVAVEIRNWLVAQTAVELPGSEAARAYLSRVGKNFPFRNNRL	
KAF2251179-PP	*BU26DRAFT_277929*	*Trematosphaeria pertusa* CBS 122368	Solanapyrone	LKQRAHAVELVTGGITATVASMLLLDPVTVNAARAVSDHGVDSLIAAELRNWFHAALGSKISMVDLLDPRTSISALAARV	7
KAF2253723-PP	*BU26DRAFT_601789*	*Trematosphaeria pertusa* CBS 122368	Mellein	IKECIGAVLHLDVEEIDDRAAIADLGVDSVMTVALRQKLQQVMGVKVPPTLTWNHPTVGHLVE	4
KAK4172803-PP	QBC36DRAFT_221937	*Triangularia setosa* CBS 892.96	Solanapyrone	RASTVEFVEDAITRVVADMLFVDVEGIDPVKSVADLGVDSLIAAELRNWFLQALGTNISMLDLLD	7
KAK4173766-PP	*QBC36DRAFT_51729*	*Triangularia setosa* CBS 892.96	Solanapyrone	TSTVALVTEGVTQTVAAMLFIDASTVDPAKSIAEHGVDSLIAAELRSWFHQVLKTNLKMGELLDAQTSIRALAENIV	7
KAK4194563-PP	*QBC40DRAFT_319083*	*Triangularia verruculosa* CBS 315.58	Solanapyrone	AGGERAKTVEFLEAAITEVVADMLFVDVEGIDPAKSVADLGVDSLIAAELRSWFLQALGTNISMLDLLD	7
KAK4085595-PP	*Triagg1_585*	*Trichoderma aggressivum f. europaeum* CBS 100526	Mellein	TDPQEREKWVDVRVRECIARVLMMDDIDDIGPRMPLLDLGMDSVMTVALRQTFQTVFKIKVPLTLTWNHPTVKHLV	4
KAH6638668-PP	*BKA67DRAFT_615055*	*Truncatella angustata* MPI-SDFR-AT-0073	Mellein	LRECLASILKIADIEDIDPRVALSDIGVDSVMTIVLRQKLQSVLKVKVPQTLTWNYPTVVAMVDW	4
KAH6651430-PP	*BKA67DRAFT_537308*	*Truncatella angustata* MPI-SDFR-AT-0073	Solanapyrone	ALVEEGITTTVAEMLFNGKSNVNLAKSVADHGVDSLIAAELRNWFHQALRTNLLMLDLLDAHTSIKALAESVVE	7
KAH6653270-PP	*BKA67DRAFT_606676*	*Truncatella angustata* MPI-SDFR-AT-0073	Mellein	KLQEWLTTRIRECIAAVLMITDIEDIDPRMPVSDLGVDSVMTVALRQKLQTVLKVKVPPTLTWNHPTVNHLVAWFT	4
KAH6658993-PP	*BKA67DRAFT_3389*	*Truncatella angustata* MPI-SDFR-AT-0073	Betaenone	VKGAFATQLRNILQMTTSDEDLMASRSNEIGLDSLVSVDIRSWFLDKLQVSIPVLKIMGNDTMESLVQHAVET	7
KAI3330051-PP	*F4824DRAFT_480428*	*Ustulina deusta* IL1129	Mellein	KGDRQQWLTTKIRESIAAVLMISDIDEIDIRKPLSDFGVDSVMTVALRQKLQMAFKIKVPATLTWNHPTVNHLVKFFH	4
RDL31023-PP	*BP5553_09812*	*Venustampulla echinocandica* BP 5553	Fumagillin	RAAVRSAAVTAVGARLAKQLRLTVALDPARPLLQYGMDSLAAVELRNWVRMILGVELTTLDVANAASLGELCDKVTG	5

KAF2229154-PP	*EV356DRAFT_496446*	*Viridothelium virens* Tuck. ex Michener	Mellein	AKTSELKPWLDVKIRECIASLLKIGDIEEIDRRV ALSDVGVDSVMTIALRQKLESMLKVKVPQTLIWN HPTVVHMVEWF	4
KAF2237104-PP	*EV356DRAFT_565184*	*Viridothelium virens* Tuck. ex Michener	Solanapyrone	ASTVELVTGEITKTIAEMLFIDAGNVNPSKSVAD HGVDSLIAAELRNWLYQALGAKLPNLLDSETSIR MLAEQIV	7
KAF2275719-PP	*EI97DRAFT_467862*	*Westerdykella ornata* CBS 379.55	Solanapyrone	LIANAIAERTASLLMIPSESIGTQKSVAQYGVDS LIAVELRSWVALMFGVSIPLLKLLDEKISIRELG GWIA	7
KAI0449065-PP	*F5B21DRAFT_520838*	*Xylaria acuta* CBS 122032	Betaenone	AELLKIVQDTFAAQLRNILQMSTPDEDLMAMRSS EIGLDSLISVDVRAWFLRHYHVSIPVLKIMGNDT MANVAKHAVE	7
KAI0451189-PP	*F5B21DRAFT_528181*	*Xylaria acuta* CBS 122032	Solanapyrone	VAFVEAAINTTVAEMLFVDSKGIDQAKSVASHGV DSLIAAELRNWFHQALGANISMLDLLDPNT	7
KAI0455320-PP	*F5B21DRAFT_503375*	*Xylaria acuta* CBS 122032	Betaenone	IEGAFATQLRVVLQVSTSDQDLMASRGNEIGLDS LISVDIRSWFLKHLQVNVPVLKIMSNNTMASLVQ YT	7
KAI0973328-PP	*F4678DRAFT_426463*	*Xylaria arbuscula* CBS 124340	Betaenone	QEAYQVIENAFAAQLRVVLQVSTSDQDLMASRGN EIGLDSLISVDIRSWFLKHLQVNVPVLKIMSNNT MASLVQFT	7
KAI0973631-PP	*F4678DRAFT_486812*	*Xylaria arbuscula* CBS 124340	Solanapyrone	LSAAIELVANATAHTVAQMLFIDASAVETAKSVG HYGVDSLVAAELRSWLNLTFGADVDVLEMLDT	7
KAI0502734-PP	*F5B22DRAFT_640677*	*Xylaria bambusicola CBS 139988*	Solanapyrone	SRIVDSVTDAISAAIAELLFVDIANVNSAKTVAD HGVDSLIAAELRNWLYQALGANINMQELLDTRTS IESLAG	7
KAI1755490-PP	*F4782DRAFT_488724*	*Xylaria castorea* CBS 124033	Betaenone	HVIEGAFATQLRVVLQMSTSDQDLMAARGNEIGL DSLISVDIRSWFLKHLQVNVPVLRIMSNNTMASL IQFAI	7
KAI1758243-PP	*F4782DRAFT_536832*	*Xylaria castorea* CBS 124033	Mellein	KGDRQQWLTTKIRECIAAVLMISDIDEIDIRKPL SDFGVDSVMTVALRQKLQMAFKVKVPATLTWNHP TVNHLVAWFS	4
KAI0862706-PP	*F4860DRAFT_523077*	*Xylaria cubensis* CBS 116.85	Betaenone	HVIEGAFATQLRVVLQVSTSDQDLMASRGNEIGL DSLISVDIRSWFLKHLQVNVPVLKIMSNNTMASL IQFAI	7
KAI0864625-PP	*F4860DRAFT_529190*	*Xylaria cubensis* CBS 116.85	Mellein	PVEKGDRQQWLTTKIRECVAAVLMIPDIDEIDVR KPLSDFGVDSVMTVALRQKLQMAFKVKVPATLTW NHPTVNHLVGWFS	4

KAI0545024-PP	*F4679DRAFT_576454*	*Xylaria curta* CBS 114988	Solanapyrone	EAEQLEAVRLVANEIARTVAQMLFIDASRMDTAKSVAQYGMDSLLAAELRNWLNSTFGIDVDMLEILDTET	7
KAI0553962-PP	*F4679DRAFT_598225*	*Xylaria curta* CBS 114988	Mellein	DRQQWLTTKIRECVAAVLMISDIDEIDIRKPLSDFGVDSVMTVALRQKLQMAFKVKVPATLTWNHPTVNHLVAWFS	4
KAI0534672-PP	*GGR58DRAFT_505021*	*Xylaria digitata* CBS 161.22	Solanapyrone	IVAFAVRAITTAVAQMLFIDTEDVDPGRSVADHGVDSLIAAELRTWFQQALGANISMLGLLDPATTIRALAEQI	7
KAI0535679-PP	*GGR58DRAFT_503995*	*Xylaria digitata* CBS 161.22	Solanapyrone	VKRAITSTVAEMLFIDPLGVNPSKSVAGHGVDSLIAAELRNWFHQAFGINISMLELLDART	7
KAI0539112-PP	*GGR58DRAFT_266177*	*Xylaria digitata* CBS 161.22	Mellein	ERADRQQWLTTKIRECIAAVLMISDIDEIDIRKPLSDFGVDSVMTVALRQKLQMAFKVKVPATLTWNHPTVNHLVAWF	4
TRX95554-PP	*FHL15_003512*	*Xylaria flabelliformis* G536	Mellein	PVEKGDRQQWLTTKIRECVAAVLMMPDIDEIDVKKPLSDFGVDSVMTVALRQKLQMAFKVKVPATLTWNHPTVNHLVAWFS	4
RWA05644-PP	*EKO27_g9461*	*Xylaria grammica* IHI A82	Mellein	KGDRQQWLTTKIRECIAAVLMISDIDEIDIRKPLADFGVDSVMTVALRQKLQMAFKIKVPATLTWNHPTVNHLVAWF	4
RWA10608-PP	*EKO27_g4504*	*Xylaria grammica* IHI A82	Betaenone	EAYHVIESAFATQLRVVLQVSTSDQDLMASRGNEIGLDSLISVDIRSWFLKHLQVNVPVLKIMSNNTMSSLIQYAI	7
TGJ78773-PP	*E0Z10_g9991*	*Xylaria hypoxylon* DSM 108379	Solanapyrone	VMDGIMRTIAEMLFIDTVNVNPSKTVAEQGVDSLIAAELRNWFHQALRTNIHN	7
TGJ81633-PP	*E0Z10_g7123*	*Xylaria hypoxylon* DSM 108379	Mellein	KGDRQQWLTTKIRECIAAVLMISDIDEIDIRKPLSDFGVDSVMTVALRQKLQMAFKIKVPATLTWNHPTVNHLVAWF	4
TGJ86695-PP	*E0Z10_g2067*	*Xylaria hypoxylon* DSM 108379	Solanapyrone	AQDKTQSLVTDAIVGTVASMLSIEVSNISTAKPVAHYGVDSLIAAELRNWFTLAFRTDIPMLDVLDLHNSIEKLAKMVVE	7
KAI1821332-PP	*F4861DRAFT_542029*	*Xylaria intraflava* YMJ 725	Solanapyrone	LVVHGISIAIAEMLFVDAGNINPNKTVAEHGVDSLIAAELRNWFHQALRVDLKNLLDSETSIRALAEHIVDK	7
KAI8944329-PP	*F4801DRAFT_595032*	*Xylaria longipes* CBS 148.73	Mellein	VEKADRQQWLTTKIRECIAAVLMISDIDEIDIRKPLSDFGVDSVMTVALRQKLQMAFKVKVPATLTWNHPTVNHLVAWF	4

KAI8949604-PP	*F4801DRAFT_396144*	*Xylaria longipes* CBS 148.73	Betaenone	QEAYHVIEAAFATQLRVVLQVSTSDQDLMASRGNEIGLDSLISVDIRSWFLKHLQVNVPVLKIMSNNTMASLVQFT	7
KAI8951103-PP	*F4801DRAFT_578840*	*Xylaria longipes* CBS 148.73	Solanapyrone	AERSKTTMLVERVIKEAVADVLFIEADGVLPSKSIADHGLDSLVAAELRGWFIQALGVNVGTAELLDQSKTISQMAADMV	7
KAF2963728-PP	GQX73_g9837	*Xylaria multiplex* DSM 110363	Solanapyrone	AARDKTRSLVAGAIVGTVASLLSIEVSNVSTAKPVAHYGVDSLIAAELRNWFALVFRTDIPMLDVLDMHNTIEKLAELV	7
KAF2969744-PP	GQX73_g3823	*Xylaria multiplex* DSM 110363	Mellein	AEKADRQQWLTTKIRECIAAVLMISDIDEIDIRKPLSDFGVDSVMTFVLRQKLQMTFKVKVPATLTWNHPTVNHLVAWF	4
KAI0400032-PP	*F4802DRAFT_610433*	*Xylaria palmicola* CBS 124036	Mellein	PADKGDRQQWLTAKIRECIAAVLMISDLDEIDIRKPLSDFGVDSVMTVALRQKLQMAFKIKVPATLTWNHPTVNHLVAWFS	4
KAI0402316-PP	*F4802DRAFT_608818*	*Xylaria palmicola* CBS 124036	Solanapyrone	ADQKETTAFVEAAIARTVAEMLFSDADSVDPTKSVANHGVDSLIAAELRNWFYQALGTDISMLDLLD	7
KAI0406831-PP	F4802DRAFT_612982	*Xylaria palmicola* CBS 124036	Solanapyrone	RSKTVDFITGAISTAVASLLFVDISNVNPARTVADHGVDSLIAAELRNWFHQALRANIDMQALLDAKT	7
KAH8160526-PP	*CIB48_g7712*	*Xylaria polymorpha* DSM 105756	Mellein	APVEKGDRQKWLTTKIRECIAAVLMISDIDEIDTRKPLADFGVDSVMTVALRQKLQMA	4
KAI0438208-PP	*F4803DRAFT_569782*	*Xylaria telfairii* CBS 121673	Mellein	GDRQQWLTTKIRECIAAVLMISDIDEIDIRKPLVDFGVDSVMTVALRQKLQMAFKVKVPATLTWNHPTVNHLVAWF	4
KAI0438847-PP	*F4803DRAFT_569116*	*Xylaria telfairii* CBS 121673	Solanapyrone	SITVAEMLFVDSEGIDQAKSVASHGVDSLIAAELRNWFHQALGANISMLDLLDPNT	7
KAI1298194-PP	*F5Y03DRAFT_386702*	*Xylaria venustula* FL0490	Betaenone	KEAYSAQLRKTLQISTADDDLMMMRGAEIGFDSLVSVDVRSWFLRNFRVSIPVLKIM	7
KAI1303963-PP	*F5Y03DRAFT_358607*	*Xylaria venustula* FL0490	Betaenone	QEAYQVIESAFAAQLRVVLQVSTSDQDLMASRGNEIGLDSLISVDIRSWFLKHLQVNVPVLKIMSNNSMASLVQFT	7
KAF2180125-PP	*K469DRAFT_640404*	*Zopfia rhizophila* CBS 207.26	Solanapyrone	TTNAIIERTAGLLMIPVESIEAGMSVAEYGVDSLIAVELRNWVALMFETSIPLLKLLD	7
KJX98396-PP	*TI39_contig413g00002*	*Zymoseptoria brevis* Zb18110	Boydins	ATHIDTSKDFSECGVDSLVAIELRNWLRTQTGLDISVTGMMQASSIDDFAHEVAER	

Zpsdtrit-PP	*JYJD01002299.1:14950-22949*	*Zymoseptoria pseudotritici* ST04IR_5.5	Boydins	GATHIDTSKDFSECGVDSLVAIELRNWLRTQTGL DISVTGMMQASSIDDFAHEVAER	
EGP90777-PP	*MYCGRDRAFT_84380*	*Zymoseptoria tritici* IPO323	Boydins	GATHIDTSKDFSECGVDSLVAIELRNWLRTQTGL DISVTGMMQACSIDDFAHEVAER	
EGP91889-PP	*MYCGRDRAFT_67477*	*Zymoseptoria tritici* IPO323	Betaenone	TREEVHQALKQAFAAQMRKILFIKLSDDEVMESV SSTLGLDSLIAVDIRSWIMKTFSVNIPVLQIMS	7

PP: Phosphopantetheine.

Supplementary Table S4: Selected iterative PKSs exhibiting a functional ER domain and comparison with that of the ER domain of KEZ45498 and its orthologs in *Scedosporium* species

Protein Genbank accession number	Gene GenBank accession number	Fungal species and strain	Metabolite produced
CBF88289-ER	*ANIA_01036*	*Aspergillus nidulans* FGSC A4	Asperfuranone
EAU31921-ER	*ATEG_07659*	*Aspergillus terreus* NIH2624	Asperfuranone
GCB26577-ER	*AAWM_09462*	*Aspergillus awamori* IFM 58123	Fumonisin
RAK91539-ER	*BO79DRAFT_141520*	*Aspergillus costaricaensis* CBS 115574	Fumonisin
BCR95830-ER	*AKAW2_20770A*	*Aspergillus luchuensis* IFO 4308	Fumonisin
PYH58042-ER	*BO96DRAFT_499196*	*Aspergillus niger* (*Aspergillus lacticoffeatus*) CBS 101883	Fumonisin
RDK46058-ER	*M752DRAFT_332694*	*Aspergillus phoenicis* ATCC 13157	Fumonisin
PYI12621-ER	*BO78DRAFT_457054*	*Aspergillus sclerotiicarbonarius* CBS 121057	Fumonisin
RDH37115-ER	*BDQ94DRAFT_183969*	*Aspergillus welwitschiae* CBS 139.54b	Fumonisin
EMD62755-ER	*COCSADRAFT_343166*	*Bipolaris sorokiniana* ND90Pr	Fumonisin
KAF5242405-ER	*FANTH_8691*	*Fusarium anthophilum* NRRL 25214	Fumonisin
KAF5966031-ER	*FBULB1_11909*	*Fusarium bulbicola* NRRL 25176	Fumonisin
CCT73928-ER	*FFUJ_09241*	*Fusarium fujikuroi* IMI 58289	Fumonisin
KAF5711983-ER	*FGLOB1_4749*	*Fusarium globosum* NRRL 26131	Fumonisin
CZR47926-ER	*FPRO_13593*	*Fusarium proliferatum* ET1	Fumonisin
RBQ98724-ER	*FVER53263_00316*	*Fusarium verticillioides* BRIP53263	Fumonisin
UQC88619-ER	*CLUP02_14144*	*Colletotrichum lupini* IMI 504893	Zearalenone
PSN68227-ER	*BS50DRAFT_676138*	*Corynespora cassiicola* Philippines	Zearalenone
PSN68460-ER	*BS50DRAFT_676305*	*Corynespora cassiicola* Philippines	Zearalenone
ESU07825-ER	*FGSG_12126*	*Fusarium graminearum* PH-1 (*Gibberella zeae* PH-1)	Zearalenone
EKJ72969-ER	*FPSE_06865*	*Fusarium pseudograminearum* CS3096	Zearalenone
KAI1771028-ER	*F4818DRAFT_454916*	*Hypoxylon cercidicola* CBS 119009	Zearalenone
OAG15814-ER	*CC77DRAFT_1012953*	Alternaria alternata SRC1lrK2f	Alternapyrone
KAI4909447-ER	*J4E90_008144*	*Alternaria incomplexa* BMP 0042	Alternapyrone
KAI4642909-ER	*J4E93_006978*	*Alternaria ventricosa* BMP 2768	Alternapyrone
KAF4483323-ER	*CGGC5_v009630*	*Colletotrichum fructicola* Nara gc5	Alternapyrone
KAF3802434-ER	*GCG54_00003237*	*Colletotrichum gloeosporioides* Lc1	Alternapyrone

KAF9877573-ER	*CkaCkLH20_04708*	*Colletotrichum karsti* CkLH20	Alternapyrone
KAF6845034-ER	*CMUS01_00567*	*Colletotrichum musicola* LFN0074	Alternapyrone
KAJ0320293-ER	*Brms1b_003318*	*Colletotrichum noveboracense* PMBrms-1	Alternapyrone
KAJ0292463-ER	*CBS470a_002769*	*Colletotrichum nupharicola* CBS470	Alternapyrone
KAF5511154-ER	*CGCS363_v003160*	*Colletotrichum siamense* Cg363	Alternapyrone
KAK8246206-ER	*HDK90DRAFT_444923*	*Phyllosticta capitalensis* (*Guignardia mangiferae*) CBS 1[illegible]	Alternapyrone
KAK7523902-ER	*IWZ03DRAFT_339305*	*Phyllosticta citriasiana* CBS 123371	Alternapyrone
KAK7563603-ER	*IWX92DRAFT_439053*	*Phyllosticta citricarpa* CBS 122482	Alternapyrone
KAK8153700-ER	*IWX90DRAFT_495291*	*Phyllosticta citrichinensis* CBS 129764	Alternapyrone
KAK7615034-ER	*JOL62DRAFT_137903*	Phyllosticta paracitricarpa CBS 141358	Alternapyrone
TLD04408-ER	*PgNI_12145*	*Pyricularia grisea* NI907	Alternapyrone
QBZ61696-ER	*PoMZ_08651*	*Pyricularia oryzae* MZ5-1-6	Alternapyrone
EMF15815-ER	*SEPMUDRAFT_131426*	*Sphaerulina musiva* (*Mycosphaerella populorum*) SO22[illegible]	Alternapyrone
KAE8153725-ER	*BDV25DRAFT_136664*	*Aspergillus avenaceus* IBT 18842	BII-rafflesfungin
KAF2257594-ER	*CC78DRAFT_482977*	*Lojkania enalia* (*Didymosphaeria enalia*) CBS 304.66	BII-rafflesfungin
KAF2633120-ER	*BU25DRAFT_471359*	*Macroventuria anomochaeta* CBS 525.71	BII-rafflesfungin
KAJ5195620-ER	*N7498_009058*	*Penicillium cinerascens* IBT 15544	BII-rafflesfungin
KAJ9484383-ER	*VN97_g8996*	*Penicillium thymicola* DAOM 180753	BII-rafflesfungin
GKU02576-ER	*FIMFG217701_00010823*	*Fusarium langsethiae* MFG 217701	W493 B / W493 A
KAG8676086-ER	*FPOAC1_002082*	*Fusarium poae* DAOMC 252244	W493 B / W493 A
EKJ70677-ER	*FPSE_09187*	*Fusarium pseudograminearum* CS3096	W493 B / W493 A
KAJ4255490-ER	*NW762_009485*	*Fusarium torreyae* CF00136	W493 B / W493 A
SPJ85401-ER	*FTOL_11182*	*Fusarium torulosum*	W493 B / W493 A
CEI65813-ER	*FVRRES_02325*	*Fusarium venenatum* A3/5	W493 B / W493 A
KAJ0414844-ER	*BJY00DRAFT_318416*	*Aspergillus carlsbadensis* CBS 123894	Emericellamide
CBF87072-ER	*ANIA_02547*	*Aspergillus nidulans* FGSC A4	Emericellamide
GIC93056-ER	*Aud_009535*	*Aspergillus udagawae* IFM 46973	Emericellamide
KIA75545-ER	*HK57_00684*	*Aspergillus ustus* 3.3904	Emericellamide
KAK3292802-ER	*B0H64DRAFT_464367*	*Chaetomium fimeti* CBS 168.71	Emericellamide
EAQ87856-ER	*CHGG_04475*	*Chaetomium globosum* CBS 148.51	Emericellamide
KAK4461021-ER	*QBC42DRAFT_347542*	*Cladorrhinum samala* PSN324	Emericellamide
CRG90802-ER	*PISL3812_07847*	*Talaromyces islandicus* WF-38-12	Emericellamide

QGA20981-ER	*EYB26_008691*	*Talaromyces marneffei* 11CN-20-091	Emericellamide
EGX96624-ER	*CCM_01282*	*Cordyceps militaris* CM01	Beauveriolide
KEZ45498-ER	*SAPIO_CDS1819*	*Scedosporium apiospermum* IHEM 14462	Boydins
SapioPKS_2RF1-5-ER	*JOWA01000077.1:626416-629318*	*Scedosporium apiospermum* 2RF1-5	Boydins
SapioPKS_HDO1-ER	*MVOQ01000007.1:784566-787468*	*Scedosporium apiospermum* HDO1	Boydins
SaurPKS-ER	*SJVH01000046.1:52298-61732*	*Scedosporium aurantiacum* MUT 6114	Boydins
SboydPKS-ER	*NJFT01000058.1:150537-159137*	*Scedosporium boydii* IHEM 23826	Boydins
SdehoogPKS-ER	*PGIR01000017.1:40098-48674*	*Scedosporium dehoogii* UA120008799-01/4	Boydins

Supplementary Table S5: List of the primers used.

Aim and species	Nucleotide sequence (5' to 3')	Location on contig
**Verification of chromosomal rearrangement**		
Scedosporium dehoogii		
S.dehoog017-F1	CACCTTGAAACACCAGCACG	21,121 → 21,140
S.dehoog017-R1	AACTCAAGACAGATGCCGGG	22,405 → 22,386
S.dehoog017-F2	CGTCATTGCATTCGCCTCTG	21,409 → 21,428
S.dehoog017-R2	CCCACCCAGCATCATTCACT	23,373 → 23,354
S.dehoog017-F3	CGGACCTACAAGGCACGATT	22,901 → 22,920
S.dehoog017-R3	GGTGGAAGAAACGGCCAAAC	24,973 → 24,954
S.dehoog017-F4	GTGAGTCGTTGGTTCGAGGT	24,424 → 24,443
S.dehoog017-R4	ATACGAGCCATGACGCTCAG	25,495 → 25,476
Scedosporium minutisporum		
S.minu98-F1	CGACCTGCTACACAACCTGT	26,086 → 26,105
S.minu98-R1	GGAGAGGGTTCCCACAAAGG	27,466 → 27,447
S.minu98-F2	GTATCCTCAGCTCGTGTCGG	26,994 → 27,013
S.minu98-R2	CCTTGAGGACCATCCACCAC	30,175 → 30,156
S.minu98-F3	ACCTCGTGTTAGCTTCACCG	29,267 → 29,286
S.minu98-R3	GGGGGAAGTACCTTGTTCCG	32,421 → 32,402
S.minu98-F4	CTCACCTTGCTCACCCTCTG	32,031 → 32,050
S.minu98-R4	AAGAGATTTCCTGGCTGGGC	34,130 → 34,111
S.minu98-F5	AGATCGCTTTTGGAGGTGGG	33,479 → 33,498
S.minu98-R5	TTCTCCTCGGATTTGGTGGC	35,433 → 35,414
**Verification of contig assembly**		
Scedosporium minutisporum		
S.minu182-98-F1	GGGTCCACGGTTAATTGGGT	Node 182: 52,442 → 52,461
S.minu182-98-R1	CCGGAGCCATCTCCTTGTTT	Node 98: 443 → 424
Zymoseptoria brevis		
Z.brevis413-102-F1	CGCTACTACGAGCATAGGCG	Contig 413: 21,781 → 21,800
Z.brevis413-102-R1	ATCGCTCTCCCGAACACTTG	Contig 102: 5,231 → 5,250
Z.brevis102-4213-F1	AACATGATCGTCCTGGCTCC	Contig 102: 553 → 534
Z.brevis102-4213-R1	GCCCTGGAAGACAGCAAACT	Contig 4213: 373 → 354
Z.brevis4213-1153-F1	GGCGGCAATCGACTTAGAAT	Contig 4213: 11,722 → 11,741
Z.brevis4213-1153-R1	TGATGGCGTCCTGTATGTGG	Contig 1153: 3,147 → 3,166
Z.brevis1153-432-F2	CTCAGCGCGAACAAAGAAGG	Contig 1153: 789 → 770
Z.brevis1153-432-R2	CATTGGTTTTGCGGTCGGAG	Contig 432: 25,843 → 25,862
**RT-qPCR experiments**		
Scedosporium apiospermum SAPIO_CDS1823		
SAMMT-F	TCATCAGCTTCGCCCTTCAC	640,578 → 640,597
SAMMT-R	TTCGGCCCCATCTTGTTTGA	640,699 → 640,680
Scedosporium apiospermum SAPIO_CDS1826		
GGCT-F	AGAAGCAGCCGTGTCCAATC	648,367 → 648,386
GGCT-R	TTCATGTATCGCAGGCTCGG	648,449 to 648,466 and 648,553 to 648,554
Scedosporium apiospermum SAPIO_CDS1827		
MP-F	AATTGACCGTTGCTCCTCGT	649,447 → 649,466
MP-R	CCTTCGGGTGTTGAGAGAGG	649,558 → 649539
Scedosporium apiospermum SAPIO_CDS1830		

TrxR1830-F	GACGTGAATCCTGCCACTTT	662,114 → 662133
TrxR1830-R	ACCGAGAATGTCACCCTGTA	662,218 → 662199
Scedosporium apiospermum SAPIO_CDS1831		
MT-F	TTGGGCGAATAACCACGTCT	663,752 → 663,771
MT-R	TCCATCGGCTTTTACGGCTT	663,884 → 663,865
Scedosporium apiospermum SAPIO_CDS1832		
GST-F	TCAAACCACTGATCCTCGCC	665,235 → 665254
GST-R	ATCCTCTGCGGGTGTATCCT	665,334 → 665,315
